# Supplementary material for: G Protein-Coupled Receptor–Ligand Pose and Functional Class Prediction
Source: Int J Mol Sci. 2024 Jun 22;25(13):6876. doi: 10.3390/ijms25136876 (PMC11241240; doi:10.3390/ijms25136876)
Supplement: Supplementary file 1 [file ijms-25-06876-s001.zip › Szwabowski_IJMS_2024_SupplementalFigures.pdf]

GPCR Ligand Pose and Functional Class Prediction

Gregory L. Szwabowski [1], Makenzie Griffing [2], Elijah J. Mugabe [3], Daniel O'Malley[4], Lindsey N. Baker[5], Daniel L. Baker [6,*], and Abby L. Parrill [7,*]

Department of Chemistry, University of Memphis, Memphis TN 38152, USA

1 gszwabowski@gmail.com
2 makenziegriffing@outlook.com
3 elm4026@med.cornell.edu
4 domalley@memphis.edu
5 lindsey.baker@yale.edu
6 dlbaker@memphis.edu
7 aparrill@memphis.edu
* Correspondence: aparrill@memphis.edu; Tel.: 1-901-678-3371 (AP) or dlbaker@memphis.edu (DB)

SUPPLEMENTAL FIGURES

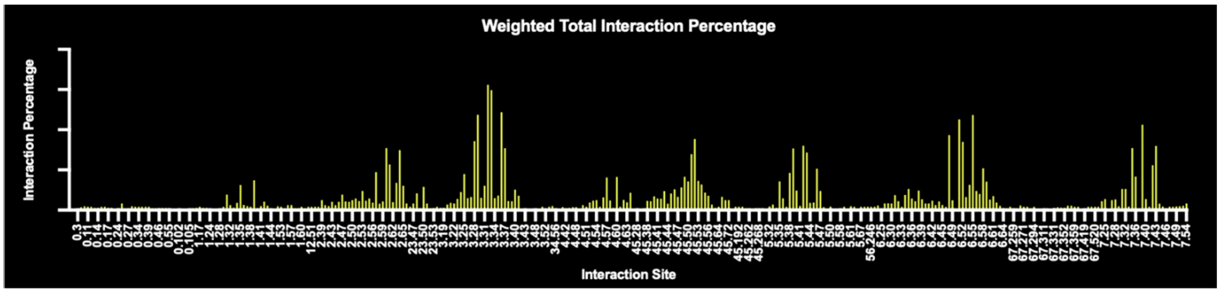

Supplemental Figure S1. The interaction percentages across all class A GPCR are shown, with the interaction site shown on the x-axis numbered with the BW numbering system. Interactions from the 311 ligand complex structures of 60 GPCR shown in Table S1 are represented.

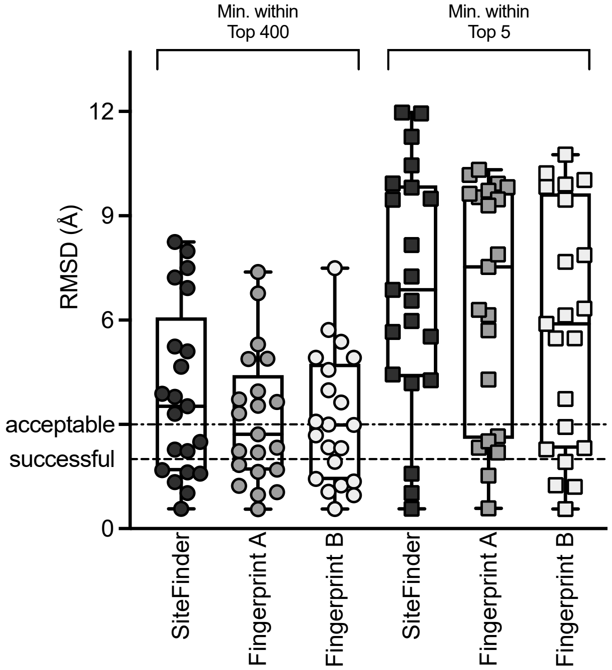

Supplemental Figure S2. Box and whisker plot showing best sampled and best scored RMSD values from each docking calculation used to compare automated site selection and site selection using global fingerprints.

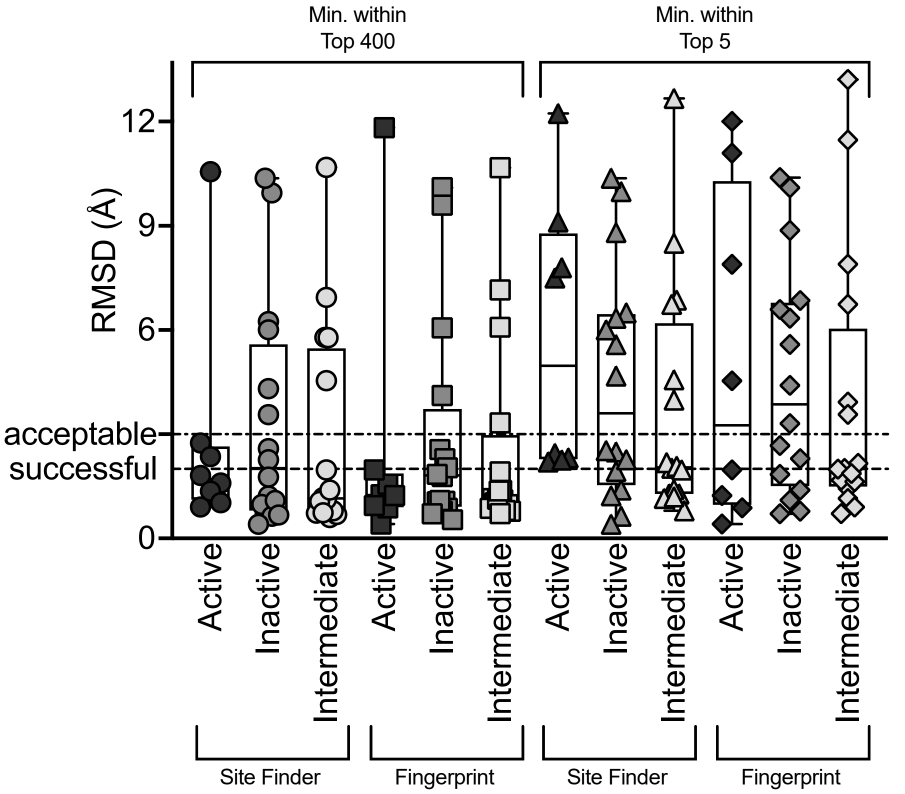

Supplemental Figure S3. Box and whisker plot showing best sampled and best scored RMSD values from each docking calculation used to compare automated site selection and site selection using activation state specific fingerprints.

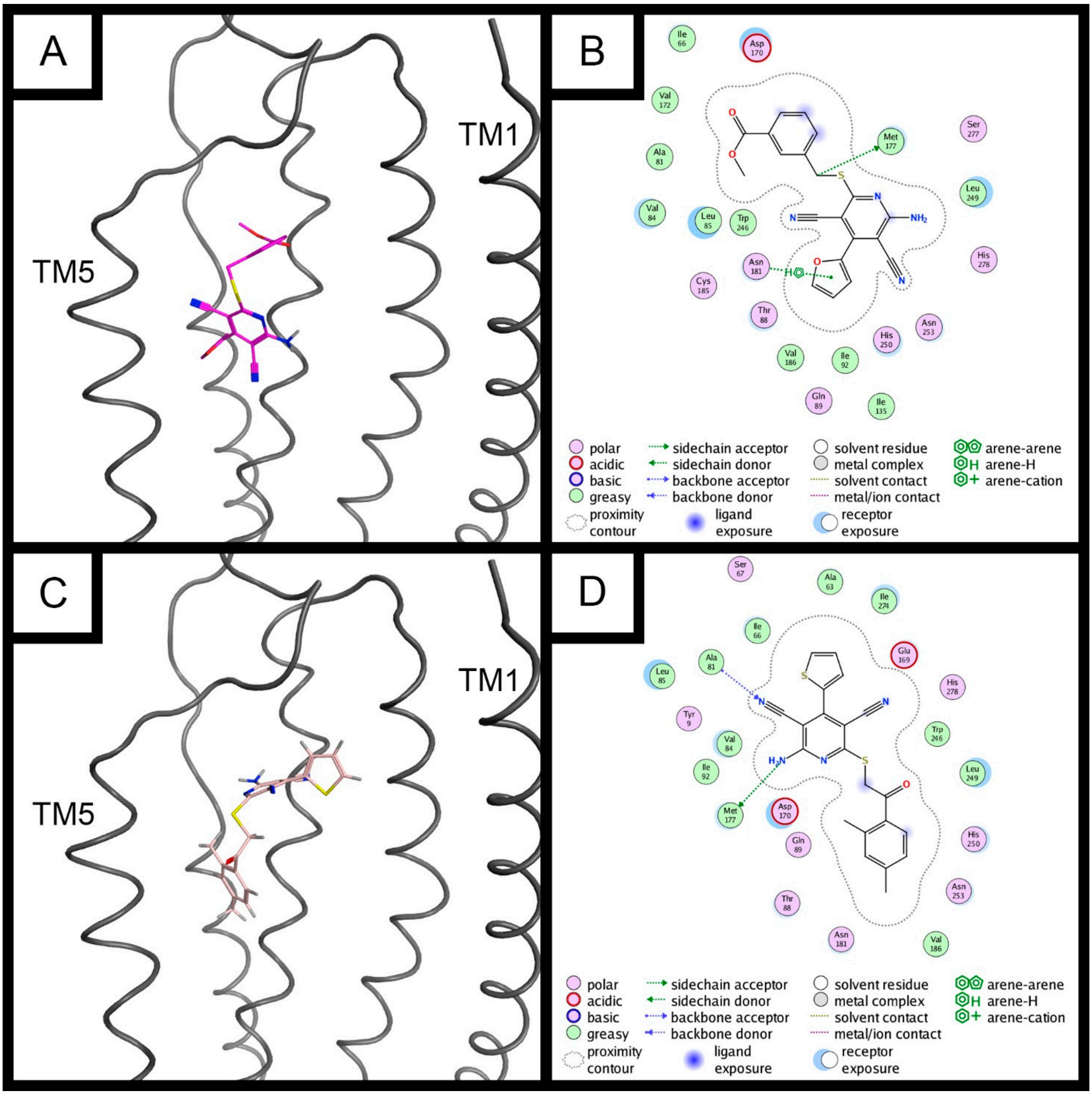

Supplemental Figure S4. Top scoring docked poses (A, C) and ligand interaction diagrams (B,D) of AA2AR antagonist Compound 10 (A,B) and AA2AR inactive 2-amino-6-((2-(2,4-dimethylphenyl)-2-oxoethyl)thio)-4-(thiophen-2-yl)pyridine-3,5-dicarbonitrile (C,D) in complex with the AA2AR best case active template homology model. Transmembrane domains 6 and 7 have been removed for visibility in panels A and C.

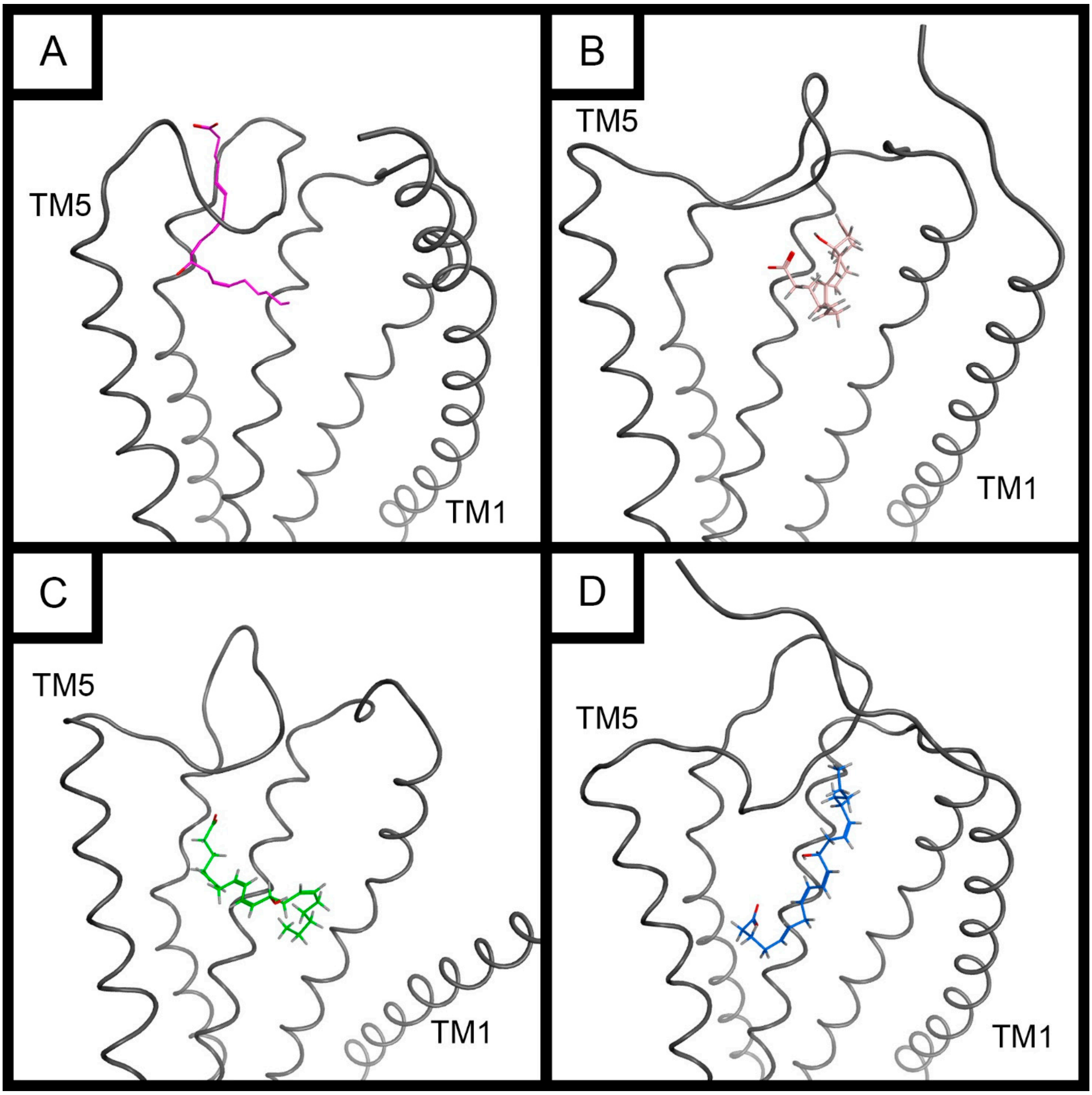

Supplemental Figure S5. Top scoring docked poses of GPR31 agonist 12(S)-HETE in complex with GPR31 modeled structures with transmembrane domains 6 and 7 removed for visibility. A) in-house homology model, B) GPCRdb active template homology model, C) GPCRdb inactive template homology model, D) AlphaFold homology model

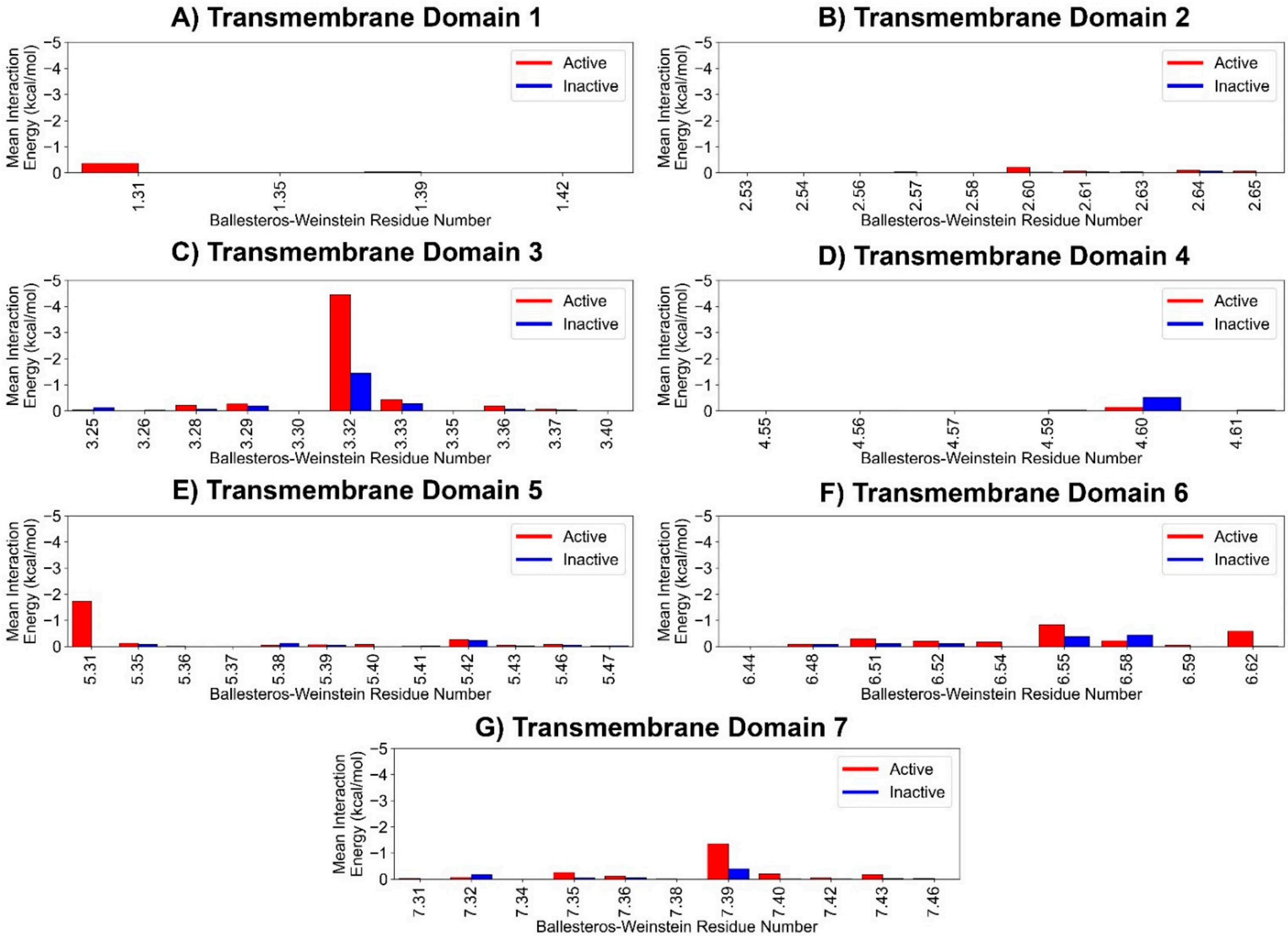

Supplemental Figure S6. Mean interaction energy for Ballesteros-Weinstein indexed residue positions in each transmembrane domain for residue positions possessing interactions in ≥ 10 complexes in the internal dataset.

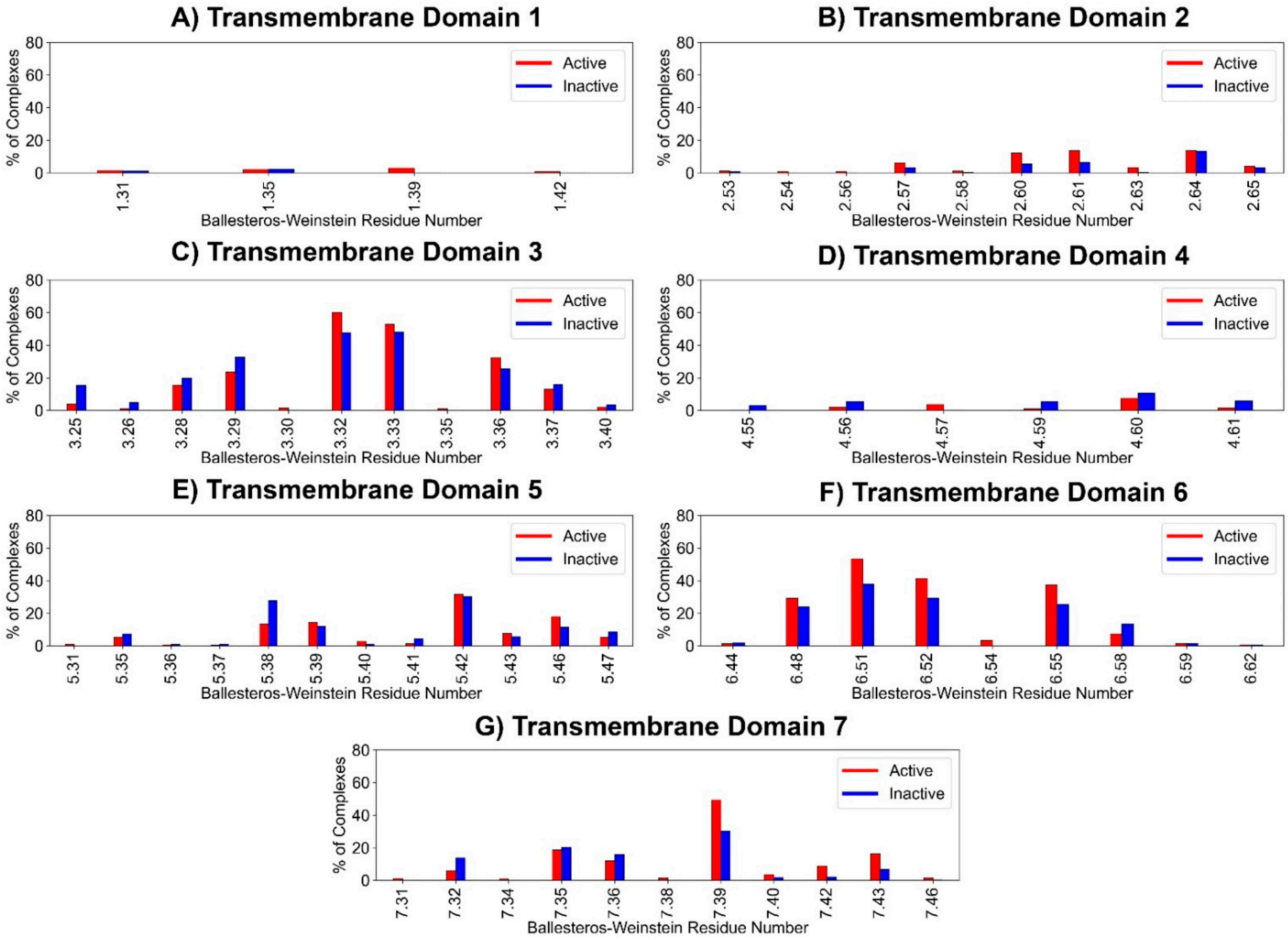

Supplemental Figure S7. Interaction percentages for Ballesteros-Weinstein indexed residue positions in each transmembrane domain for residue positions possessing interactions in ≥ 10 complexes in the initial dataset.

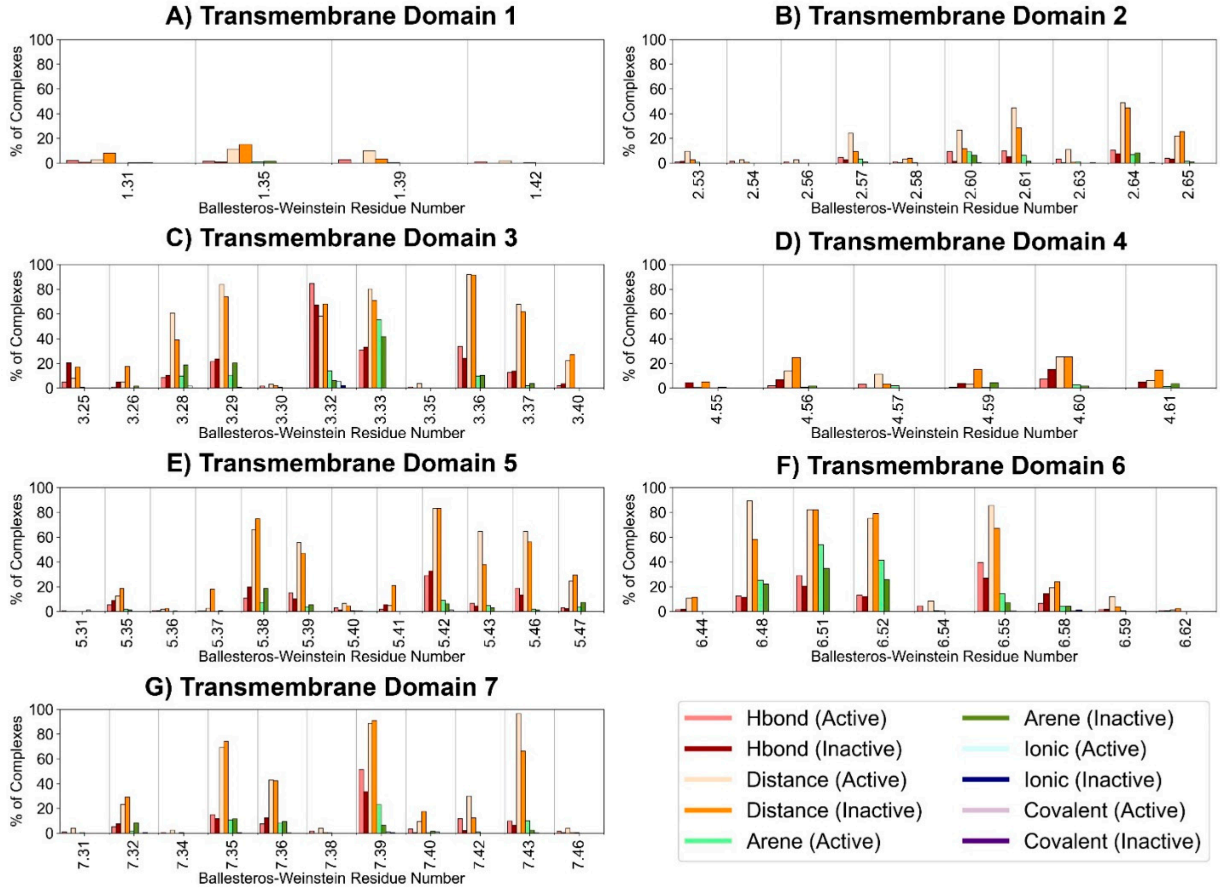

Supplemental Figure S8. Interaction percentages (by interaction type) for Ballesteros-Weinstein indexed residue positions in each transmembrane domain for residue positions possessing interactions in ≥ 10 complexes in the initial dataset.
