# Supplementary material for: G Protein-Coupled Receptor–Ligand Pose and Functional Class Prediction
Source: Int J Mol Sci. 2024 Jun 22;25(13):6876. doi: 10.3390/ijms25136876 (PMC11241240; doi:10.3390/ijms25136876)
Supplement: Supplementary file 1 [file ijms-25-06876-s001.zip › Szwabowski_IJMS_2024_Supplemental_TablesS1_to_S5_References.pdf]

GPCR Ligand Pose and Functional Class Prediction

Gregory L. Szwabowski [1], Makenzie Griffing [2], Elijah J. Mugabe [3], Daniel O'Malley[4], Lindsey N. Baker[5], Daniel L. Baker [6,*], and Abby L. Parrill [7,*]

Department of Chemistry, University of Memphis, Memphis TN 38152, USA

1 gszwabowski@gmail.com
2 makenziegriffing@outlook.com
3 elm4026@med.cornell.edu
4 domalley@memphis.edu
5 lindsey.baker@yale.edu
6 dlbaker@memphis.edu
7 aparrill@memphis.edu
* Correspondence: aparrill@memphis.edu; Tel.: 1-901-678-3371 (AP) or dlbaker@memphis.edu (DB)

SUPPLEMENTAL TABLES

Table S1. List of experimentally determined Class A GPCR structures used in fingerprint development.					
PDBID	GPCR	Species	Publication Date	Activation State	Ligand Name
5V54[1]	5HT1B	Homo sapiens	2018-02-07	Inactive	CHEMBL428892
4IAQ[2]	5HT1B	Homo sapiens	2013-03-13	Inactive	dihydroergotamine
6G79[3]	5HT1B	Homo sapiens	2018-06-20	Active	donitriptan
4IAR[2]	5HT1B	Homo sapiens	2013-03-13	Inactive	ergotamine
6A93[4]	5HT2A	Homo sapiens	2019-02-13	Inactive	risperidone
6A94[4]	5HT2A	Homo sapiens	2019-02-13	Inactive	zotepine
6DS0[5]	5HT2B	Homo sapiens	2018-08-29	Intermediate	LY266097
5TVN[6]	5HT2B	Homo sapiens	2017-02-01	Intermediate	lysergide
4IB4[7]	5HT2B	Homo sapiens	2013-03-13	Intermediate	ergotamine
4NC3[8]	5HT2B	Homo sapiens	2013-12-18	Intermediate	ergotamine
5TUD[9]	5HT2B	Homo sapiens	2017-07-26	Active	ergotamine
6DRX[5]	5HT2B	Homo sapiens	2018-08-29	Intermediate	lisuride
6DRY[5]	5HT2B	Homo sapiens	2018-08-29	Intermediate	methylergonovine
6DRZ[5]	5HT2B	Homo sapiens	2018-08-29	Intermediate	methysergide
6BQG[10]	5HT2C	Homo sapiens	2018-02-14	Active	ergotamine
6BQH[10]	5HT2C	Homo sapiens	2018-02-14	Inactive	ritanserin
5UEN[11]	AA1R	Homo sapiens	2017-03-01	Inactive	DU172
5N2S[12]	AA1R	Homo sapiens	2017-07-26	Inactive	PSB36
6D9H[13]	AA1R	Homo sapiens	2018-06-20	Active	adenosine
5IU7[14]	AA2AR	Homo sapiens	2016-06-29	Inactive	2-(furan-2-yl)-5-N-[2-(4-phenylpiperidin-1-yl)ethyl]-1H-[1,2,4]triazolo[1,5-a][1,3,5]triazin-8-ium-5,7-diamine
5IUA[14]	AA2AR	Homo sapiens	2016-06-29	Inactive	2-(furan-2-yl)-5-N-[3-(4-phenylpiperazin-1-yl)propyl]-1H-[1,2,4]triazolo[1,5-a][1,3,5]triazin-8-ium-5,7-diamine
5UIG[15]	AA2AR	Homo sapiens	2017-02-08	Inactive	5-amino-N-[(2-methoxyphenyl)methyl]-2-(3-methylphenyl)-2h-1,2,3-triazole-4-carboximidamide
4UG2[16]	AA2AR	Homo sapiens	2015-04-08	Active	CGS 21680
4UHR[16]	AA2AR	Homo sapiens	2015-04-08	Active	CGS 21680
5OLV[17]	AA2AR	Homo sapiens	2018-01-17	Inactive	CHEMBL1671936
5IUB[14]	AA2AR	Homo sapiens	2016-06-29	Inactive	CHEMBL184061
3UZC[18]	AA2AR	Homo sapiens	2012-03-21	Inactive	CHEMBL2024114
5OLZ[17]	AA2AR	Homo sapiens	2018-01-17	Inactive	CHEMBL2024114
5OM1[17]	AA2AR	Homo sapiens	2018-01-17	Inactive	CHEMBL2024114
5OM4[17]	AA2AR	Homo sapiens	2018-01-17	Inactive	CHEMBL2024114
5IU8[14]	AA2AR	Homo sapiens	2016-06-29	Inactive	CHEMBL3934661
2YDV[19]	AA2AR	Homo sapiens	2011-05-18	Active	NECA
5G53[20]	AA2AR	Homo sapiens	2016-08-03	Active	NECA
6GDG[21]	AA2AR	Homo sapiens	2018-05-16	Active	NECA

5N2R[12]	AA2AR	Homo sapiens	2017-07-26	Inactive	PSB36
3QAK[22]	AA2AR	Homo sapiens	2011-03-09	Active	UK-432,097
5WF5[23]	AA2AR	Homo sapiens	2018-02-21	Active	UK-432,097
5WF6[23]	AA2AR	Homo sapiens	2018-02-21	Active	UK-432,097
3EML[24]	AA2AR	Homo sapiens	2008-10-14	Inactive	ZM-241385
3PWH[25]	AA2AR	Homo sapiens	2011-09-07	Inactive	ZM-241385
3VG9[26]	AA2AR	Homo sapiens	2012-02-01	Inactive	ZM-241385
3VGA[26]	AA2AR	Homo sapiens	2012-02-01	Inactive	ZM-241385
4EIY[27]	AA2AR	Homo sapiens	2012-07-25	Inactive	ZM-241385
5IU4[14]	AA2AR	Homo sapiens	2016-06-29	Inactive	ZM-241385
5JTB[28]	AA2AR	Homo sapiens	2017-05-31	Inactive	ZM-241385
5K2A[29]	AA2AR	Homo sapiens	2016-09-21	Inactive	ZM-241385
5K2B[29]	AA2AR	Homo sapiens	2016-09-21	Inactive	ZM-241385
5K2C[29]	AA2AR	Homo sapiens	2016-09-21	Inactive	ZM-241385
5K2D[29]	AA2AR	Homo sapiens	2016-09-21	Inactive	ZM-241385
5NLX[30]	AA2AR	Homo sapiens	2017-09-27	Inactive	ZM-241385
5NM2[30]	AA2AR	Homo sapiens	2017-09-27	Inactive	ZM-241385
5NM4[30]	AA2AR	Homo sapiens	2017-09-27	Inactive	ZM-241385
5OLG[17]	AA2AR	Homo sapiens	2018-01-17	Inactive	ZM-241385
5UVI[31]	AA2AR	Homo sapiens	2017-05-24	Inactive	ZM-241385
5VRA[32]	AA2AR	Homo sapiens	2017-12-13	Inactive	ZM-241385
6AQF[33]	AA2AR	Homo sapiens	2018-01-10	Inactive	ZM-241385
2YDO[19]	AA2AR	Homo sapiens	2011-05-18	Active	adenosine
3RFM[25]	AA2AR	Homo sapiens	2011-09-07	Inactive	caffeine
5MZP[12]	AA2AR	Homo sapiens	2017-07-26	Inactive	caffeine
3UZA[18]	AA2AR	Homo sapiens	2012-03-21	Inactive	compound 4g [PMID: 22220592]
5MZJ[12]	AA2AR	Homo sapiens	2017-07-26	Inactive	theophylline
5OLO[17]	AA2AR	Homo sapiens	2018-01-17	Inactive	tozadenant
5OLH[17]	AA2AR	Homo sapiens	2018-01-17	Inactive	vipadenant
3REY[25]	AA2AR	Homo sapiens	2011-09-07	Inactive	xanthine amine congener
5CXV[34]	ACM1	Homo sapiens	2016-03-09	Inactive	0HK
6OIJ[35]	ACM1	Homo sapiens	2019-05-08	Active	iperoxo
5ZKB[36]	ACM2	Homo sapiens	2018-11-21	Inactive	AF-DX 384
3UON[37]	ACM2	Homo sapiens	2012-02-01	Inactive	CHEMBL558910
5ZK3[36]	ACM2	Homo sapiens	2018-11-21	Inactive	CHEMBL558910
5YC8[36]	ACM2	Homo sapiens	2018-11-21	Inactive	N-methyl scopolamine
5ZK8[36]	ACM2	Homo sapiens	2018-11-21	Inactive	N-methyl scopolamine
5ZKC[36]	ACM2	Homo sapiens	2018-11-21	Inactive	N-methyl scopolamine
4MQS[38]	ACM2	Homo sapiens	2013-11-27	Active	iperoxo
5ZHP[39]	ACM3	Rattus norvegicus	2018-11-28	Inactive	(1R,2R,4S,5S,7s)-7-({[4-fluoro-2-(thiophen-2-yl)phenyl]carbamoyl}oxy)-9,9-dimethyl-3-oxa-9-azatricyclo[3.3.1.0~2,4~]nonan-9-ium
4U16[40]	ACM3	Rattus norvegicus	2014-11-26	Inactive	N-methyl scopolamine
4DAJ[41]	ACM3	Rattus norvegicus	2012-02-22	Inactive	tiotropium

4U14[40]	ACM3	Rattus norvegicus	2014-11-26	Inactive	tiotropium
4U15[40]	ACM3	Rattus norvegicus	2014-11-26	Inactive	tiotropium
5DSG[34]	ACM4	Homo sapiens	2016-03-16	Inactive	tiotropium
6OL9[42]	ACM5	Homo sapiens	2019-12-11	Inactive	tiotropium
2VT4[43]	ADRB1	Meleagris gallopavo	2008-06-24	Inactive	(S)-cyanopindolol
2YCY[44]	ADRB1	Meleagris gallopavo	2011-06-08	Inactive	(S)-cyanopindolol
4BVN[45]	ADRB1	Meleagris gallopavo	2014-04-02	Inactive	(S)-cyanopindolol
5F8U[46]	ADRB1	Meleagris gallopavo	2015-12-23	Inactive	(S)-cyanopindolol
6H7O[47]	ADRB1	Meleagris gallopavo	2018-10-17	Active	(S)-cyanopindolol
6H7N[47]	ADRB1	Meleagris gallopavo	2018-10-17	Active	(S)-xamoterol
5A8E[48]	ADRB1	Meleagris gallopavo	2015-09-30	Inactive	7-methylcyanopindolol
4AMI[49]	ADRB1	Meleagris gallopavo	2012-05-23	Inactive	bucindolol
2Y02[50]	ADRB1	Meleagris gallopavo	2011-01-12	Inactive	carmoterol
3ZPR[51]	ADRB1	Meleagris gallopavo	2013-04-03	Inactive	CHEMBL1559535
3ZPQ[51]	ADRB1	Meleagris gallopavo	2013-04-03	Inactive	CHEMBL200234
4AMJ[49]	ADRB1	Meleagris gallopavo	2012-05-23	Inactive	CHEMBL3799125
2YCW[44]	ADRB1	Meleagris gallopavo	2011-06-01	Inactive	carazolol
2Y00[50]	ADRB1	Meleagris gallopavo	2011-01-12	Inactive	dobutamine
2Y01[50]	ADRB1	Meleagris gallopavo	2011-03-30	Inactive	dobutamine
6H7L[47]	ADRB1	Meleagris gallopavo	2018-10-17	Active	dobutamine
2YCZ[44]	ADRB1	Meleagris gallopavo	2011-06-01	Inactive	iodocyanopindolol
2Y03[50]	ADRB1	Meleagris gallopavo	2011-01-12	Inactive	levisoprenaline
6H7J[47]	ADRB1	Meleagris gallopavo	2018-10-17	Active	levisoprenaline
6IBL[52]	ADRB1	Meleagris gallopavo	2019-01-09	Active	arformoterol
2Y04[50]	ADRB1	Meleagris gallopavo	2011-01-12	Inactive	levosalbutamol

6H7M[47]	ADRB1	Meleagris gallopavo	2018-10-17	Active	levosalbutamol
3P0G[53]	ADRB2	Homo sapiens	2011-01-19	Active	BI-167107
3SN6[54]	ADRB2	Homo sapiens	2011-07-20	Active	BI-167107
4LDE[55]	ADRB2	Homo sapiens	2013-09-25	Active	BI-167107
3NYA[56]	ADRB2	Homo sapiens	2010-08-11	Inactive	CHEMBL1160734
3NY8[56]	ADRB2	Homo sapiens	2010-08-11	Inactive	CHEMBL1233766
3NY9[56]	ADRB2	Homo sapiens	2010-08-11	Inactive	CHEMBL1233771
2RH1[57]	ADRB2	Homo sapiens	2007-10-30	Inactive	carazolol
4GBR[58]	ADRB2	Homo sapiens	2012-10-24	Inactive	carazolol
5D5A[59]	ADRB2	Homo sapiens	2016-01-13	Inactive	carazolol
5D5B[59]	ADRB2	Homo sapiens	2016-01-13	Inactive	carazolol
5D6L[60]	ADRB2	Homo sapiens	2016-08-17	Inactive	carazolol
5JQH[61]	ADRB2	Homo sapiens	2016-07-13	Inactive	carazolol
3PDS[62]	ADRB2	Homo sapiens	2011-01-12	Inactive	FAUC50
4QKX[63]	ADRB2	Homo sapiens	2014-07-23	Active	Q27453560
6MXT[64]	ADRB2	Homo sapiens	2018-11-14	Active	salmeterol
3D4S[65]	ADRB2	Homo sapiens	2008-06-17	Inactive	timolol
4LDL[55]	ADRB2	Homo sapiens	2013-09-25	Active	hydroxybenzylisoproterenol
4LDO[55]	ADRB2	Homo sapiens	2013-09-25	Active	(-)-adrenaline
5X7D[66]	ADRB2	Homo sapiens	2017-08-16	Inactive	carazolol
4ZUD[67]	AGTR1	Homo sapiens	2015-10-07	Inactive	olmesartan
4YAY[68]	AGTR1	Homo sapiens	2015-04-22	Inactive	ZD-7155
6DO1[69]	AGTR1	Homo sapiens	2019-01-30	Active	angiotensin-like peptide S1I8
5UNF[70]	AGTR2	Homo sapiens	2017-04-05	Active	compound 1 [PMID: 28379944]
5UNG[70]	AGTR2	Homo sapiens	2017-04-05	Active	compound 1 [PMID: 28379944]
5UNH[70]	AGTR2	Homo sapiens	2017-04-05	Active	compound 2 [PMID: 28379944]
6JOD[71]	AGTR2	Homo sapiens	2020-01-15	Active	angiotensin II
5XJM[72]	AGTR2	Homo sapiens	2018-07-11	Active	ile8-angiotensin II
6KNM[73]	APJ	Homo sapiens	2020-01-29	Inactive	JN241
5VBL [74]	APJ	Homo sapiens	2017-05-31	Inactive	AMG3054
5O9H[75]	C5AR1	Homo sapiens	2018-01-10	Inactive	NDT9513727
6C1Q [76]	C5AR1	Homo sapiens	2018-05-30	Inactive	NDT9513727
6C1R [76]	C5AR1	Homo sapiens	2018-05-30	Inactive	PMX53
6GPS[77]	CCR2	Homo sapiens	2019-01-02	Inactive	MK-0812
6GPX[77]	CCR2	Homo sapiens	2019-01-02	Inactive	MK-0812
5T1A [78]	CCR2	Homo sapiens	2016-12-14	Inactive	(3S)-1-{(1S,2R,4R)-4-[methyl(propan-2-yl)amino]-2-propylcyclohexyl}-3-{[6-(trifluoromethyl)quinazolin-4-yl]amino}pyrrolidin-2-one
6AKY[79]	CCR5	Homo sapiens	2018-10-24	Inactive	CCR5 antagonist 34
6AKX[79]	CCR5	Homo sapiens	2018-10-24	Inactive	N-[(1S)-3-{(3-exo)-3-[3-methyl-5-(propan-2-yl)-4H-1,2,4-triazol-4-yl]-8-azabicyclo[3.2.1]octan-8-yl}-1-(thiophen-2-yl)propyl]cyclopentanecarboxamide

4MBS[80]	CCR5	Homo sapiens	2013-09-11	Inactive	maraviroc
6MEO [81]	CCR5	Homo sapiens	2018-12-12	Inactive	GP120
6MET [81]	CCR5	Homo sapiens	2018-12-12	Inactive	GP120
5UIW [82]	CCR5	Homo sapiens	2017-06-28	Inactive	5P7-CCL5
6QZH [83]	CCR7	Homo sapiens	2019-09-04	Inactive	3-[[4-[[(1~{R})-2,2-dimethyl-1-(5-methylfuran-2-yl)propyl]amino]-1,1-bis(oxidanylidene)-1,2,5-thiadiazol-3-yl]amino]-~{N},~{N},6-trimethyl-2-oxidanyl-benzamide
5LWE [84]	CCR9	Homo sapiens	2016-12-07	Inactive	vercirnon
6RZ4[85]	CLTR1	Homo sapiens	2019-10-30	Intermediate	pranlukast
6RZ5[85]	CLTR1	Homo sapiens	2019-10-30	Intermediate	zafirlukast
6RZ8[85]	CLTR2	Homo sapiens	2019-12-11	Intermediate	ONO-2080365
6RZ6[85]	CLTR2	Homo sapiens	2019-12-11	Intermediate	ONO-2570366
6RZ7[85]	CLTR2	Homo sapiens	2019-12-11	Intermediate	ONO-2570366
6RZ9[85]	CLTR2	Homo sapiens	2019-12-11	Intermediate	ONO-2770372
5XRA[86]	CNR1	Homo sapiens	2017-07-12	Active	AM11542
5TGZ[87]	CNR1	Homo sapiens	2016-11-02	Inactive	AM6538
5XR8[86]	CNR1	Homo sapiens	2017-07-12	Active	AM841
6N4B[88]	CNR1	Homo sapiens	2019-01-30	Active	MDMB-fubinaca
5U09[89]	CNR1	Homo sapiens	2016-12-07	Inactive	taranabant
5ZTY[90]	CNR2	Homo sapiens	2019-01-30	Inactive	AM10257
6KPC[91]	CNR2	Homo sapiens	2020-02-12	Inactive	AM841
6KPF[91]	CNR2	Homo sapiens	2020-02-12	Active	AM841
6PT0[92]	CNR2	Homo sapiens	2020-02-12	Active	WIN55212-2
3ODU[93]	CXCR4	Homo sapiens	2010-10-27	Inactive	isothiourea-1t
3OE6[93]	CXCR4	Homo sapiens	2010-10-27	Inactive	isothiourea-1t
3OE8[93]	CXCR4	Homo sapiens	2010-10-27	Inactive	isothiourea-1t
3OE9[93]	CXCR4	Homo sapiens	2010-10-27	Inactive	isothiourea-1t
3OE0[93]	CXCR4	Homo sapiens	2010-10-27	Inactive	CVX15
4RWS [94]	CXCR4	Homo sapiens	2015-02-11	Inactive	vMIP-II
6LUQ[95]	DRD2	Homo sapiens	2020-03-04	Inactive	haloperidol
6CM4[96]	DRD2	Homo sapiens	2018-03-14	Inactive	risperidone
3PBL[97]	DRD3	Homo sapiens	2010-11-03	Inactive	eticlopride
5WIU[98]	DRD4	Homo sapiens	2017-10-18	Inactive	nemonapride
5WIV[98]	DRD4	Homo sapiens	2017-10-18	Inactive	nemonapride
6K1Q[99]	EDNRB	Homo sapiens	2019-07-17	Inactive	IRL 2500
5X93[100]	EDNRB	Homo sapiens	2017-08-16	Inactive	K-8794
5GLH [101]	EDNRB	Homo sapiens	2016-09-07	Inactive	endothelin
6IGK [102]	EDNRB	Homo sapiens	2018-11-21	Inactive	endothelin-3
6IGL [102]	EDNRB	Homo sapiens	2018-11-21	Inactive	IRL1620
6K1Q [103]	EDNRB	Homo sapiens	2019-07-17	Inactive	IRL2500
6LRY [104]	EDNRB	Homo sapiens	2020-02-12	Inactive	sarafotoxin S6b
5TZR[105]	FFAR1	Homo sapiens	2017-06-07	Intermediate	MK-8666
5TZY[105]	FFAR1	Homo sapiens	2017-06-07	Intermediate	MK-8666

5KW2 [106]	FFAR1	Homo sapiens	2018-05-02	Intermediate	(3~{S})-3-cyclopropyl-3-[2-[1-[2-[2,2-dimethylpropyl-(6-methylpyridin-2-yl)carbamoyl]-5-methoxy-phenyl]piperidin-4-yl]-1-benzofuran-6-yl]propanoic acid
4PHU[107]	FFAR1	Homo sapiens	2014-07-16	Intermediate	fasiglifam
6OMM[108]	FPR2	Homo sapiens	2020-02-26	Active	CHEMBL552527
6LW5[109]	FPR2	Homo sapiens	2020-03-25	Active	CHEMBL552527
6LI0[110]	GPR52	Homo sapiens	2020-02-26	Inactive	derivative 17
3RZE[111]	HRH1	Homo sapiens	2011-06-15	Inactive	doxepin
4Z36[112]	LPAR1	Homo sapiens	2015-06-03	Inactive	ONO-3080573
4Z34[112]	LPAR1	Homo sapiens	2015-06-03	Inactive	ONO-9780307
4Z35[112]	LPAR1	Homo sapiens	2015-06-03	Inactive	ONO-9910539
6ME4[113]	MTR1A	Homo sapiens	2019-04-24	Inactive	2-iodomelatonin
6ME3[113]	MTR1A	Homo sapiens	2019-04-24	Inactive	2-phenylmelatonin
6PS8[114]	MTR1A	Homo sapiens	2019-11-13	Inactive	2-phenylmelatonin
6ME5[113]	MTR1A	Homo sapiens	2019-04-24	Inactive	agomelatine
6ME2[113]	MTR1A	Homo sapiens	2019-04-24	Inactive	ramelteon
6ME6[113]	MTR1B	Homo sapiens	2019-04-24	Inactive	2-phenylmelatonin
6ME7[113]	MTR1B	Homo sapiens	2019-04-24	Inactive	2-phenylmelatonin
6ME8[113]	MTR1B	Homo sapiens	2019-04-24	Inactive	2-phenylmelatonin
6ME9[113]	MTR1B	Homo sapiens	2019-04-24	Inactive	ramelteon
6HLL[115]	NK1R	Homo sapiens	2019-01-16	Inactive	CP 99994
6E59[116]	NK1R	Homo sapiens	2018-12-12	Inactive	L760735
6HLO[115]	NK1R	Homo sapiens	2019-01-16	Inactive	aprepitant
6J20[117]	NK1R	Homo sapiens	2019-03-06	Inactive	aprepitant
6J21[117]	NK1R	Homo sapiens	2019-03-06	Inactive	aprepitant
6HLP[115]	NK1R	Homo sapiens	2019-01-16	Inactive	netupitant
5ZBH[118]	NPY1R	Homo sapiens	2018-04-25	Inactive	BMS-193885
5ZBQ[118]	NPY1R	Homo sapiens	2018-04-25	Inactive	CHEMBL3747822
3ZEV [119]	NTR1	Rattus norvegicus	2014-01-29	Inactive	neurotensin
4BUO [119]	NTR1	Rattus norvegicus	2014-01-29	Inactive	neurotensin
4BV0 [119]	NTR1	Rattus norvegicus	2014-01-29	Inactive	neurotensin
4BWB [119]	NTR1	Rattus norvegicus	2014-01-29	Inactive	neurotensin
4GRV [120]	NTR1	Rattus norvegicus	2012-10-17	Active	neurotensin 8-13
4XEE [121]	NTR1	Rattus norvegicus	2015-07-29	Active	neurotensin
4XES [121]	NTR1	Rattus norvegicus	2015-07-29	Active	neurotensin
5T04 [122]	NTR1	Rattus norvegicus	2016-12-21	Active	neurotensin
4EJ4[123]	OPRD	Mus musculus	2012-05-16	Inactive	naltrindole
4N6H[124]	OPRD	Homo sapiens	2013-12-25	Inactive	naltrindole
4RWD [125]	OPRD	Homo sapiens	2015-01-14	Inactive	(3S)-2-[(2S)-2-azanyl-3-(2,6-dimethyl-4-oxidanyl-phenyl)propanoyl]-N-[(2S)-1-[[(2S)-1-azanyl-1-oxidanylidene-3-phenyl-propan-2-yl]amino]-1-oxidanylidene-3-phenyl-

					propan-2-yl]-3,4-dihydro-1H-isoquinoline-3-carboxamide
4RWA [125]	OPRD	Homo sapiens	2015-01-14	Inactive	(3S)-2-[(2S)-2-azanyl-3-(2,6-dimethyl-4-oxidanyl-phenyl)propanoyl]-N-[(2S)-1-[[(2S)-1-azanyl-1-oxidanylidene-3-phenyl-propan-2-yl]amino]-1-oxidanylidene-3-phenyl-propan-2-yl]-3,4-dihydro-1H-isoquinoline-3-carboxamide
4DJH[126]	OPRK	Homo sapiens	2012-03-21	Inactive	JDTic
6B73[127]	OPRK	Homo sapiens	2018-01-17	Active	MP1104
5C1M[128]	OPRM	Mus musculus	2015-08-05	Active	BU72
4DKL[129]	OPRM	Mus musculus	2012-03-21	Inactive	methyl 4-[[(4R,4aS,7R,7aR,12bS)-3-(cyclopropylmethyl)-4a,9-dihydroxy-1,2,4,5,6,7,7a,13-octahydro-4,12-methanobenzofuro[3,2-e]isoquinolin-7-yl]amino]-4-oxobutanoate
6DDE [130]	OPRM	Mus musculus	2018-06-13	Active	DAMGO
6DDF [130]	OPRM	Mus musculus	2018-06-13	Active	DAMGO
5DHG[131]	OPRX	Homo sapiens	2015-10-21	Inactive	CHEMBL1783826
5DHH[131]	OPRX	Homo sapiens	2015-10-21	Inactive	SB 612111
4EA3[132]	OPRX	Homo sapiens	2012-04-25	Inactive	compound 24 [PMID: 16451050]
2ZIY [133]	OPSD	Todarodes pacificus	2008-05-06	Inactive	11-cis retinal
6TP4[134]	OX1R	Homo sapiens	2020-01-01	Inactive	ACT-462206
6TOS[134]	OX1R	Homo sapiens	2020-01-15	Inactive	CHEMBL2413522
6TQ4[134]	OX1R	Homo sapiens	2020-01-01	Inactive	CHEMBL4590386
6TQ6[134]	OX1R	Homo sapiens	2020-01-01	Inactive	CHEMBL4592653
6TQ7[134]	OX1R	Homo sapiens	2020-01-01	Inactive	SB-334867
6TQ9[134]	OX1R	Homo sapiens	2020-01-01	Inactive	SB-408124
4ZJC[135]	OX1R	Homo sapiens	2016-03-09	Inactive	SB-674042
6TP3[134]	OX1R	Homo sapiens	2020-01-01	Inactive	daridorexant
6TP6[134]	OX1R	Homo sapiens	2020-01-01	Inactive	filorexant
6TOT[134]	OX1R	Homo sapiens	2020-01-15	Inactive	lemborexant
4ZJ8[135]	OX1R	Homo sapiens	2016-03-09	Inactive	suvorexant
6TO7[134]	OX1R	Homo sapiens	2020-01-01	Inactive	suvorexant
5WQC[136]	OX2R	Homo sapiens	2017-11-29	Inactive	EMPA
5WS3[136]	OX2R	Homo sapiens	2017-12-13	Inactive	EMPA
6TPG[134]	OX2R	Homo sapiens	2020-01-01	Inactive	EMPA
6TPN[134]	OX2R	Homo sapiens	2020-01-01	Inactive	HTL6641
4S0V[137]	OX2R	Homo sapiens	2015-01-14	Inactive	suvorexant
6TPJ[134]	OX2R	Homo sapiens	2020-01-01	Inactive	suvorexant
5NDZ [138]	PAR2	Homo sapiens	2017-05-03	Intermediate	2-(6-bromanyl-1,3-benzodioxol-5-yl)-~{N}-(4-cyanophenyl)-1-[(1~{S})-1-cyclohexylethyl]benzimidazole-5-carboxamide

4XNW[139]	P2RY1	Homo sapiens	2015-04-01	Intermediate	MRS2500
4XNV[139]	P2RY1	Homo sapiens	2015-04-01	Intermediate	1-[2-(2-tert-butylphenoxy)pyridin-3-yl]-3-[4-(trifluoromethoxy)phenyl]urea
4PXZ[140]	P2Y12	Homo sapiens	2014-04-30	Intermediate	2-(methylsulfanyl)adenosine 5'-(trihydrogen diphosphate)
4PY0[140]	P2Y12	Homo sapiens	2014-04-30	Intermediate	2-(methylsulfanyl)adenosine 5'-(trihydrogen diphosphate)
4NTJ[141]	P2Y12	Homo sapiens	2014-03-26	Intermediate	AZD1283
3VW7[142]	PAR1	Homo sapiens	2012-12-12	Intermediate	vorapaxar
5NDD[143]	PAR2	Homo sapiens	2017-05-03	Intermediate	AZ8838
6D27[144]	PD2R2	Homo sapiens	2018-10-03	Inactive	CHEMBL179036
6D26[144]	PD2R2	Homo sapiens	2018-10-03	Inactive	fevipiprant
6M9T[145]	PE2R3	Homo sapiens	2018-12-05	Active	misoprostol-FA
6AK3[146]	PE2R3	Homo sapiens	2018-12-05	Active	prostaglandin E_2
5YWY[147]	PE2R4	Homo sapiens	2018-12-05	Inactive	ONO-AE3-208
5YHL[147]	PE2R4	Homo sapiens	2018-12-05	Inactive	ONO-AE3-208-Br
5ZKQ[148]	PTAFR	Homo sapiens	2018-06-20	Intermediate	ABT-491
5ZKP[148]	PTAFR	Homo sapiens	2018-06-20	Other	foropafant
5X33[149]	Q9WTK1	Cavia porcellus	2018-01-03	Inactive	BIIL 260
3V2W[150]	S1PR1	Homo sapiens	2012-02-15	Inactive	W146
3V2Y[150]	S1PR1	Homo sapiens	2012-02-15	Inactive	W146
6RNK[151]	SUCR1	Rattus norvegicus	2019-08-14	Intermediate	NF-56-EJ40
6IIV[152]	TA2R	Homo sapiens	2018-12-19	Intermediate	daltroban
6IIU[152]	TA2R	Homo sapiens	2018-12-19	Intermediate	ramatroban
4XT3 [153]	US28	Strain AD169	2015-03-04	Active	CX_3CL1
4XT1 [153]	US28	Strain AD169	2015-03-04	Active	CX_3CL1
5WB2 [154]	US38	Strain AD169	2018-06-13	Active	CX_3CL1

Table S2. Cross-docking structure pairs used to test interaction fingerprints. Ligands docked in two different charge states due to ionizable groups with pKa values near 7 are noted with *.

Receptor	Protein PDB	Protein State	Ligand PDB	Ligand Function	Fingerprint(s)
AA1R	5N2S [12]	Inactive	6D9H [13]	Agonist	A, B
AA1R	6D9H [13]	Inactive	5N2S [12]	Agonist	A, B
AA1R	5N2S [12]	Inactive	5UEN [11]	Antagonist	Inactive
AA1R	5UEN [11]	Inactive	5N2S [12]	Agonist	Inactive
AA2AR	5WF5 [23]	Active	4UHR [16]	Agonist	A, B
AA2AR	4UHR [16]	Active	5WF5 [23]	Agonist	A, B
AA2AR	5WF5 [23]	Active	4UG2 [16]	Agonist	Active
AA2AR	4UG2 [16]	Active	5WF5* [23]	Agonist	Active
AA2AR	5IU4 [14]	Inactive	5MJZ [12]	Antagonist	Inactive
AA2AR	5MZJ [12]	Inactive	5IU4 [14]	Antagonist	Inactive
ADRB1	6IBL [52]	Active	4BVN [45]	Antagonist	A, B
ADRB1	4BVN [45]	Inactive	6IBL [52]	Agonist	A, B
ADRB1	6IBL [52]	Active	7JJO [155]	Agonist	Active
ADRB1	7JJO [155]	Active	6IBL [52]	Agonist	Active
ADRB1	4BVN [45]	Inactive	4AMJ [49]	Inverse Agonist	Inactive
ADRB1	4AMJ [49]	Inactive	4BVN [45]	Antagonist	Inactive
ADRB2	4LDE [55]	Active	5X7D [66]	Antagonist	A, B
ADRB2	5X7D [66]	Inactive	4LDE [55]	Agonist	A, B
ADRB2	4LDE [55]	Active	6MXT [64]	Agonist	Active
ADRB2	6MXT [64]	Active	4LDE [55]	Agonist	Active
CLTR1	6RZ5 [85]	Intermediate	6RZ4 [85]	Antagonist	Intermediate
CLTR1	6RZ4 [85]	Intermediate	6RZ5 [85]	Antagonist	Intermediate
CLTR1	6RZ7 [156]	Intermediate	6RZ8 [156]	Antagonist	Intermediate
CLTR1	6RZ8 [156]	Intermediate	6RZ7 [156]	Antagonist	Intermediate
CNR1	5XRA [86]	Active	5TGZ [87]	Antagonist	A, B
CNR1	5TGZ [87]	Inactive	5XRA [86]	Agonist	A, B
CNR1	5XRA [86]	Active	5U09 [89]	Inverse Agonist	A, B
CNR1	5U09 [89]	Inactive	5XRA [86]	Agonist	A, B
CNR1	5XRA [86]	Active	6N4B [88]	Agonist	Active
CNR1	6N4B [88]	Active	5XRA [86]	Agonist	Active
CNR1	5U09 [89]	Inactive	5TGZ [87]	Antagonist	A, B, Inactive
CNR1	5TGZ [87]	Inactive	5U09 [89]	Inverse Agonist	A, B, Inactive
MTR1A	6PS8 [114]	Inactive	6ME5 [113]	Agonist	Inactive
MTR1A	6ME5 [113]	Inactive	6PS8 [114]	Agonist	Inactive
MTR1B	6ME8 [113]	Inactive	6ME9 [113]	Agonist	Inactive
MTR1B	6ME9 [113]	Inactive	6ME8 [113]	Agonist	Inactive
OPRK	4DJH [126]	Inactive	6B73 [127]	Agonist	A, B
OPRK	6B73 [127]	Active	4DJH [126]	Antagonist	A, B
P2Y12	4PXZ [127]	Intermediate	4NTJ [141]	Antagonist	Intermediate
P2Y12	4NTJ [141]	Intermediate	4PXZ [140]	Agonist	Intermediate
5HT1B	4IAR [2]	Inactive	6G79 [3]	Agonist	A, B
5HT1B	6G79 [3]	Active	4IAR [2]	Agonist	A, B
5HT1B	4IAR [2]	Inactive	5V54 [1]	Antagonist	Inactive
5HT1B	5V54 [1]	Inactive	4IAR [2]	Agonist	Inactive
5HT2A	6A93 [4]	Inactive	6A94 [4]	Antagonist	Inactive
5HT2A	6A94 [4]	Inactive	6A93 [4]	Antagonist	Inactive
5HT2B	4IB4 [7]	Intermediate	6DRZ [5]	Antagonist	Intermediate
5HT2B	6DRZ [5]	Intermediate	4IB4 [7]	Agonist	Intermediate
5HT2B	6DRX [5]	Intermediate	6DS0 [5]	Antagonist	Intermediate

5HT2B	6DS0 [5]	Intermediate	6DRX [5]	Antagonist	Intermediate
5HT2B	6DRY [5]	Intermediate	5TVN [6]	Agonist	Intermediate
5HT2B	5TVN [6]	Intermediate	6DRY [5]	Agonist	Intermediate
5HT2B	6DRY [5]	Intermediate	6DS0 [5]	Antagonist	Intermediate
5HT2B	6DS0 [5]	Intermediate	6DRY [5]	Agonist	Intermediate
5HT2B	6DRX [5]	Intermediate	5TVN [6]	Agonist	Intermediate
5HT2B	5TVN [6]	Intermediate	6DRX [5]	Antagonist	Intermediate
5HT2C	6BQG [10]	Active	6BQH [10]	Inverse Agonist	A, B
5HT2C	6BQH [10]	Inactive	6BQG [10]	Agonist	A, B

Table S3. Homology modeling targets and templates for GPCR comprising the external dataset used to validate the classifier.

Target Receptor	Template Receptor	CoINPocket Score[c]	Template PDBID	Template State	Loop Anchor 1[a]	Loop Anchor 2
GPR31	SUCNR	1.19	6RNK	Intermediate	S153	G171
TAAR2	5HT1B	2.14	5V54	Inactive	F180	K207

[a]Anchor residues used for extracellular loop 2 modeling are numbered to match the numbering scheme present on GPCRdb.[157]

Table S4. List of experimentally determined Class A GPCR structures used in ligand prediction.

PDBID	GPCR	Species	Publication Date	Activation State	Ligand Name	Ligand Activity	Cross-docking PDBID[a]
7E2Y[158]	5HT1A	Homo sapiens	2021-04-14	Active	5-hydroxytryptamine	Agonist	7E2X
7E2Z[158]	5HT1A	Homo sapiens	2021-04-14	Active	aripiprazole	Agonist (partial)	7E2Y
5V54[1]	5HT1B	Homo sapiens	2018-02-07	Inactive	CHEMBL428892	Antagonist	7C61
4IAQ[2]	5HT1B	Homo sapiens	2013-03-13	Inactive	dihydroergotamine	Agonist	4IAR
6G79[3]	5HT1B	Homo sapiens	2018-06-20	Active	donitriptan	Agonist	4IAR
4IAR[2]	5HT1B	Homo sapiens	2013-03-13	Inactive	ergotamine	Agonist	4IAQ
7C61[159]	5HT1B	Homo sapiens	2020-07-29	Inactive	ergotamine	Antagonist	5V54
7E32[158]	5HT1D	Homo sapiens	2021-04-21	Active	5-hydroxytryptamine	Agonist	NA[a]
7E33[158]	5HT1E	Homo sapiens	2021-04-14	Active	BRL-54443	Agonist	NA
7EXD[160]	5HT1F	Homo sapiens	2021-08-04	Active	lasmiditan	Agonist	NA
6WHA[161]	5HT2A	Homo sapiens	2020-09-23	Active	25-CN-NBOH	Agonist	7WC8
6WH4[161]	5HT2A	Homo sapiens	2020-09-23	Inactive	CHEMBL428892	Inverse agonist	7WC8
6WGT[161]	5HT2A	Homo sapiens	2020-09-23	Inactive	lysergide	Agonist	7WC8
7VOE[162]	5HT2A	Homo sapiens	2021-12-22	Inactive	aripiprazole	Agonist (partial)	7VOD
7VOD[162]	5HT2A	Homo sapiens	2021-12-22	Inactive	cariprazine	Agonist (partial)	7VOE
6A93[4]	5HT2A	Homo sapiens	2019-02-13	Inactive	risperidone	Antagonist	6A94
6A94[4]	5HT2A	Homo sapiens	2019-02-13	Inactive	zotepine	Antagonist	6A93
6DS0[5]	5HT2B	Homo sapiens	2018-08-29	Intermediate	LY266097	Antagonist	6DRX
5TVN[6]	5HT2B	Homo sapiens	2017-02-01	Intermediate	lysergide	Agonist	4IB4
4IB4[7]	5HT2B	Homo sapiens	2013-03-13	Intermediate	ergotamine	Agonist	4NC3
4NC3[8]	5HT2B	Homo sapiens	2013-12-18	Intermediate	ergotamine	Agonist	4IB4
5TUD[9]	5HT2B	Homo sapiens	2017-07-26	Active	ergotamine	Agonist	4IB4
6DRX[5]	5HT2B	Homo sapiens	2018-08-29	Intermediate	lisuride	Antagonist	6DRZ
6DRY[5]	5HT2B	Homo sapiens	2018-08-29	Intermediate	methylergonovine	Agonist	4IB4
6DRZ[5]	5HT2B	Homo sapiens	2018-08-29	Intermediate	methysergide	Antagonist	6DRX
6BQG[10]	5HT2C	Homo sapiens	2018-02-14	Active	ergotamine	Agonist	6BQH
6BQH[10]	5HT2C	Homo sapiens	2018-02-14	Inactive	ritanserin	Inverse agonist	6BQG
5UEN[11]	AA1R	Homo sapiens	2017-03-01	Inactive	DU172	Antagonist	5N2S

5N2S[12]	AA1R	Homo sapiens	2017-07-26	Inactive	PSB36	Agonist	5UEN
6D9H[13]	AA1R	Homo sapiens	2018-06-20	Active	adenosine	Agonist	7LD3
7LD3[163]	AA1R	Homo sapiens	2021-09-08	Active	adenosine	Agonist	7LD4
7LD4[163]	AA1R	Homo sapiens	2021-09-08	Active	adenosine	Agonist	7LD3
5IU7[14]	AA2AR	Homo sapiens	2016-06-29	Inactive	2-(furan-2-yl)-5-N-[2-(4-phenylpiperidin-1-yl)ethyl]-1H-[1,2,4]triazolo[1,5-a][1,3,5]triazin-8-ium-5,7-diamine	Antagonist	5NM4
5IUA[14]	AA2AR	Homo sapiens	2016-06-29	Inactive	2-(furan-2-yl)-5-N-[3-(4-phenylpiperazin-1-yl)propyl]-1H-[1,2,4]triazolo[1,5-a][1,3,5]triazin-8-ium-5,7-diamine	Antagonist	5NM4
6ZDV[164]	AA2AR	Homo sapiens	2020-09-16	Inactive	2-methyl-3-(4-methylthiazol-2-yl)-4-oxo-6-propyl-4H-chromen-7-yl acetate	Antagonist	5NM4
5UIG[15]	AA2AR	Homo sapiens	2017-02-08	Inactive	5-amino-N-[(2-methoxyphenyl)methyl]-2-(3-methylphenyl)-2h-1,2,3-triazole-4-carboximidamide	Antagonist	5NM4
4UG2[16]	AA2AR	Homo sapiens	2015-04-08	Active	CGS 21680	Agonist	5WF5
4UHR[16]	AA2AR	Homo sapiens	2015-04-08	Active	CGS 21680	Agonist	5WF5
7ARO[165]	AA2AR	Homo sapiens	2021-04-07	Inactive	CHEMBL124345	Agonist (partial)	5NM4
5OLV[17]	AA2AR	Homo sapiens	2018-01-17	Inactive	CHEMBL1671936	Antagonist	5NM4
5IUB[14]	AA2AR	Homo sapiens	2016-06-29	Inactive	CHEMBL184061	Antagonist	5NM4
3UZC[18]	AA2AR	Homo sapiens	2012-03-21	Inactive	CHEMBL2024114	Antagonist	5NM4
5OLZ[17]	AA2AR	Homo sapiens	2018-01-17	Inactive	CHEMBL2024114	Antagonist	5NM4
5OM1[17]	AA2AR	Homo sapiens	2018-01-17	Inactive	CHEMBL2024114	Antagonist	5NM4
5OM4[17]	AA2AR	Homo sapiens	2018-01-17	Inactive	CHEMBL2024114	Antagonist	5NM4
6ZDR[164]	AA2AR	Homo sapiens	2020-09-16	Inactive	CHEMBL2030687	Antagonist	5NM4
5IU8[14]	AA2AR	Homo sapiens	2016-06-29	Inactive	CHEMBL3934661	Antagonist	5NM4
2YDV[19]	AA2AR	Homo sapiens	2011-05-18	Active	NECA	Agonist	5WF5
5G53[20]	AA2AR	Homo sapiens	2016-08-03	Active	NECA	Agonist	5WF5
6GDG[21]	AA2AR	Homo sapiens	2018-05-16	Active	NECA	Agonist	5WF5
5N2R[12]	AA2AR	Homo sapiens	2017-07-26	Inactive	PSB36	Antagonist	5NM4
3QAK[22]	AA2AR	Homo sapiens	2011-03-09	Active	UK-432,097	Agonist	5WF5
5WF5[23]	AA2AR	Homo sapiens	2018-02-21	Active	UK-432,097	Agonist	4UHR
5WF6[23]	AA2AR	Homo sapiens	2018-02-21	Active	UK-432,097	Agonist	5WF5
3EML[24]	AA2AR	Homo sapiens	2008-10-14	Inactive	ZM-241385	Antagonist	5NM4

3PWH[25]	AA2AR	Homo sapiens	2011-09-07	Inactive	ZM-241385	Antagonist	5NM4
3VG9[26]	AA2AR	Homo sapiens	2012-02-01	Inactive	ZM-241385	Antagonist	5NM4
3VGA[26]	AA2AR	Homo sapiens	2012-02-01	Inactive	ZM-241385	Antagonist	5NM4
4EIY[27]	AA2AR	Homo sapiens	2012-07-25	Inactive	ZM-241385	Antagonist	5NM4
5IU4[14]	AA2AR	Homo sapiens	2016-06-29	Inactive	ZM-241385	Antagonist	5NM4
5JTB[28]	AA2AR	Homo sapiens	2017-05-31	Inactive	ZM-241385	Antagonist	5NM4
5K2A[29]	AA2AR	Homo sapiens	2016-09-21	Inactive	ZM-241385	Antagonist	5NM4
5K2B[29]	AA2AR	Homo sapiens	2016-09-21	Inactive	ZM-241385	Antagonist	5NM4
5K2C[29]	AA2AR	Homo sapiens	2016-09-21	Inactive	ZM-241385	Antagonist	5NM4
5K2D[29]	AA2AR	Homo sapiens	2016-09-21	Inactive	ZM-241385	Antagonist	5NM4
5NLX[30]	AA2AR	Homo sapiens	2017-09-27	Inactive	ZM-241385	Antagonist	5NM4
5NM2[30]	AA2AR	Homo sapiens	2017-09-27	Inactive	ZM-241385	Antagonist	5NM4
5NM4[30]	AA2AR	Homo sapiens	2017-09-27	Inactive	ZM-241385	Antagonist	5IU4
5OLG[17]	AA2AR	Homo sapiens	2018-01-17	Inactive	ZM-241385	Antagonist	5NM4
5UVI[31]	AA2AR	Homo sapiens	2017-05-24	Inactive	ZM-241385	Antagonist	5NM4
5VRA[32]	AA2AR	Homo sapiens	2017-12-13	Inactive	ZM-241385	Antagonist	5NM4
6AQF[33]	AA2AR	Homo sapiens	2018-01-10	Inactive	ZM-241385	Antagonist	5NM4
6JZH[166]	AA2AR	Homo sapiens	2019-10-30	Inactive	ZM-241385	Antagonist	5NM4
6LPJ[167]	AA2AR	Homo sapiens	2020-11-25	Inactive	ZM-241385	Antagonist	5NM4
6LPK[167]	AA2AR	Homo sapiens	2020-11-25	Inactive	ZM-241385	Antagonist	5NM4
6LPL[167]	AA2AR	Homo sapiens	2020-11-25	Inactive	ZM-241385	Antagonist	5NM4
6MH8[168]	AA2AR	Homo sapiens	2019-04-24	Inactive	ZM-241385	Antagonist	5NM4
6PS7[114]	AA2AR	Homo sapiens	2019-11-13	Inactive	ZM-241385	Antagonist	5NM4
6S0L[169]	AA2AR	Homo sapiens	2020-07-15	Inactive	ZM-241385	Antagonist	5NM4
6S0Q[169]	AA2AR	Homo sapiens	2020-07-15	Inactive	ZM-241385	Antagonist	5NM4
6WQA[170]	AA2AR	Homo sapiens	2020-11-18	Inactive	ZM-241385	Antagonist	5NM4
7RM5[171]	AA2AR	Homo sapiens	2021-09-08	Inactive	ZM-241385	Antagonist	5NM4
2YDO[19]	AA2AR	Homo sapiens	2011-05-18	Active	adenosine	Agonist	5WF5
3RFM[25]	AA2AR	Homo sapiens	2011-09-07	Inactive	caffeine	Antagonist	5NM4
5MZP[12]	AA2AR	Homo sapiens	2017-07-26	Inactive	caffeine	Antagonist	5NM4
3UZA[18]	AA2AR	Homo sapiens	2012-03-21	Inactive	compound 4g [PMID: 22220592]	Antagonist	5NM4
6GT3[172]	AA2AR	Homo sapiens	2019-06-26	Inactive	imaradenant	Antagonist	5NM4
5MZJ[12]	AA2AR	Homo sapiens	2017-07-26	Inactive	theophylline	Antagonist	5NM4
5OLO[17]	AA2AR	Homo sapiens	2018-01-17	Inactive	tozadenant	Antagonist	5NM4

5OLH[17]	AA2AR	Homo sapiens	2018-01-17	Inactive	vipadenant	Antagonist	5NM4
3REY[25]	AA2AR	Homo sapiens	2011-09-07	Inactive	xanthine amine congener	Antagonist	5NM4
5CXV[34]	ACM1	Homo sapiens	2016-03-09	Inactive	0HK	Antagonist	6WJC
6ZG9[173]	ACM1	Homo sapiens	2021-10-06	Inactive	7-fluoro-5-methyl-3-[1-(oxan-4-yl)piperidin-4-yl]-1H-benzimidazol-2-one	Agonist	6ZFZ
6ZFZ[173]	ACM1	Homo sapiens	2021-10-06	Inactive	CHEMBL3354065	Agonist	6ZG4
6ZG4[173]	ACM1	Homo sapiens	2021-10-06	Inactive	ethyl (4S)-4-[4-[(1-methylcyclobutyl)carbamoyl]piperidin-1-yl]azepane-1-carboxylate	Agonist	6ZFZ
6OIJ[35]	ACM1	Homo sapiens	2019-05-08	Active	iperoxo	Agonist	6ZFZ
5ZKB[36]	ACM2	Homo sapiens	2018-11-21	Inactive	AF-DX 384	Antagonist	5ZKC
3UON[37]	ACM2	Homo sapiens	2012-02-01	Inactive	CHEMBL558910	Antagonist	5ZKC
5ZK3[36]	ACM2	Homo sapiens	2018-11-21	Inactive	CHEMBL558910	Antagonist	5ZKC
6U1N[174]	ACM2	Homo sapiens	2020-02-26	Active	LY2119620	Agonist	4MQS
5YC8[36]	ACM2	Homo sapiens	2018-11-21	Inactive	N-methyl scopolamine	Antagonist	5ZKC
5ZK8[36]	ACM2	Homo sapiens	2018-11-21	Inactive	N-methyl scopolamine	Antagonist	5ZKC
5ZKC[36]	ACM2	Homo sapiens	2018-11-21	Inactive	N-methyl scopolamine	Antagonist	5YC8
4MQS[38]	ACM2	Homo sapiens	2013-11-27	Active	iperoxo	Agonist	6OIK
5ZHP[39]	ACM3	Rattus norvegicus	2018-11-28	Inactive	(1R,2R,4S,5S,7s)-7-({[4-fluoro-2-(thiophen-2-yl)phenyl]carbamoyl}oxy)-9,9-dimethyl-3-oxa-9-azatricyclo[3.3.1.0~2,4~]nonan-9-ium	Antagonist	4U15
4U16[40]	ACM3	Rattus norvegicus	2014-11-26	Inactive	N-methyl scopolamine	Antagonist	4U15
4DAJ[41]	ACM3	Rattus norvegicus	2012-02-22	Inactive	tiotropium	Antagonist	4U15
4U14[40]	ACM3	Rattus norvegicus	2014-11-26	Inactive	tiotropium	Antagonist	4U15
5DSG[34]	ACM4	Homo sapiens	2016-03-16	Inactive	tiotropium	Antagonist	6KP6
6OL9[42]	ACM5	Homo sapiens	2019-12-11	Inactive	tiotropium	Inverse agonist	NA
6KUY[175]	ADRA2R	Homo sapiens	2019-12-04	Inactive	(2~{S})-4-fluoranyl-2-(1~{H}-imidazol-5-yl)-1-propan-2-yl-2,3-dihydroindole	Agonist (partial)	6KUX
6KUX[175]	ADRA2R	Homo sapiens	2019-12-04	Inactive	RSC	Antagonist	6KUY
6K41[176]	ADA2B	Homo sapiens	2020-04-15	Active	dexmedetomidine	Agonist	6K42
6K42[176]	ADA2B	Homo sapiens	2020-04-15	Active	dexmedetomidine	Agonist	6K41

6KUW[177]	ADA2C	Homo sapiens	2019-12-04	Inactive	(8aR,12aS,13aR)-12-ethylsulfonyl-3-methoxy-5,6,8,8a,9,10,11,12a,13,13a-decahydroisoquinolino[2,1-g][1,6]naphthyridine	Antagonist	NA
7BTS[178]	ADRB1	Homo sapiens	2020-12-02	Active	(-)-adrenaline	Agonist	6H7N
2VT4[43]	ADRB1	Meleagris gallopavo	2008-06-24	Inactive	(S)-cyanopindolol	Antagonist	4BVN
2YCY[44]	ADRB1	Meleagris gallopavo	2011-06-08	Inactive	(S)-cyanopindolol	Antagonist	4BVN
4BVN[45]	ADRB1	Meleagris gallopavo	2014-04-02	Inactive	(S)-cyanopindolol	Antagonist	3ZPR
5F8U[46]	ADRB1	Meleagris gallopavo	2015-12-23	Inactive	(S)-cyanopindolol	Antagonist	4BVN
6H7O[47]	ADRB1	Meleagris gallopavo	2018-10-17	Active	(S)-cyanopindolol	Agonist	6H7N
6H7N[47]	ADRB1	Meleagris gallopavo	2018-10-17	Active	(S)-xamoterol	Agonist	7BU7
5A8E[48]	ADRB1	Meleagris gallopavo	2015-09-30	Inactive	7-methylcyanopindolol	Inverse agonist	4AMJ
7BU7[178]	ADRB1	Homo sapiens	2020-12-02	Active	BI-167107	Agonist	6H7N
4AMI[49]	ADRB1	Meleagris gallopavo	2012-05-23	Inactive	bucindolol	Agonist	2Y02
2Y02[50]	ADRB1	Meleagris gallopavo	2011-01-12	Inactive	carmoterol	Agonist	2Y03
3ZPR[51]	ADRB1	Meleagris gallopavo	2013-04-03	Inactive	CHEMBL1559535	Antagonist	4BVN
3ZPQ[51]	ADRB1	Meleagris gallopavo	2013-04-03	Inactive	CHEMBL200234	Antagonist	4BVN
4AMJ[49]	ADRB1	Meleagris gallopavo	2012-05-23	Inactive	CHEMBL3799125	Inverse agonist	5A8E
2YCW[44]	ADRB1	Meleagris gallopavo	2011-06-01	Inactive	carazolol	Antagonist	4BVN
7BVQ[178]	ADRB1	Homo sapiens	2020-12-02	Inactive	carazolol	Inverse agonist	4AMJ
2Y00[50]	ADRB1	Meleagris gallopavo	2011-01-12	Inactive	dobutamine	Agonist (partial)	2Y01

2Y01[50]	ADRB1	Meleagris gallopavo	2011-03-30	Inactive	dobutamine	Agonist (partial)	2Y00
6H7L[47]	ADRB1	Meleagris gallopavo	2018-10-17	Active	dobutamine	Agonist	6H7N
2YCZ[44]	ADRB1	Meleagris gallopavo	2011-06-01	Inactive	iodocyanopindolol	Antagonist	4BVN
2Y03[50]	ADRB1	Meleagris gallopavo	2011-01-12	Inactive	levisoprenaline	Agonist	2Y02
6H7J[47]	ADRB1	Meleagris gallopavo	2018-10-17	Active	levisoprenaline	Agonist	6H7N
7JJO[155]	ADRB1	Meleagris gallopavo	2020-09-02	Active	levisoprenaline	Agonist	6H7N
7BU6[178]	ADRB1	Homo sapiens	2020-12-02	Active	noradrenaline	Agonist	6H7N
6IBL[52]	ADRB1	Meleagris gallopavo	2019-01-09	Active	arformoterol	Agonist	6H7N
6TKO[52]	ADRB1	Meleagris gallopavo	2020-06-17	Active	arformoterol	Agonist	6H7N
2Y04[50]	ADRB1	Meleagris gallopavo	2011-01-12	Inactive	levosalbutamol	Agonist (partial)	2Y00
6H7M[47]	ADRB1	Meleagris gallopavo	2018-10-17	Active	levosalbutamol	Agonist	6H7N
3P0G[53]	ADRB2	Homo sapiens	2011-01-19	Active	BI-167107	Agonist	4LDE
3SN6[54]	ADRB2	Homo sapiens	2011-07-20	Active	BI-167107	Agonist	4LDE
4LDE[55]	ADRB2	Homo sapiens	2013-09-25	Active	BI-167107	Agonist	6MXT
6E67[179]	ADRB2	Homo sapiens	2019-06-05	Active	BI-167107	Agonist	4LDE
6NI3[180]	ADRB2	Homo sapiens	2019-11-20	Active	BI-167107	Agonist	4LDE
3NYA[56]	ADRB2	Homo sapiens	2010-08-11	Inactive	CHEMBL1160734	Antagonist	6PS2
6PRZ[114]	ADRB2	Homo sapiens	2019-11-13	Inactive	CHEMBL1160734	Antagonist	6PS2
6PS2[114]	ADRB2	Homo sapiens	2019-11-13	Inactive	CHEMBL1160734	Antagonist	6PS3
3NY8[56]	ADRB2	Homo sapiens	2010-08-11	Inactive	CHEMBL1233766	Inverse agonist	2RH1
6PS4[114]	ADRB2	Homo sapiens	2019-11-13	Inactive	CHEMBL1233766	Antagonist	6PS2
3NY9[56]	ADRB2	Homo sapiens	2010-08-11	Inactive	CHEMBL1233771	Inverse agonist	2RH1
6PS3[114]	ADRB2	Homo sapiens	2019-11-13	Inactive	CHEMBL3799125	Antagonist	6PS2
2RH1[57]	ADRB2	Homo sapiens	2007-10-30	Inactive	carazolol	Inverse agonist	5D5A

4GBR[58]	ADRB2	Homo sapiens	2012-10-24	Inactive	carazolol	Inverse agonist	2RH1
5D5A[59]	ADRB2	Homo sapiens	2016-01-13	Inactive	carazolol	Inverse agonist	2RH1
5D5B[59]	ADRB2	Homo sapiens	2016-01-13	Inactive	carazolol	Inverse agonist	2RH1
5D6L[60]	ADRB2	Homo sapiens	2016-08-17	Inactive	carazolol	Inverse agonist	2RH1
5JQH[61]	ADRB2	Homo sapiens	2016-07-13	Inactive	carazolol	Inverse agonist	2RH1
3PDS[62]	ADRB2	Homo sapiens	2011-01-12	Inactive	FAUC50	Agonist	6PS2
7DHR[181]	ADRB2	Homo sapiens	2020-12-16	Active	levisoprenaline	Agonist	4LDE
4QKX[63]	ADRB2	Homo sapiens	2014-07-23	Active	Q27453560	Agonist	4LDE
7BZ2[182]	ADRB2	Homo sapiens	2020-08-05	Active	arformoterol	Agonist	4LDE
6PS0[114]	ADRB2	Homo sapiens	2019-11-13	Inactive	carazolol	Antagonist	6PS2
7DHI[181]	ADRB2	Homo sapiens	2020-12-16	Active	levosalbutamol	Agonist	4LDE
6PS5[114]	ADRB2	Homo sapiens	2019-11-13	Inactive	propranolol	Antagonist	6PS2
6MXT[64]	ADRB2	Homo sapiens	2018-11-14	Active	salmeterol	Agonist	4LDE
3D4S[65]	ADRB2	Homo sapiens	2008-06-17	Inactive	timolol	Inverse agonist	2RH1
6PS1[114]	ADRB2	Homo sapiens	2019-11-13	Inactive	timolol	Antagonist	6PS2
6PS6[114]	ADRB2	Homo sapiens	2019-11-13	Inactive	timolol	Antagonist	6PS2
7DH5[183]	ADRB3	Canis lupus familiaris	2021-08-04	Active	mirabegron	Agonist	NA
4ZUD[67]	AGTR1	Homo sapiens	2015-10-07	Inactive	olmesartan	Inverse agonist	4YAY
4YAY[68]	AGTR1	Homo sapiens	2015-04-22	Inactive	ZD-7155	Antagonist	4ZUD
5UNF[70]	AGTR2	Homo sapiens	2017-04-05	Active	compound 1 [PMID: 28379944]	Antagonist	5UNG
5UNG[70]	AGTR2	Homo sapiens	2017-04-05	Active	compound 1 [PMID: 28379944]	Antagonist	5UNF
5UNH[70]	AGTR2	Homo sapiens	2017-04-05	Active	compound 2 [PMID: 28379944]	Antagonist	5UNF
5O9H[75]	C5AR1	Homo sapiens	2018-01-10	Inactive	NDT9513727	Antagonist	6C1R
7F8Y[184]	CCKAR	Homo sapiens	2021-10-13	Inactive	devazepide	Antagonist	7F8U
7F8U[184]	CCKAR	Homo sapiens	2021-10-13	Inactive	lintitript	Antagonist	7F8Y
6GPS[77]	CCR2	Homo sapiens	2019-01-02	Inactive	MK-0812	Antagonist	6GPX
6GPX[77]	CCR2	Homo sapiens	2019-01-02	Inactive	MK-0812	Antagonist	6GPS
6AKY[79]	CCR5	Homo sapiens	2018-10-24	Inactive	CCR5 antagonist 34	Antagonist	5UIW
6AKX[79]	CCR5	Homo sapiens	2018-10-24	Inactive	N-[(1S)-3-{(3-exo)-3-[3-methyl-5-(propan-2-yl)-4H-1,2,4-triazol-4-yl]-8-azabicyclo[3.2.1]octan-8-yl}-1-(thiophen-2-yl)propyl]cyclopentanecarboxamide	Antagonist	5UIW
4MBS[80]	CCR5	Homo sapiens	2013-09-11	Inactive	maraviroc	Antagonist	5UIW

6RZ4[85]	CLTR1	Homo sapiens	2019-10-30	Intermediate	pranlukast	Antagonist	6RZ5
6RZ5[85]	CLTR1	Homo sapiens	2019-10-30	Intermediate	zafirlukast	Antagonist	6RZ4
6RZ8 [156]	CLTR2	Homo sapiens	2019-12-11	Intermediate	ONO-2080365	Antagonist	6RZ6
6RZ6[85]	CLTR2	Homo sapiens	2019-12-11	Intermediate	ONO-2570366	Antagonist	6RZ7
6RZ7 [156]	CLTR2	Homo sapiens	2019-12-11	Intermediate	ONO-2570366	Antagonist	6RZ6
6RZ9 [156]	CLTR2	Homo sapiens	2019-12-11	Intermediate	ONO-2770372	Antagonist	6RZ6
5XRA[86]	CNR1	Homo sapiens	2017-07-12	Active	AM11542	Agonist	5XR8
5TGZ[87]	CNR1	Homo sapiens	2016-11-02	Inactive	AM6538	Antagonist	5U09
5XR8[86]	CNR1	Homo sapiens	2017-07-12	Active	AM841	Agonist	5XRA
6KPG[91]	CNR1	Homo sapiens	2020-02-12	Active	AM841	Agonist	5XRA
7V3Z[185]	CNR1	Homo sapiens	2021-11-24	Active	CP55940	Agonist	5XRA
6N4B[88]	CNR1	Homo sapiens	2019-01-30	Active	MDMB-fubinaca	Agonist	5XRA
5U09[89]	CNR1	Homo sapiens	2016-12-07	Inactive	taranabant	Inverse agonist	5TGZ
5ZTY[90]	CNR2	Homo sapiens	2019-01-30	Inactive	AM10257	Antagonist	6KPC
6KPC[91]	CNR2	Homo sapiens	2020-02-12	Inactive	AM841	Agonist	5ZTY
6KPF[91]	CNR2	Homo sapiens	2020-02-12	Active	AM841	Agonist	6PT0
6PT0[92]	CNR2	Homo sapiens	2020-02-12	Active	WIN55212-2	Agonist	6KPF
3ODU[93]	CXCR4	Homo sapiens	2010-10-27	Inactive	isothiourea-1t	Antagonist	3OE0
3OE6[93]	CXCR4	Homo sapiens	2010-10-27	Inactive	isothiourea-1t	Antagonist	3ODU
3OE8[93]	CXCR4	Homo sapiens	2010-10-27	Inactive	isothiourea-1t	Antagonist	3ODU
3OE9[93]	CXCR4	Homo sapiens	2010-10-27	Inactive	isothiourea-1t	Antagonist	3ODU
7JVP[186]	DRD1	Homo sapiens	2021-02-24	Active	39YLC3L0ZU	Agonist	7JVQ
7CKX[187]	DRD1	Homo sapiens	2021-03-03	Active	A77636	Agonist	7JVP
7JV5[186]	DRD1	Homo sapiens	2021-02-24	Active	CHEMBL1160787	Agonist	7JVP
7CRH[187]	DRD1	Homo sapiens	2021-03-03	Active	CHEMBL1416789	Agonist	7JVP
7CKY[187]	DRD1	Homo sapiens	2021-03-03	Active	CHEMBL3697578	Agonist	7JVP
7JOZ[188]	DRD1	Homo sapiens	2021-04-14	Active	CHEMBL4453318	Agonist	7JVP
7JVQ[186]	DRD1	Homo sapiens	2021-02-24	Active	apomorphine	Agonist	7JVP
7CKW[187]	DRD1	Homo sapiens	2021-03-03	Active	fenoldopam	Agonist	7JVP
6VMS[189]	DRD2	Homo sapiens	2020-06-17	Active	bromocriptine	Agonist	7JVR
7JVR[186]	DRD2	Homo sapiens	2021-02-24	Active	bromocriptine	Agonist	6VMS
6LUQ[95]	DRD2	Homo sapiens	2020-03-04	Inactive	haloperidol	Antagonist	7DFP
6CM4[96]	DRD2	Homo sapiens	2018-03-14	Inactive	risperidone	Inverse agonist	6LUQ
7DFP[190]	DRD2	Homo sapiens	2020-12-30	Inactive	spiperone	Antagonist	6LUQ

7CMV[191]	DRD3	Homo sapiens	2021-03-10	Active	CHEMBL70565	Agonist	7CMU
3PBL[97]	DRD3	Homo sapiens	2010-11-03	Inactive	eticlopride	Antagonist	7CMV
7CMU[191]	DRD3	Homo sapiens	2021-03-10	Active	pramipexole	Agonist	7CMV
6IQL[192]	DRD4	Mus musculus	2019-12-04	Inactive	L745870	Antagonist	5WIU
5WIU[98]	DRD4	Homo sapiens	2017-10-18	Inactive	nemonapride	Antagonist	5WIV
5WIV[98]	DRD4	Homo sapiens	2017-10-18	Inactive	nemonapride	Antagonist	5WIU
6K1Q[99]	EDNRB	Homo sapiens	2019-07-17	Inactive	IRL 2500	Inverse agonist	6IGK
5X93[100]	EDNRB	Homo sapiens	2017-08-16	Inactive	K-8794	Antagonist	6IGK
5TZR[105]	FFAR1	Homo sapiens	2017-06-07	Intermediate	MK-8666	Agonist	4PHU
4PHU[107]	FFAR1	Homo sapiens	2014-07-16	Intermediate	fasiglifam	Agonist	5TZR
6KO5[193]	GHSR	Homo sapiens	2020-08-12	Inactive	CHEMBL1956994	Antagonist	7F83
7NA8[194]	GHSR	Homo sapiens	2021-12-15	Active	ibutamoren	Agonist	7NA7
7BR3[195]	GNRHR	Homo sapiens	2020-10-07	Inactive	elagolix	Antagonist	NA
7VUG[196]	GP139	Homo sapiens	2021-12-29	Active	JNJ-63533054	Agonist	7VUH
7VUH[196]	GP139	Homo sapiens	2021-12-29	Active	JNJ-63533054	Agonist	7VUG
7VUI[196]	GP139	Homo sapiens	2021-12-29	Active	JNJ-63533054	Agonist	7VUG
7VUJ[196]	GP139	Homo sapiens	2021-12-29	Active	JNJ-63533054	Agonist	7VUG
7CFM[197]	GPBAR	Homo sapiens	2020-09-09	Active	CHEMBL2331646	Agonist	7CFN
6LI0[110]	GPR52	Homo sapiens	2020-02-26	Inactive	derivative 17	Agonist	6LI2
3RZE[111]	HRH1	Homo sapiens	2011-06-15	Inactive	doxepin	Antagonist	7DFL
7DFL[198]	HRH1	Homo sapiens	2021-03-31	Active	histamine	Agonist	3RZE
4Z36[112]	LPAR1	Homo sapiens	2015-06-03	Inactive	ONO-3080573	Antagonist	4Z35
4Z34[112]	LPAR1	Homo sapiens	2015-06-03	Inactive	ONO-9780307	Antagonist	4Z35
4Z35[112]	LPAR1	Homo sapiens	2015-06-03	Inactive	ONO-9910539	Antagonist	4Z36
7K15[199]	LT4R1	Homo sapiens	2021-02-17	Inactive	N-(tert-butylsulfonyl)-4-fluoro-2-{(3S,4R)-4-hydroxy-3-[(pyridin-2-yl)methyl]-3,4-dihydro-2H-1-benzopyran-7-yl}benzamide	Antagonist	7VKT
7F58[200]	MC4R	Homo sapiens	2021-11-03	Active	(3R)-N-[(2R)-3-(4-chlorophenyl)-1-[4-cyclohexyl-4-(1,2,4-triazol-1-ylmethyl) piperidin-1-yl]-1-oxidanylidene-propan-2-yl]-1,2,3,4-tetrahydroisoquinoline-3-carboxamide	Agonist	7PIU
7S8N[201]	MRGX2	Homo sapiens	2021-11-17	Active	(R)-ZINC-3573	Agonist	7S8L
7S8O[201]	MRGX2	Homo sapiens	2021-11-17	Active	(R)-ZINC-3573	Agonist	7S8L
7VDH[201]	MRGX2	Homo sapiens	2021-12-01	Active	compound 48/80	Agonist	7S8L

7VV6[201]	MRGX2	Homo sapiens	2021-12-01	Active	compound 48/80	Agonist	7S8L
7S8P[201]	MRGX4	Homo sapiens	2021-11-17	Active	N-[(1R,3R,5S,7R)-3,5-dimethyltricyclo[3.3.1.1~3,7~]decane-1-carbonyl]-D-phenylalanine	Agonist	NA
6ME4[113]	MTR1A	Homo sapiens	2019-04-24	Inactive	2-iodomelatonin	Agonist	6ME2
6ME3[113]	MTR1A	Homo sapiens	2019-04-24	Inactive	2-phenylmelatonin	Agonist	6ME2
6PS8[114]	MTR1A	Homo sapiens	2019-11-13	Inactive	2-phenylmelatonin	Agonist	6ME2
6ME5[113]	MTR1A	Homo sapiens	2019-04-24	Inactive	agomelatine	Agonist	6ME2
6ME2[113]	MTR1A	Homo sapiens	2019-04-24	Inactive	ramelteon	Agonist	6ME3
7DB6[202]	MTR1A	Homo sapiens	2021-08-18	Active	ramelteon	Agonist	7VGY
6ME6[113]	MTR1B	Homo sapiens	2019-04-24	Inactive	2-phenylmelatonin	Agonist	6ME8
6ME7[113]	MTR1B	Homo sapiens	2019-04-24	Inactive	2-phenylmelatonin	Agonist	6ME6
6ME8[113]	MTR1B	Homo sapiens	2019-04-24	Inactive	2-phenylmelatonin	Agonist	6ME6
6ME9[113]	MTR1B	Homo sapiens	2019-04-24	Inactive	ramelteon	Agonist	6ME6
6HLL[115]	NK1R	Homo sapiens	2019-01-16	Inactive	CP 99994	Antagonist	6HLP
6E59[116]	NK1R	Homo sapiens	2018-12-12	Inactive	L760735	Antagonist	6HLP
6HLO[115]	NK1R	Homo sapiens	2019-01-16	Inactive	aprepitant	Antagonist	6HLP
6J20[117]	NK1R	Homo sapiens	2019-03-06	Inactive	aprepitant	Antagonist	6HLP
6J21[117]	NK1R	Homo sapiens	2019-03-06	Inactive	aprepitant	Antagonist	6HLP
6HLP[115]	NK1R	Homo sapiens	2019-01-16	Inactive	netupitant	Antagonist	6HLO
5ZBH[118]	NPY1R	Homo sapiens	2018-04-25	Inactive	BMS-193885	Antagonist	5ZBQ
5ZBQ[118]	NPY1R	Homo sapiens	2018-04-25	Inactive	CHEMBL3747822	Antagonist	5ZBH
7DDZ[203]	NPY2R	Homo sapiens	2021-01-27	Inactive	CHEMBL1823578	Antagonist	NA
6Z8N[204]	NTR1	Rattus norvegicus	2021-02-10	Intermediate	(2~{S})-4-methyl-2-[(1-quinolin-8-ylsulfonylindol-3-yl)carbonylamino] pentanoic acid	Agonist	6Z4V
6ZA8[204]	NTR1	Rattus norvegicus	2021-02-10	Intermediate	CHEMBL508044	Agonist (partial)	6Z4V
6Z4Q[204]	NTR1	Rattus norvegicus	2021-02-10	Inactive	SR142948A	Inverse agonist	6ZIN
6Z4S[204]	NTR1	Rattus norvegicus	2021-02-10	Inactive	meclinertant	Inverse agonist	6ZIN
6ZIN[204]	NTR1	Rattus norvegicus	2021-02-10	Inactive	meclinertant	Inverse agonist	6Z4S
6PT3[205]	OPRD	Homo sapiens	2019-12-11	Active	DPI-287	Agonist	6PT2

4EJ4[123]	OPRD	Mus musculus	2012-05-16	Inactive	naltrindole	Antagonist	4N6H
4N6H[124]	OPRD	Homo sapiens	2013-12-25	Inactive	naltrindole	Antagonist	4RWD
4DJH[126]	OPRK	Homo sapiens	2012-03-21	Inactive	JDTic	Antagonist	6VI4
6VI4[206]	OPRK	Homo sapiens	2020-03-18	Inactive	JDTic	Antagonist	4DJH
6B73[127]	OPRK	Homo sapiens	2018-01-17	Active	MP1104	Agonist	4DJH
5C1M[128]	OPRM	Mus musculus	2015-08-05	Active	BU72	Agonist	7SBF
4DKL[129]	OPRM	Mus musculus	2012-03-21	Inactive	methyl 4-[[(4R,4aS,7R,7aR,12bS)-3-(cyclopropylmethyl)-4a,9-dihydroxy-1,2,4,5,6,7,7a,13-octahydro-4,12-methanobenzofuro[3,2-e]isoquinolin-7-yl]amino]-4-oxobutanoate	Antagonist	5C1M
5DHG[131]	OPRX	Homo sapiens	2015-10-21	Inactive	CHEMBL1783826	Antagonist	5DHH
5DHH[131]	OPRX	Homo sapiens	2015-10-21	Inactive	SB 612111	Antagonist	5DHG
4EA3[132]	OPRX	Homo sapiens	2012-04-25	Inactive	compound 24 [PMID: 16451050]	Antagonist	5DHG
6TP4[134]	OX1R	Homo sapiens	2020-01-01	Inactive	ACT-462206	Antagonist	6TOD
6TOS[134]	OX1R	Homo sapiens	2020-01-15	Inactive	CHEMBL2413522	Antagonist	6TOD
6TQ4[134]	OX1R	Homo sapiens	2020-01-01	Inactive	CHEMBL4590386	Antagonist	6TOD
6TQ6[134]	OX1R	Homo sapiens	2020-01-01	Inactive	CHEMBL4592653	Antagonist	6TOD
6TOD[134]	OX1R	Homo sapiens	2020-01-01	Inactive	EMPA: N-ethyl-2-[(6-methoxypyridin-3-yl)-(2-methylphenyl)sulfonyl-amino]-N-(pyridin-3-ylmethyl)ethanamide	Antagonist	6TOS
6V9S[207]	OX1R	Homo sapiens	2020-07-15	Inactive	JH112	Antagonist	6TOD
6TQ7[134]	OX1R	Homo sapiens	2020-01-01	Inactive	SB-334867	Antagonist	6TOD
6TQ9[134]	OX1R	Homo sapiens	2020-01-01	Inactive	SB-408124	Antagonist	6TOD
4ZJC[135]	OX1R	Homo sapiens	2016-03-09	Inactive	SB-674042	Antagonist	6TOD
6TP3[134]	OX1R	Homo sapiens	2020-01-01	Inactive	daridorexant	Antagonist	6TOD
6TP6[134]	OX1R	Homo sapiens	2020-01-01	Inactive	filorexant	Antagonist	6TOD
6TOT[134]	OX1R	Homo sapiens	2020-01-15	Inactive	lemborexant	Antagonist	6TOD
4ZJ8[135]	OX1R	Homo sapiens	2016-03-09	Inactive	suvorexant	Antagonist	6TOD
6TO7[134]	OX1R	Homo sapiens	2020-01-01	Inactive	suvorexant	Antagonist	6TOD
5WQC[136]	OX2R	Homo sapiens	2017-11-29	Inactive	EMPA	Antagonist	5WS3
5WS3[136]	OX2R	Homo sapiens	2017-12-13	Inactive	EMPA	Antagonist	5WQC
6TPG[134]	OX2R	Homo sapiens	2020-01-01	Inactive	EMPA	Antagonist	5WQC
6TPN[134]	OX2R	Homo sapiens	2020-01-01	Inactive	HTL6641	Antagonist	5WQC

7L1V[208]	OX2R	Homo sapiens	2021-02-10	Active	compound 1 [PMID: 33547286]	Agonist	7L1U
4S0V[137]	OX2R	Homo sapiens	2015-01-14	Inactive	suvorexant	Antagonist	5WQC
6TPJ[134]	OX2R	Homo sapiens	2020-01-01	Inactive	suvorexant	Antagonist	5WQC
6TPK[209]	OXYR	Homo sapiens	2020-08-05	Inactive	retosiban	Antagonist	7RYC
4XNW[139]	P2RY1	Homo sapiens	2015-04-01	Intermediate	MRS2500	Antagonist	4XNV
4PXZ[140]	P2Y12	Homo sapiens	2014-04-30	Intermediate	2-(methylsulfanyl)adenosine 5'-(trihydrogen diphosphate)	Agonist	4NTJ
4PY0[140]	P2Y12	Homo sapiens	2014-04-30	Intermediate	2-(methylsulfanyl)adenosine 5'-(trihydrogen diphosphate)	Agonist (partial)	4PXZ
4NTJ[141]	P2Y12	Homo sapiens	2014-03-26	Intermediate	AZD1283	Antagonist	4PXZ
3VW7[142]	PAR1	Homo sapiens	2012-12-12	Intermediate	vorapaxar	Antagonist	7TD0
5NDD[143]	PAR2	Homo sapiens	2017-05-03	Intermediate	AZ8838	Antagonist	5NDZ
7M8W[210]	PD2R2	Homo sapiens	2021-08-25	Inactive	15(R)-15-methyl-prostaglandin D_2	Antagonist	6D27
6D27[144]	PD2R2	Homo sapiens	2018-10-03	Inactive	CHEMBL179036	Antagonist	7M8W
6D26[144]	PD2R2	Homo sapiens	2018-10-03	Inactive	fevipiprant	Antagonist	7M8W
7CX2[211]	PE2R2	Homo sapiens	2021-05-05	Active	prostaglandin E_2	Agonist	7CX3
7CX4[211]	PE2R2	Homo sapiens	2021-05-05	Active	evatanepag	Agonist	7CX3
7CX3[211]	PE2R2	Homo sapiens	2021-05-05	Active	taprenepag	Agonist	7CX2
6M9T[145]	PE2R3	Homo sapiens	2018-12-05	Active	misoprostol-FA	Agonist	6AK3
6AK3[146]	PE2R3	Homo sapiens	2018-12-05	Active	prostaglandin E_2	Agonist	6M9T
5YWY[147]	PE2R4	Homo sapiens	2018-12-05	Inactive	ONO-AE3-208	Antagonist	5YHL
5YHL[147]	PE2R4	Homo sapiens	2018-12-05	Inactive	ONO-AE3-208-Br	Antagonist	5YWY
7D7M[212]	PE2R4	Homo sapiens	2020-11-18	Active	prostaglandin E_2	Agonist	5YWY
5ZKQ[148]	PTAFR	Homo sapiens	2018-06-20	Intermediate	ABT-491	Inverse agonist	5ZKP
5ZKP[148]	PTAFR	Homo sapiens	2018-06-20	Other	foropafant	Antagonist	5ZKQ
5X33[149]	Q9WTK1	Cavia porcellus	2018-01-03	Inactive	BIIL 260	Antagonist	NA
7EO2[213]	S1PR1	Homo sapiens	2022-01-05	Active	(S)-FTY720-phosphate	Agonist	7TD4
3V2W[150]	S1PR1	Homo sapiens	2012-02-15	Inactive	W146	Antagonist	3V2Y
3V2Y[150]	S1PR1	Homo sapiens	2012-02-15	Inactive	W146	Antagonist	3V2W
7EO4[213]	S1PR1	Homo sapiens	2022-01-05	Active	siponimod	Agonist	7TD4
7WF7[213]	S1PR1	Homo sapiens	2022-01-05	Active	sphingosine 1-phosphate	Agonist	7TD4
7EW2[214]	S1PR3	Homo sapiens	2021-09-29	Active	(S)-FTY720-phosphate	Agonist	7EW3
7EW4[214]	S1PR3	Homo sapiens	2021-09-29	Active	CYM-5541	Agonist	7EW2
7EW3[214]	S1PR3	Homo sapiens	2021-09-29	Active	sphingosine 1-phosphate	Agonist	7EW2

7C4S[215]	S1PR3	Homo sapiens	2021-06-09	Active	sphingosine 1-phosphate	Agonist	7EW2
7EW1[216]	S1PR5	Homo sapiens	2021-09-29	Active	siponimod	Agonist	NA
6Z10[217]	SUCR1	Rattus norvegicus	2020-09-16	Intermediate	CHEMBL4751488	Antagonist	6RNK
6RNK[151]	SUCR1	Rattus norvegicus	2019-08-14	Intermediate	NF-56-EJ40	Antagonist	6Z10
6IIV[152]	TA2R	Homo sapiens	2018-12-19	Intermediate	daltroban	Antagonist	6IIU
6IIU[152]	TA2R	Homo sapiens	2018-12-19	Intermediate	ramatroban	Antagonist	6IIV

[a]Entries marked “NA” represent cases where a suitable structure for cross-docking was not available.

Table S5. Counts of active and inactive ligands docked into DUD-E experimentally determined structures and homology models.

	Inactive Ligands		Active Ligands			
Receptor	Number of Ligands[a]	Number of Ligands in Subset[b]	Number of IUPHAR[218] Ligands[c]	Number of Ligands in Subset[b]	Subset Agonists	Subset Antagonists
AA2AR	104	25	90	18	9	9
ADRB1	52	25	41	18	9	9
ADRB2	108	25	46	18	9	9
CXCR4	16	15	8	8	0	8
DRD3	5	5	57	18	9	9
Total	285	95	242	80	36	44

[a]Number of ligands docked into each target's experimentally determined structure retrieved from DUD-E.

[b]Number of ligands docked into each target's set of 3 loop-refined homology models.

[c]Number of active ligands retrieved from IUPHAR Guide to Pharmacology[218] per target.

References Cited

1. Yang, D.; De Waal, P.; Dai, A.; Cai, X.; Liu, P.; Yin, Y.; Liu, B.; Caffrey, M.; Melcher, K.; Xu, Y.; et al. A Common Antagonistic Mechanism for Class A GPCRs Revealed by the Structure of the Human 5-HT1B Serotonin Receptor Bound to an Antagonist. *Cell Discov* **2018**, doi:10.2210/PDB5V54/PDB.

2. Wang, C.; Jiang, Y.; Ma, J.; Wu, H.; Wacker, D.; Katritch, V.; Han, G.W.; Liu, W.; Huang, X.P.; Vardy, E.; et al. Structural Basis for Molecular Recognition at Serotonin Receptors. *Science (1979)* **2013**, *340*, 610–614, doi:10.1126/SCIENCE.1232807.

3. García-Nafría, J.; Nehmé, R.; Edwards, P.C.; Tate, C.G. Cryo-EM Structure of the Serotonin 5-HT1B Receptor Coupled to Heterotrimeric Go. *Nature* **2018**, *558*, 620–623, doi:10.1038/S41586-018-0241-9.

4. Kimura, K.T.; Asada, H.; Inoue, A.; Kadji, F.M.N.; Im, D.; Mori, C.; Arakawa, T.; Hirata, K.; Nomura, Y.; Nomura, N. Structures of the 5-HT2A Receptor in Complex with the Antipsychotics Risperidone and Zotepine. *Nat Struct Mol Biol* **2019**, *26*, 121–128.

5. McCorvy, J.D.; Wacker, D.; Wang, S.; Agegnehu, B.; Liu, J.; Lansu, K.; Tribo, A.R.; Olsen, R.H.J.; Che, T.; Jin, J.; et al. Structural Determinants of 5-HT2B Receptor Activation and Biased Agonism. *Nat Struct Mol Biol* **2018**, *25*, 787–796, doi:10.1038/S41594-018-0116-7.

6. Wacker, D.; Wang, S.; McCorvy, J.D.; Betz, R.M.; Venkatakrishnan, A.J.; Levit, A.; Lansu, K.; Schools, Z.L.; Che, T.; Nichols, D.E.; et al. Crystal Structure of an LSD-Bound Human Serotonin Receptor. *Cell* **2017**, *168*, 377-389.e12, doi:10.1016/J.CELL.2016.12.033.

7. Wacker, D.; Wang, C.; Katritch, V.; Han, G.W.; Huang, X.P.; Vardy, E.; McCorvy, J.D.; Jiang, Y.; Chu, M.; Siu, F.Y.; et al. Structural Features for Functional Selectivity at Serotonin Receptors. *Science (1979)* **2013**, *340*, 615–619, doi:10.1126/SCIENCE.1232808.

8. Liu, W.; Wacker, D.; Gati, C.; Han, G.W.; James, D.; Wang, D.; Nelson, G.; Weierstall, U.; Katritch, V.; Barty, A. Serial Femtosecond Crystallography of G Protein–Coupled Receptors. *Science (1979)* **2013**, *342*, 1521–1524.

9. Ishchenko, A.; Wacker, D.; Kapoor, M.; Zhang, A.; Han, G.W.; Basu, S.; Patel, N.; Messerschmidt, M.; Weierstall, U.; Liu, W.; et al. Structural Insights into the Extracellular Recognition of the Human Serotonin 2B Receptor by an Antibody. *Proc Natl Acad Sci U S A* **2017**, *114*, 8223–8228, doi:10.1073/PNAS.1700891114.

10. Peng, Y.; McCorvy, J.D.; Harpsøe, K.; Lansu, K.; Yuan, S.; Popov, P.; Qu, L.; Pu, M.; Che, T.; Nikolajsen, L.F. 5-HT2C Receptor Structures Reveal the Structural Basis of GPCR Polypharmacology. *Cell* **2018**, *172*, 719-730. e14.

11. Glukhova, A.; Thal, D.M.; Nguyen, A.T.; Vecchio, E.A.; Jörg, M.; Scammells, P.J.; May, L.T.; Sexton, P.M.; Christopoulos, A. Structure of the Adenosine A1 Receptor Reveals the Basis for Subtype Selectivity. *Cell* **2017**, *168*, 867-877.e13, doi:10.1016/J.CELL.2017.01.042.

12. Cheng, R.K.Y.; Segala, E.; Robertson, N.; Deflorian, F.; Doré, A.S.; Errey, J.C.; Fiez-Vandal, C.; Marshall, F.H.; Cooke, R.M. Structures of Human A1 and A2A Adenosine Receptors with Xanthines Reveal Determinants of Selectivity. *Structure* **2017**, *25*, 1275-1285.e4, doi:10.1016/J.STR.2017.06.012.

13. Draper-Joyce, C.J.; Khoshouei, M.; Thal, D.M.; Liang, Y.L.; Nguyen, A.T.N.; Furness, S.G.B.; Venugopal, H.; Baltos, J.A.; Plitzko, J.M.; Danev, R.; et al. Structure of the Adenosine-Bound Human Adenosine A1 Receptor-Gi Complex. *Nature* **2018**, *558*, 559–565, doi:10.1038/S41586-018-0236-6.

14. Segala, E.; Guo, D.; Cheng, R.K.Y.; Bortolato, A.; Deflorian, F.; Doré, A.S.; Errey, J.C.; Heitman, L.H.; Ijzerman, A.P.; Marshall, F.H.; et al. Controlling the Dissociation of Ligands from the Adenosine A2A Receptor through Modulation of Salt Bridge Strength. *J Med Chem* **2016**, *59*, 6470–6479, doi:10.1021/ACS.JMEDCHEM.6B00653.

15. Sun, B.; Bachhawat, P.; Chu, M.L.H.; Wood, M.; Ceska, T.; Sands, Z.A.; Mercier, J.; Lebon, F.; Kobilka, T.S.; Kobilka, B.K. Crystal Structure of the Adenosine A2A Receptor Bound to an Antagonist Reveals a Potential Allosteric Pocket. *Proc Natl Acad Sci U S A* **2017**, *114*, 2066–2071, doi:10.1073/PNAS.1621423114.

16. Lebon, G.; Edwards, P.C.; Leslie, A.G.W.; Tate, C.G. Molecular Determinants of CGS21680 Binding to the Human Adenosine A2A Receptor. *Mol Pharmacol* **2015**, *87*, 907–915, doi:10.1124/MOL.114.097360.

17. Rucktooa, P.; Cheng, R.K.Y.; Segala, E.; Geng, T.; Errey, J.C.; Brown, G.A.; Cooke, R.M.; Marshall, F.H.; Doré, A.S. Towards High Throughput GPCR Crystallography: In Meso Soaking of Adenosine A2A Receptor Crystals. *Sci Rep* **2018**, *8*, doi:10.1038/S41598-017-18570-W.

18. Congreve, M.; Andrews, S.P.; Doré, A.S.; Hollenstein, K.; Hurrell, E.; Langmead, C.J.; Mason, J.S.; Ng, I.W.; Tehan, B.; Zhukov, A.; et al. Discovery of 1,2,4-Triazine Derivatives as Adenosine A 2A Antagonists Using Structure Based Drug Design. *J Med Chem* **2012**, *55*, 1898–1903, doi:10.1021/JM201376W.

19. Lebon, G.; Warne, T.; Edwards, P.C.; Bennett, K.; Langmead, C.J.; Leslie, A.G.W.; Tate, C.G. Agonist-Bound Adenosine A2A Receptor Structures Reveal Common Features of GPCR Activation. *Nature* **2011**, *474*, 521–526, doi:10.1038/NATURE10136.

20. Carpenter, B.; Nehmé, R.; Warne, T.; Leslie, A.G.W.; Tate, C.G. Structure of the Adenosine A2A Receptor Bound to an Engineered G Protein. *Nature* **2016**, *536*, 104–107, doi:10.1038/NATURE18966.

21. García-Nafría, J.; Lee, Y.; Bai, X.; Carpenter, B.; Tate, C.G. Cryo-EM Structure of the Adenosine A2A Receptor Coupled to an Engineered Heterotrimeric G Protein. *Elife* **2018**, *7*, doi:10.7554/ELIFE.35946.

22. Xu, F.; Wu, H.; Katritch, V.; Han, G.W.; Jacobson, K.A.; Gao, Z.G.; Cherezov, V.; Stevens, R.C. Structure of an Agonist-Bound Human A2A Adenosine Receptor. *Science (1979)* **2011**, *332*, 322–327, doi:10.1126/SCIENCE.1202793.

23. White, K.L.; Eddy, M.T.; Gao, Z.G.; Han, G.W.; Lian, T.; Deary, A.; Patel, N.; Jacobson, K.A.; Katritch, V.; Stevens, R.C. Structural Connection between Activation Microswitch and Allosteric Sodium Site in GPCR Signaling. *Structure* **2018**, *26*, 259-269.e5, doi:10.1016/J.STR.2017.12.013.

24. Jaakola, V.P.; Griffith, M.T.; Hanson, M.A.; Cherezov, V.; Chien, E.Y.T.; Lane, J.R.; Ijzerman, A.P.; Stevens, R.C. The 2.6 Angstrom Crystal Structure of a Human A2A Adenosine Receptor Bound to an Antagonist. *Science (1979)* **2008**, *322*, 1211–1217, doi:10.1126/SCIENCE.1164772.

25. Doré, A.S.; Robertson, N.; Errey, J.C.; Ng, I.; Hollenstein, K.; Tehan, B.; Hurrell, E.; Bennett, K.; Congreve, M.; Magnani, F.; et al. Structure of the Adenosine A 2A Receptor in Complex with ZM241385 and the Xanthines XAC and Caffeine. *Structure* **2011**, *19*, 1283–1293, doi:10.1016/J.STR.2011.06.014.

26. Hino, T.; Arakawa, T.; Iwanari, H.; Yurugi-Kobayashi, T.; Ikeda-Suno, C.; Nakada-Nakura, Y.; Kusano-Arai, O.; Weyand, S.; Shimamura, T.; Nomura, N.; et al. G-Protein-Coupled Receptor Inactivation by an Allosteric Inverse-Agonist Antibody. *Nature* **2012**, *482*, 237–240, doi:10.1038/NATURE10750.

27. Liu, W.; Chun, E.; Thompson, A.A.; Chubukov, P.; Xu, F.; Katritch, V.; Han, G.W.; Roth, C.B.; Heitman, L.H.; IJzerman, A.P.; et al. Structural Basis for Allosteric Regulation of GPCRS by Sodium Ions. *Science (1979)* **2012**, *337*, 232–236, doi:10.1126/SCIENCE.1219218.

28. Melnikov, I.; Polovinkin, V.; Kovalev, K.; Gushchin, I.; Shevtsov, M.; Shevchenko, V.; Mishin, A.; Alekseev, A.; Rodriguez-Valera, F.; Borshchevskiy, V.; et al. Fast Iodide-SAD Phasing for High-Throughput Membrane Protein Structure Determination. *Sci Adv* **2017**, *3*, doi:10.1126/SCIADV.1602952.

29. Batyuk, A.; Galli, L.; Ishchenko, A.; Han, G.W.; Gati, C.; Popov, P.A.; Lee, M.Y.; Stauch, B.; White, T.A.; Barty, A.; et al. Native Phasing of X-Ray Free-Electron Laser Data for a G Protein–Coupled Receptor. *Sci Adv* **2016**, *2*, doi:10.1126/SCIADV.1600292.

30. Weinert, T.; Olieric, N.; Cheng, R.; Brünle, S.; James, D.; Ozerov, D.; Gashi, D.; Vera, L.; Marsh, M.; Jaeger, K.; et al. Serial Millisecond Crystallography for Routine Room-Temperature Structure Determination at Synchrotrons. *Nat Commun* **2017**, *8*, doi:10.1038/S41467-017-00630-4.

31. Martin-Garcia, J.M.; Conrad, C.E.; Nelson, G.; Stander, N.; Zatsepin, N.A.; Zook, J.; Zhu, L.; Geiger, J.; Chun, E.; Kissick, D.; et al. Serial Millisecond Crystallography of Membrane and Soluble Protein Microcrystals Using Synchrotron Radiation. *IUCrJ* **2017**, *4*, 439–454, doi:10.1107/S205225251700570X.

32. Broecker, J.; Morizumi, T.; Ou, W.L.; Klingel, V.; Kuo, A.; Kissick, D.J.; Ishchenko, A.; Lee, M.Y.; Xu, S.; Makarov, O.; et al. High-Throughput in Situ X-Ray Screening of and Data Collection from Protein Crystals at Room Temperature and under Cryogenic Conditions. *Nat Protoc* **2018**, *13*, 260–291, doi:10.1038/NPROT.2017.135.

33. Eddy, M.T.; Lee, M.Y.; Gao, Z.G.; White, K.L.; Didenko, T.; Horst, R.; Audet, M.; Stanczak, P.; McClary, K.M.; Han, G.W.; et al. Allosteric Coupling of Drug Binding and Intracellular Signaling in the A2A Adenosine Receptor. *Cell* **2018**, *172*, 68-80.e12, doi:10.1016/J.CELL.2017.12.004.

34. Thal, D.M.; Sun, B.; Feng, D.; Nawaratne, V.; Leach, K.; Felder, C.C.; Bures, M.G.; Evans, D.A.; Weis, W.I.; Bachhawat, P. Crystal Structures of the M1 and M4 Muscarinic Acetylcholine Receptors. *Nature* **2016**, *531*, 335–340.

35. Maeda, S.; Qu, Q.; Robertson, M.J.; Skiniotis, G.; Kobilka, B.K. Structures of the M1 and M2 Muscarinic Acetylcholine Receptor/G-Protein Complexes. *Science (1979)* **2019**, *364*, 552–557, doi:10.1126/SCIENCE.AAW5188.

36. Suno, R.; Lee, S.; Maeda, S.; Yasuda, S.; Yamashita, K.; Hirata, K.; Horita, S.; Tawaramoto, M.S.; Tsujimoto, H.; Murata, T.; et al. Structural Insights into the Subtype-Selective Antagonist Binding to the M2 Muscarinic Receptor. *Nat Chem Biol* **2018**, *14*, 1150–1158, doi:10.1038/S41589-018-0152-Y.

37. Haga, K.; Kruse, A.C.; Asada, H.; Yurugi-Kobayashi, T.; Shiroishi, M.; Zhang, C.; Weis, W.I.; Okada, T.; Kobilka, B.K.; Haga, T.; et al. Structure of the Human M2 Muscarinic Acetylcholine Receptor Bound to an Antagonist. *Nature* **2012**, *482*, 547–551, doi:10.1038/NATURE10753.

38. Kruse, A.C.; Ring, A.M.; Manglik, A.; Hu, J.; Hu, K.; Eitel, K.; Hübner, H.; Pardon, E.; Valant, C.; Sexton, P.M.; et al. Activation and Allosteric Modulation of a Muscarinic Acetylcholine Receptor. *Nature* **2013**, *504*, 101–106, doi:10.1038/NATURE12735.

39. Liu, H.; Hofmann, J.; Fish, I.; Schaake, B.; Eitel, K.; Bartuschat, A.; Kaindl, J.; Rampp, H.; Banerjee, A.; Hübner, H. Structure-Guided Development of Selective M3 Muscarinic Acetylcholine Receptor Antagonists. *Proceedings of the National Academy of Sciences* **2018**, *115*, 12046–12050.

40. Thorsen, T.S.; Matt, R.; Weis, W.I.; Kobilka, B.K. Modified T4 Lysozyme Fusion Proteins Facilitate G Protein-Coupled Receptor Crystallogenesis. *Structure* **2014**, *22*, 1657–1664.

41. Kruse, A.C.; Hu, J.; Pan, A.C.; Arlow, D.H.; Rosenbaum, D.M.; Rosemond, E.; Green, H.F.; Liu, T.; Chae, P.S.; Dror, R.O. Structure and Dynamics of the M3 Muscarinic Acetylcholine Receptor. *Nature* **2012**, *482*, 552–556.

42. Vuckovic, Z.; Gentry, P.R.; Berizzi, A.E.; Hirata, K.; Varghese, S.; Thompson, G.; van der Westhuizen, E.T.; Burger, W.A.C.; Rahmani, R.; Valant, C. Crystal Structure of the M5 Muscarinic Acetylcholine Receptor. *Proceedings of the National Academy of Sciences* **2019**, *116*, 26001–26007.

43. Warne, T.; Serrano-Vega, M.J.; Baker, J.G.; Moukhametzianov, R.; Edwards, P.C.; Henderson, R.; Leslie, A.G.W.; Tate, C.G.; Schertler, G.F.X. Structure of a B1-Adrenergic G-Protein-Coupled Receptor. *Nature* **2008**, *454*, 486–491, doi:10.1038/NATURE07101.

44. Moukhametzianov, R.; Warne, T.; Edwards, P.C.; Serrano-Vega, M.J.; Leslie, A.G.W.; Tate, C.G.; Schertler, G.F.X. Two Distinct Conformations of Helix 6 Observed in Antagonist-Bound Structures of a Β1-Adrenergic Receptor. *Proc Natl Acad Sci U S A* **2011**, *108*, 8228–8232, doi:10.1073/PNAS.1100185108.

45. Miller-Gallacher, J.L.; Nehmé, R.; Warne, T.; Edwards, P.C.; Schertler, G.F.X.; Leslie, A.G.W.; Tate, C.G. The 2.1 Å Resolution Structure of Cyanopindolol-Bound Β1-Adrenoceptor Identifies an Intramembrane Na+ Ion That Stabilises the Ligand-Free Receptor. *PLoS One* **2014**, *9*, doi:10.1371/JOURNAL.PONE.0092727.

46. Leslie, A.G.W.; Warne, T.; Tate, C.G. Ligand Occupancy in Crystal Structure of β 1-Adrenergic G Protein Coupled Receptor. *Nat Struct Mol Biol* **2015**, *22*, 941–942, doi:10.1038/NSMB.3130.

47. Warne, T.; Edwards, P.C.; Dorfe, A.S.; Leslie, A.G.W.; Tate, C.G. Molecular Basis for High-Affinity Agonist Binding in GPCRs. *Science (1979)* **2019**, doi:10.1126/SCIENCE.AAU5595.

48. Sato, T.; Baker, J.; Warne, T.; Brown, G.A.; Leslie, A.G.W.; Congreve, M.; Tate, C.G. Pharmacological Analysis and Structure Determination of 7-Methylcyanopindolol-Bound Β1-Adrenergic Receptor. *Mol Pharmacol* **2015**, *88*, 1024–1034, doi:10.1124/MOL.115.101030.

49. Warne, T.; Edwards, P.C.; Leslie, A.G.W.; Tate, C.G. Crystal Structures of a Stabilized Β1-Adrenoceptor Bound to the Biased Agonists Bucindolol and Carvedilol. *Structure* **2012**, *20*, 841–849, doi:10.1016/J.STR.2012.03.014.

50. Warne, T.; Moukhametzianov, R.; Baker, J.G.; Nehmé, R.; Edwards, P.C.; Leslie, A.G.W.; Schertler, G.F.X.; Tate, C.G. The Structural Basis for Agonist and Partial Agonist Action on a Β1-Adrenergic Receptor. *Nature* **2011**, *469*, 241–245, doi:10.1038/NATURE09746.

51. Christopher, J.A.; Brown, J.; Doré, A.S.; Errey, J.C.; Koglin, M.; Marshall, F.H.; Myszka, D.G.; Rich, R.L.; Tate, C.G.; Tehan, B.; et al. Biophysical Fragment Screening of the Β1-Adrenergic Receptor: Identification of High Affinity Arylpiperazine Leads Using Structure-Based Drug Design. *J Med Chem* **2013**, *56*, 3446–3455, doi:10.1021/JM400140Q.

52. Lee, Y.; Warne, T.; Nehmé, R.; Pandey, S.; Dwivedi-Agnihotri, H.; Chaturvedi, M.; Edwards, P.C.; García-Nafría, J.; Leslie, A.G.W.; Shukla, A.K.; et al. Molecular Basis of β-Arrestin Coupling to Formoterol-Bound Β1-Adrenoceptor. *Nature* **2020**, *583*, 862–866, doi:10.1038/S41586-020-2419-1.

53. Rasmussen, S.G.F.; Choi, H.J.; Fung, J.J.; Pardon, E.; Casarosa, P.; Chae, P.S.; Devree, B.T.; Rosenbaum, D.M.; Thian, F.S.; Kobilka, T.S.; et al. Structure of a Nanobody-Stabilized Active State of the Β2 Adrenoceptor. *Nature* **2011**, *469*, 175–181, doi:10.1038/NATURE09648.

54. Rasmussen, S.G.F.; Devree, B.T.; Zou, Y.; Kruse, A.C.; Chung, K.Y.; Kobilka, T.S.; Thian, F.S.; Chae, P.S.; Pardon, E.; Calinski, D.; et al. Crystal Structure of the β 2 Adrenergic Receptor-Gs Protein Complex. *Nature* **2011**, *477*, 549–557, doi:10.1038/NATURE10361.

55. Ring, A.M.; Manglik, A.; Kruse, A.C.; Enos, M.D.; Weis, W.I.; Garcia, K.C.; Kobilka, B.K. Adrenaline-Activated Structure of β 2-Adrenoceptor Stabilized by an Engineered Nanobody. *Nature* **2013**, *502*, 575–579, doi:10.1038/NATURE12572.

56. Wacker, D.; Fenalti, G.; Brown, M.A.; Katritch, V.; Abagyan, R.; Cherezov, V.; Stevens, R.C. Conserved Binding Mode of Human Β2 Adrenergic Receptor Inverse Agonists and Antagonist Revealed by X-Ray Crystallography. *J Am Chem Soc* **2010**, *132*, 11443–11445, doi:10.1021/JA105108Q.

57. Cherezov, V.; Rosenbaum, D.M.; Hanson, M.A.; Rasmussen, S.G.F.; Thian, F.S.; Kobilka, T.S.; Choi, H.-J.; Kuhn, P.; Weis, W.I.; Kobilka, B.K. High-Resolution Crystal Structure of an Engineered Human Β2-Adrenergic G Protein–Coupled Receptor. *Science (1979)* **2007**, *318*, 1258–1265.

58. Zou, Y.; Weis, W.I.; Kobilka, B.K. N-Terminal T4 Lysozyme Fusion Facilitates Crystallization of a G Protein Coupled Receptor. *PLoS One* **2012**, *7*, doi:10.1371/JOURNAL.PONE.0046039.

59. Huang, C.Y.; Olieric, V.; Ma, P.; Howe, N.; Vogeley, L.; Liu, X.; Warshamanage, R.; Weinert, T.; Panepucci, E.; Kobilka, B.; et al. In Meso in Situ Serial X-Ray Crystallography of Soluble and Membrane Proteins at Cryogenic Temperatures. *Acta Crystallogr D Struct Biol* **2016**, *72*, 93–112, doi:10.1107/S2059798315021683.

60. Ma, P.; Weichert, D.; Aleksandrov, L.A.; Jensen, T.J.; Riordan, J.R.; Liu, X.; Kobilka, B.K.; Caffrey, M. The Cubicon Method for Concentrating Membrane Proteins in the Cubic Mesophase. *Nat Protoc* **2017**, *12*, 1745–1762, doi:10.1038/NPROT.2017.057.

61. Staus, D.P.; Strachan, R.T.; Manglik, A.; Pani, B.; Kahsai, A.W.; Kim, T.H.; Wingler, L.M.; Ahn, S.; Chatterjee, A.; Masoudi, A.; et al. Allosteric Nanobodies Reveal the Dynamic Range and Diverse Mechanisms of G-Protein-Coupled Receptor Activation. *Nature* **2016**, *535*, 448–452, doi:10.1038/NATURE18636.

62. Rosenbaum, D.M.; Zhang, C.; Lyons, J.A.; Holl, R.; Aragao, D.; Arlow, D.H.; Rasmussen, S.G.F.; Choi, H.J.; Devree, B.T.; Sunahara, R.K.; et al. Structure and Function of an Irreversible Agonist-Β2 Adrenoceptor Complex. *Nature* **2011**, *469*, 236–242, doi:10.1038/NATURE09665.

63. Weichert, D.; Kruse, A.C.; Manglik, A.; Hiller, C.; Zhang, C.; Hübner, H.; Kobilka, B.K.; Gmeiner, P. Covalent Agonists for Studying G Protein-Coupled Receptor Activation. *Proc Natl Acad Sci U S A* **2014**, *111*, 10744–10748, doi:10.1073/PNAS.1410415111.

64. Masureel, M.; Zou, Y.; Picard, L.P.; van der Westhuizen, E.; Mahoney, J.P.; Rodrigues, J.P.G.L.M.; Mildorf, T.J.; Dror, R.O.; Shaw, D.E.; Bouvier, M.; et al. Structural Insights into Binding Specificity, Efficacy and Bias of a β 2 AR Partial Agonist. *Nat Chem Biol* **2018**, *14*, 1059–1066, doi:10.1038/S41589-018-0145-X.

65. Hanson, M.A.; Cherezov, V.; Griffith, M.T.; Roth, C.B.; Jaakola, V.P.; Chien, E.Y.T.; Velasquez, J.; Kuhn, P.; Stevens, R.C. A Specific Cholesterol Binding Site Is Established by the 2.8 Å Structure of the Human Β2-Adrenergic Receptor. *Structure* **2008**, *16*, 897–905, doi:10.1016/J.STR.2008.05.001.

66. Liu, X.; Ahn, S.; Kahsai, A.W.; Meng, K.-C.; Latorraca, N.R.; Pani, B.; Venkatakrishnan, A.J.; Masoudi, A.; Weis, W.I.; Dror, R.O.; et al. Mechanism of Intracellular Allosteric Β2AR Antagonist Revealed by X-Ray Crystal Structure. *Nature* **2017**, *548*, 480–484, doi:10.1038/nature23652.

67. Zhang, H.; Unal, H.; Desnoyer, R.; Han, G.W.; Patel, N.; Katritch, V.; Karnik, S.S.; Cherezov, V.; Stevens, R.C. Structural Basis for Ligand Recognition and Functional Selectivity at Angiotensin Receptor. *Journal of Biological Chemistry* **2015**, *290*, 29127–29139, doi:10.1074/JBC.M115.689000.

68. Zhang, H.; Unal, H.; Gati, C.; Han, G.W.; Liu, W.; Zatsepin, N.A.; James, D.; Wang, D.; Nelson, G.; Weierstall, U.; et al. Structure of the Angiotensin Receptor Revealed by Serial Femtosecond Crystallography. *Cell* **2015**, *161*, 833–844, doi:10.1016/J.CELL.2015.04.011.

69. Wingler, L.M.; McMahon, C.; Staus, D.P.; Lefkowitz, R.J.; Kruse, A.C. Distinctive Activation Mechanism for Angiotensin Receptor Revealed by a Synthetic Nanobody. *Cell* **2019**, *176*, 479-490.e12, doi:10.1016/j.cell.2018.12.006.

70. Zhang, H.; Han, G.W.; Batyuk, A.; Ishchenko, A.; White, K.L.; Patel, N.; Sadybekov, A.; Zamlynny, B.; Rudd, M.T.; Hollenstein, K. Structural Basis for Selectivity and Diversity in Angiotensin II Receptors. *Nature* **2017**, *544*, 327.

71. Asada, H.; Inoue, A.; Ngako Kadji, F.M.; Hirata, K.; Shiimura, Y.; Im, D.; Shimamura, T.; Nomura, N.; Iwanari, H.; Hamakubo, T.; et al. The Crystal Structure of Angiotensin II Type 2 Receptor with Endogenous Peptide Hormone. *Structure* **2020**, *28*, 418-425.e4, doi:10.1016/j.str.2019.12.003.

72. Asada, H.; Horita, S.; Hirata, K.; Shiroishi, M.; Shiimura, Y.; Iwanari, H.; Hamakubo, T.; Shimamura, T.; Nomura, N.; Kusano-Arai, O.; et al. Crystal Structure of the Human Angiotensin Ii Type 2 Receptor Bound to an Angiotensin Ii Analog. *Nat Struct Mol Biol* **2018**, *25*, 570–576, doi:10.1038/s41594-018-0079-8.

73. Ma, Y.; Ding, Y.; Song, X.; Ma, X.; Li, X.; Zhang, N.; Song, Y.; Sun, Y.; Shen, Y.; Zhong, W.; et al. Structure-Guided Discovery of a Single-Domain Antibody Agonist against Human Apelin Receptor. *Sci Adv* **2020**, *6*, doi:10.1126/sciadv.aax7379.

74. Ma, Y.; Yue, Y.; Ma, Y.; Zhang, Q.; Zhou, Q.; Song, Y.; Shen, Y.; Li, X.; Ma, X.; Li, C.; et al. Structural Basis for Apelin Control of the Human Apelin Receptor. *Structure* **2017**, *25*, 858-866.e4, doi:10.1016/j.str.2017.04.008.

75. Robertson, N.; Rappas, M.; Doré, A.S.; Brown, J.; Bottegoni, G.; Koglin, M.; Cansfield, J.; Jazayeri, A.; Cooke, R.M.; Marshall, F.H. Structure of the Complement C5a Receptor Bound to the Extra-Helical Antagonist NDT9513727. *Nature* **2018**, *553*, 111–114.

76. Liu, H.; Kim, H.R.; Deepak, R.N.V.K.; Wang, L.; Chung, K.Y.; Fan, H.; Wei, Z.; Zhang, C. Orthosteric and Allosteric Action of the C5a Receptor Antagonists. *Nat Struct Mol Biol* **2018**, *25*, 472–481, doi:10.1038/s41594-018-0067-z.

77. Apel, A.-K.; Cheng, R.K.Y.; Tautermann, C.S.; Brauchle, M.; Huang, C.-Y.; Pautsch, A.; Hennig, M.; Nar, H.; Schnapp, G. Crystal Structure of CC Chemokine Receptor 2A in Complex with an Orthosteric Antagonist Provides Insights for the Design of Selective Antagonists. *Structure* **2019**, *27*, 427–438.

78. Zheng, Y.; Qin, L.; Zacarías, N.V.O.; De Vries, H.; Han, G.W.; Gustavsson, M.; Dabros, M.; Zhao, C.; Cherney, R.J.; Carter, P.; et al. Structure of CC Chemokine Receptor 2 with Orthosteric and Allosteric Antagonists. *Nature* **2016**, *540*, 458–461, doi:10.1038/nature20605.

79. Peng, P.; Chen, H.; Zhu, Y.; Wang, Z.; Li, J.; Luo, R.-H.; Wang, J.; Chen, L.; Yang, L.-M.; Jiang, H. Structure-Based Design of 1-Heteroaryl-1, 3-Propanediamine Derivatives as a Novel Series of CC-Chemokine Receptor 5 Antagonists. *J Med Chem* **2018**, *61*, 9621–9636.

80. Tan, Q.; Zhu, Y.; Li, J.; Chen, Z.; Han, G.W.; Kufareva, I.; Li, T.; Ma, L.; Fenalti, G.; Li, J. Structure of the CCR5 Chemokine Receptor–HIV Entry Inhibitor Maraviroc Complex. *Science (1979)* **2013**, *341*, 1387–1390.

81. Shaik, M.M.; Peng, H.; Lu, J.; Rits-Volloch, S.; Xu, C.; Liao, M.; Chen, B. Structural Basis of Coreceptor Recognition by HIV-1 Envelope Spike. *Nature* **2019**, *565*, 318–323, doi:10.1038/s41586-018-0804-9.

82. Zheng, Y.; Han, G.W.; Abagyan, R.; Wu, B.; Stevens, R.C.; Cherezov, V.; Kufareva, I.; Handel, T.M. Structure of CC Chemokine Receptor 5 with a Potent Chemokine Antagonist Reveals Mechanisms of Chemokine Recognition and Molecular Mimicry by HIV. *Immunity* **2017**, *46*, 1005-1017.e5, doi:10.1016/j.immuni.2017.05.002.

83. Jaeger, K.; Bruenle, S.; Weinert, T.; Guba, W.; Muehle, J.; Miyazaki, T.; Weber, M.; Furrer, A.; Haenggi, N.; Tetaz, T.; et al. Structural Basis for Allosteric Ligand Recognition in the Human CC Chemokine Receptor 7. *Cell* **2019**, *178*, 1222-1230.e10, doi:10.1016/j.cell.2019.07.028.

84. Oswald, C.; Rappas, M.; Kean, J.; Doré, A.S.; Errey, J.C.; Bennett, K.; Deflorian, F.; Christopher, J.A.; Jazayeri, A.; Mason, J.S.; et al. Intracellular Allosteric Antagonism of the CCR9 Receptor. *Nature* **2016**, *540*, 462–465, doi:10.1038/nature20606.

85. Luginina, A.; Gusach, A.; Marin, E.; Mishin, A.; Brouillette, R.; Popov, P.; Shiriaeva, A.; Besserer-Offroy, É.; Longpré, J.-M.; Lyapina, E. Structure-Based Mechanism of Cysteinyl Leukotriene Receptor Inhibition by Antiasthmatic Drugs. *Sci Adv* **2019**, *5*, eaax2518.

86. Hua, T.; Vemuri, K.; Nikas, S.P.; Laprairie, R.B.; Wu, Y.; Qu, L.; Pu, M.; Korde, A.; Jiang, S.; Ho, J.-H. Crystal Structures of Agonist-Bound Human Cannabinoid Receptor CB1. *Nature* **2017**, *547*, 468–471.

87. Hua, T.; Vemuri, K.; Pu, M.; Qu, L.; Han, G.W.; Wu, Y.; Zhao, S.; Shui, W.; Li, S.; Korde, A. Crystal Structure of the Human Cannabinoid Receptor CB1. *Cell* **2016**, *167*, 750–762.

88. Kumar, K.K.; Shalev-Benami, M.; Robertson, M.J.; Hu, H.; Banister, S.D.; Hollingsworth, S.A.; Latorraca, N.R.; Kato, H.E.; Hilger, D.; Maeda, S. Structure of a Signaling Cannabinoid Receptor 1-G Protein Complex. *Cell* **2019**, *176*, 448–458.

89. Shao, Z.; Yin, J.; Chapman, K.; Grzemska, M.; Clark, L.; Wang, J.; Rosenbaum, D.M. High-Resolution Crystal Structure of the Human CB1 Cannabinoid Receptor. *Nature* **2016**, *540*, 602–606.

90. Li, X.; Hua, T.; Vemuri, K.; Ho, J.-H.; Wu, Y.; Wu, L.; Popov, P.; Benchama, O.; Zvonok, N.; Qu, L. Crystal Structure of the Human Cannabinoid Receptor CB2. *Cell* **2019**, *176*, 459–467.

91. Hua, T.; Li, X.; Wu, L.; Iliopoulos-Tsoutsouvas, C.; Wang, Y.; Wu, M.; Shen, L.; Brust, C.A.; Nikas, S.P.; Song, F. Activation and Signaling Mechanism Revealed by Cannabinoid Receptor-Gi Complex Structures. *Cell* **2020**, *180*, 655–665.

92. Xing, C.; Zhuang, Y.; Xu, T.-H.; Feng, Z.; Zhou, X.E.; Chen, M.; Wang, L.; Meng, X.; Xue, Y.; Wang, J. Cryo-EM Structure of the Human Cannabinoid Receptor CB2-Gi Signaling Complex. *Cell* **2020**, *180*, 645–654.

93. Wu, B.; Chien, E.Y.T.; Mol, C.D.; Fenalti, G.; Liu, W.; Katritch, V.; Abagyan, R.; Brooun, A.; Wells, P.; Bi, F.C.; et al. Structures of the CXCR4 Chemokine GPCR with Small-Molecule and Cyclic Peptide Antagonists. *Science* **2010**, *330*, 1066–1071, doi:10.1126/science.1194396.

94. Qin, L.; Kufareva, I.; Holden, L.G.; Wang, C.; Zheng, Y.; Zhao, C.; Fenalti, G.; Wu, H.; Han, G.W.; Cherezov, V.; et al. Crystal Structure of the Chemokine Receptor CXCR4 in Complex with a Viral Chemokine. *Science (1979)* **2015**, *347*, 1117–1122, doi:10.1126/science.1261064.

95. Fan, L.; Tan, L.; Chen, Z.; Qi, J.; Nie, F.; Luo, Z.; Cheng, J.; Wang, S. Haloperidol Bound D 2 Dopamine Receptor Structure Inspired the Discovery of Subtype Selective Ligands. *Nat Commun* **2020**, *11*, 1–11.

96. Wang, S.; Che, T.; Levit, A.; Shoichet, B.K.; Wacker, D.; Roth, B.L. Structure of the D2 Dopamine Receptor Bound to the Atypical Antipsychotic Drug Risperidone. *Nature* **2018**, *555*, 269–273.

97. Chien, E.Y.T.; Liu, W.; Zhao, Q.; Katritch, V.; Han, G.W.; Hanson, M.A.; Shi, L.; Newman, A.H.; Javitch, J.A.; Cherezov, V. Structure of the Human Dopamine D3 Receptor in Complex with a D2/D3 Selective Antagonist. *Science (1979)* **2010**, *330*, 1091–1095.

98. Wang, S.; Wacker, D.; Levit, A.; Che, T.; Betz, R.M.; McCorvy, J.D.; Venkatakrishnan, A.J.; Huang, X.-P.; Dror, R.O.; Shoichet, B.K. D4 Dopamine Receptor High-Resolution Structures Enable the Discovery of Selective Agonists. *Science (1979)* **2017**, *358*, 381–386.

99. Nagiri, C.; Shihoya, W.; Inoue, A.; Kadji, F.M.N.; Aoki, J.; Nureki, O. Crystal Structure of Human Endothelin ETB Receptor in Complex with Peptide Inverse Agonist IRL2500. *Commun Biol* **2019**, *2*, 236.

100. Shihoya, W.; Nishizawa, T.; Yamashita, K.; Inoue, A.; Hirata, K.; Kadji, F.M.N.; Okuta, A.; Tani, K.; Aoki, J.; Fujiyoshi, Y. X-Ray Structures of Endothelin ETB Receptor Bound to Clinical Antagonist Bosentan and Its Analog. *Nat Struct Mol Biol* **2017**, *24*, 758–764.

101. Shihoya, W.; Nishizawa, T.; Okuta, A.; Tani, K.; Dohmae, N.; Fujiyoshi, Y.; Nureki, O.; Doi, T. Activation Mechanism of Endothelin ET B Receptor by Endothelin-1. *Nature* **2016**, *537*, 363–368, doi:10.1038/nature19319.

102. Shihoya, W.; Izume, T.; Inoue, A.; Yamashita, K.; Kadji, F.M.N.; Hirata, K.; Aoki, J.; Nishizawa, T.; Nureki, O. Crystal Structures of Human ETB Receptor Provide Mechanistic Insight into Receptor Activation and Partial Activation. *Nat Commun* **2018**, *9*, doi:10.1038/s41467-018-07094-0.

103. Nagiri, C.; Shihoya, W.; Inoue, A.; Kadji, F.M.N.; Aoki, J.; Nureki, O. Crystal Structure of Human Endothelin ETB Receptor in Complex with Peptide Inverse Agonist IRL2500. *Commun Biol* **2019**, *2*, doi:10.1038/s42003-019-0482-7.

104. Izume, T.; Miyauchi, H.; Shihoya, W.; Nureki, O. Crystal Structure of Human Endothelin ETB Receptor in Complex with Sarafotoxin S6b. *Biochem Biophys Res Commun* **2020**, *528*, 383–388, doi:10.1016/j.bbrc.2019.12.091.

105. Lu, J.; Byrne, N.; Wang, J.; Bricogne, G.; Brown, F.K.; Chobanian, H.R.; Colletti, S.L.; Salvo, J. Di; Thomas-Fowlkes, B.; Guo, Y. Structural Basis for the Cooperative Allosteric Activation of the Free Fatty Acid Receptor GPR40. *Nat. Struct. Mol. Biol.* **2017**, *24*, 570.

106. Ho, J.D.; Chau, B.; Rodgers, L.; Lu, F.; Wilbur, K.L.; Otto, K.A.; Chen, Y.; Song, M.; Riley, J.P.; Yang, H.-C.; et al. Structural Basis for GPR40 Allosteric Agonism and Incretin Stimulation. *Nat Commun* **2018**, *9*, doi:10.1038/s41467-017-01240-w.

107. Srivastava, A.; Yano, J.; Hirozane, Y.; Kefala, G.; Gruswitz, F.; Snell, G.; Lane, W.; Ivetac, A.; Aertgeerts, K.; Nguyen, J. High-Resolution Structure of the Human GPR40 Receptor Bound to Allosteric Agonist TAK-875. *Nature* **2014**, *513*, 124–127.

108. Zhuang, Y.; Liu, H.; Edward Zhou, X.; Kumar Verma, R.; de Waal, P.W.; Jang, W.; Xu, T.-H.; Wang, L.; Meng, X.; Zhao, G.; et al. Structure of Formylpeptide Receptor 2-G<inf>i</Inf> Complex Reveals Insights into Ligand Recognition and Signaling. *Nat Commun* **2020**, *11*, doi:10.1038/s41467-020-14728-9.

109. Chen, T.; Xiong, M.; Zong, X.; Ge, Y.; Zhang, H.; Wang, M.; Won Han, G.; Yi, C.; Ma, L.; Ye, R.D.; et al. Structural Basis of Ligand Binding Modes at the Human Formyl Peptide Receptor 2. *Nat Commun* **2020**, *11*, doi:10.1038/s41467-020-15009-1.

110. Lin, X.; Li, M.; Wang, N.; Wu, Y.; Luo, Z.; Guo, S.; Han, G.-W.; Li, S.; Yue, Y.; Wei, X. Structural Basis of Ligand Recognition and Self-Activation of Orphan GPR52. *Nature* **2020**, *579*, 152–157.

111. Shimamura, T.; Shiroishi, M.; Weyand, S.; Tsujimoto, H.; Winter, G.; Katritch, V.; Abagyan, R.; Cherezov, V.; Liu, W.; Han, G.W. Structure of the Human Histamine H 1 Receptor Complex with Doxepin. *Nature* **2011**, *475*, 65–70.

112. Chrencik, J.E.; Roth, C.B.; Terakado, M.; Kurata, H.; Omi, R.; Kihara, Y.; Warshaviak, D.; Nakade, S.; Asmar-Rovira, G.; Mileni, M. Crystal Structure of Antagonist Bound Human Lysophosphatidic Acid Receptor 1. *Cell* **2015**, *161*, 1633–1643.

113. Stauch, B.; Johansson, L.C.; McCorvy, J.D.; Patel, N.; Han, G.W.; Huang, X.-P.; Gati, C.; Batyuk, A.; Slocum, S.T.; Ishchenko, A. Structural Basis of Ligand Recognition at the Human MT1 Melatonin Receptor. *Nature* **2019**, *569*, 284–288.

114. Ishchenko, A.; Stauch, B.; Han, G.W.; Batyuk, A.; Shiriaeva, A.; Li, C.; Zatsepin, N.; Weierstall, U.; Liu, W.; Nango, E. Toward G Protein-Coupled Receptor Structure-Based Drug Design Using X-Ray Lasers. *IUCrJ* **2019**, *6*, 1106–1119.

115. Schöppe, J.; Ehrenmann, J.; Klenk, C.; Rucktooa, P.; Schütz, M.; Doré, A.S.; Plückthun, A. Crystal Structures of the Human Neurokinin 1 Receptor in Complex with Clinically Used Antagonists. *Nat Commun* **2019**, *10*, 17.

116. Yin, J.; Chapman, K.; Clark, L.D.; Shao, Z.; Borek, D.; Xu, Q.; Wang, J.; Rosenbaum, D.M. Crystal Structure of the Human NK1 Tachykinin Receptor. *Proceedings of the National Academy of Sciences* **2018**, *115*, 13264–13269.

117. Chen, S.; Lu, M.; Liu, D.; Yang, L.; Yi, C.; Ma, L.; Zhang, H.; Liu, Q.; Frimurer, T.M.; Wang, M.-W. Human Substance P Receptor Binding Mode of the Antagonist Drug Aprepitant by NMR and Crystallography. *Nat Commun* **2019**, *10*, 638.

118. Yang, Z.; Han, S.; Keller, M.; Kaiser, A.; Bender, B.J.; Bosse, M.; Burkert, K.; Kögler, L.M.; Wifling, D.; Bernhardt, G. Structural Basis of Ligand Binding Modes at the Neuropeptide Y Y1 Receptor. *Nature* **2018**, *556*, 520–524.

119. Egloff, P.; Hillenbrand, M.; Klenk, C.; Batyuk, A.; Heine, P.; Balada, S.; Schlinkmann, K.M.; Scott, D.J.; Schütz, M.; Plückthun, A. Structure of Signaling-Competent Neurotensin Receptor 1 Obtained by Directed Evolution in Escherichia Coli. *Proc Natl Acad Sci U S A* **2014**, *111*, doi:10.1073/pnas.1317903111.

120. White, J.F.; Noinaj, N.; Shibata, Y.; Love, J.; Kloss, B.; Xu, F.; Gvozdenovic-Jeremic, J.; Shah, P.; Shiloach, J.; Tate, C.G.; et al. Structure of the Agonist-Bound Neurotensin Receptor. *Nature* **2012**, *490*, 508–513, doi:10.1038/nature11558.

121. Krumm, B.E.; White, J.F.; Shah, P.; Grisshammer, R. Structural Prerequisites for G-Protein Activation by the Neurotensin Receptor. *Nat Commun* **2015**, *6*, doi:10.1038/ncomms8895.

122. Krumm, B.E.; Lee, S.; Bhattacharya, S.; Botos, I.; White, C.F.; Du, H.; Vaidehi, N.; Grisshammer, R. Structure and Dynamics of a Constitutively Active Neurotensin Receptor. *Sci Rep* **2016**, *6*, doi:10.1038/srep38564.

123. Granier, S.; Manglik, A.; Kruse, A.C.; Kobilka, T.S.; Thian, F.S.; Weis, W.I.; Kobilka, B.K. Structure of the δ-Opioid Receptor Bound to Naltrindole. *Nature* **2012**, *485*, 400–404.

124. Fenalti, G.; Giguere, P.M.; Katritch, V.; Huang, X.-P.; Thompson, A.A.; Cherezov, V.; Roth, B.L.; Stevens, R.C. Molecular Control of δ-Opioid Receptor Signalling. *Nature* **2014**, *506*, 191–196.

125. Fenalti, G.; Zatsepin, N.A.; Betti, C.; Giguere, P.; Han, G.W.; Ishchenko, A.; Liu, W.; Guillemyn, K.; Zhang, H.; James, D.; et al. Structural Basis for Bifunctional Peptide Recognition at Human δ-Opioid Receptor. *Nat Struct Mol Biol* **2015**, *22*, 265–268, doi:10.1038/nsmb.2965.

126. Wu, H.; Wacker, D.; Mileni, M.; Katritch, V.; Han, G.W.; Vardy, E.; Liu, W.; Thompson, A.A.; Huang, X.-P.; Carroll, F.I. Structure of the Human κ-Opioid Receptor in Complex with JDTic. *Nature* **2012**, *485*, 327–332.

127. Che, T.; Majumdar, S.; Zaidi, S.A.; Ondachi, P.; McCorvy, J.D.; Wang, S.; Mosier, P.D.; Uprety, R.; Vardy, E.; Krumm, B.E. Structure of the Nanobody-Stabilized Active State of the Kappa Opioid Receptor. *Cell* **2018**, *172*, 55–67.

128. Huang, W.; Manglik, A.; Venkatakrishnan, A.J.; Laeremans, T.; Feinberg, E.N.; Sanborn, A.L.; Kato, H.E.; Livingston, K.E.; Thorsen, T.S.; Kling, R.C. Structural Insights into M-Opioid Receptor Activation. *Nature* **2015**, *524*, 315–321.

129. Manglik, A.; Kruse, A.C.; Kobilka, T.S.; Thian, F.S.; Mathiesen, J.M.; Sunahara, R.K.; Pardo, L.; Weis, W.I.; Kobilka, B.K.; Granier, S. Crystal Structure of the M-Opioid Receptor Bound to a Morphinan Antagonist. *Nature* **2012**, *485*, 321–326.

130. Koehl, A.; Hu, H.; Maeda, S.; Zhang, Y.; Qu, Q.; Paggi, J.M.; Latorraca, N.R.; Hilger, D.; Dawson, R.; Matile, H.; et al. Structure of the μ-Opioid Receptor-G<inf>i</Inf> Protein Complex. *Nature* **2018**, *558*, 547–552, doi:10.1038/s41586-018-0219-7.

131. Miller, R.L.; Thompson, A.A.; Trapella, C.; Guerrini, R.; Malfacini, D.; Patel, N.; Han, G.W.; Cherezov, V.; Caló, G.; Katritch, V. The Importance of Ligand-Receptor Conformational Pairs in Stabilization: Spotlight on the N/OFQ G Protein-Coupled Receptor. *Structure* **2015**, *23*, 2291–2299.

132. Thompson, A.A.; Liu, W.; Chun, E.; Katritch, V.; Wu, H.; Vardy, E.; Huang, X.-P.; Trapella, C.; Guerrini, R.; Calo, G. Structure of the Nociceptin/Orphanin FQ Receptor in Complex with a Peptide Mimetic. *Nature* **2012**, *485*, 395.

133. Shimamura, T.; Hiraki, K.; Takahashi, N.; Hori, T.; Ago, H.; Masuda, K.; Takio, K.; Ishiguro, M.; Miyano, M. Crystal Structure of Squid Rhodopsin with Intracellularly Extended Cytoplasmic Region. *Journal of Biological Chemistry* **2008**, *283*, 17753–17756, doi:10.1074/jbc.C800040200.

134. Rappas, M.; Ali, A.A.E.; Bennett, K.A.; Brown, J.D.; Bucknell, S.J.; Congreve, M.; Cooke, R.M.; Cseke, G.; de Graaf, C.; Doré, A.S. Comparison of Orexin 1 and Orexin 2 Ligand Binding Modes Using X-Ray Crystallography and Computational Analysis. *J Med Chem* **2019**, *63*, 1528–1543.

135. Yin, J.; Babaoglu, K.; Brautigam, C.A.; Clark, L.; Shao, Z.; Scheuermann, T.H.; Harrell, C.M.; Gotter, A.L.; Roecker, A.J.; Winrow, C.J. Structure and Ligand-Binding Mechanism of the Human OX1 and OX2 Orexin Receptors. *Nat Struct Mol Biol* **2016**, *23*, 293–299.

136. Suno, R.; Kimura, K.T.; Nakane, T.; Yamashita, K.; Wang, J.; Fujiwara, T.; Yamanaka, Y.; Im, D.; Horita, S.; Tsujimoto, H. Crystal Structures of Human Orexin 2 Receptor Bound to the Subtype-Selective Antagonist EMPA. *Structure* **2018**, *26*, 7–19.

137. Yin, J.; Mobarec, J.C.; Kolb, P.; Rosenbaum, D.M. Crystal Structure of the Human OX2 Orexin Receptor Bound to the Insomnia Drug Suvorexant. *Nature* **2015**, *519*, 247–250.

138. Cheng, R.K.Y.; Fiez-Vandal, C.; Schlenker, O.; Edman, K.; Aggeler, B.; Brown, D.G.; Brown, G.A.; Cooke, R.M.; Dumelin, C.E.; Doré, A.S.; et al. Structural Insight into Allosteric Modulation of Protease-Activated Receptor 2. *Nature* **2017**, *545*, 112–115, doi:10.1038/nature22309.

139. Zhang, D.; Gao, Z.-G.; Zhang, K.; Kiselev, E.; Crane, S.; Wang, J.; Paoletta, S.; Yi, C.; Ma, L.; Zhang, W. Two Disparate Ligand-Binding Sites in the Human P2Y1 Receptor. *Nature* **2015**, *520*, 317–321.

140. Zhang, J.; Zhang, K.; Gao, Z.-G.; Paoletta, S.; Zhang, D.; Han, G.W.; Li, T.; Ma, L.; Zhang, W.; Müller, C.E. Agonist-Bound Structure of the Human P2Y12 Receptor. *Nature* **2014**, *509*, 119–122.

141. Zhang, K.; Zhang, J.; Gao, Z.-G.; Zhang, D.; Zhu, L.; Han, G.W.; Moss, S.M.; Paoletta, S.; Kiselev, E.; Lu, W. Structure of the Human P2Y12 Receptor in Complex with an Antithrombotic Drug. *Nature* **2014**, *509*, 115–118.

142. Zhang, C.; Srinivasan, Y.; Arlow, D.H.; Fung, J.J.; Palmer, D.; Zheng, Y.; Green, H.F.; Pandey, A.; Dror, R.O.; Shaw, D.E. High-Resolution Crystal Structure of Human Protease-Activated Receptor 1. *Nature* **2012**, *492*, 387.

143. Cheng, R.K.Y.; Fiez-Vandal, C.; Schlenker, O.; Edman, K.; Aggeler, B.; Brown, D.G.; Brown, G.A.; Cooke, R.M.; Dumelin, C.E.; Doré, A.S. Structural Insight into Allosteric Modulation of Protease-Activated Receptor 2. *Nature* **2017**, *545*, 112–115.

144. Wang, L.; Yao, D.; Deepak, R.N.V.K.; Liu, H.; Xiao, Q.; Fan, H.; Gong, W.; Wei, Z.; Zhang, C. Structures of the Human PGD2 Receptor CRTH2 Reveal Novel Mechanisms for Ligand Recognition. *Mol Cell* **2018**, *72*, 48-59. e4.

145. Audet, M.; White, K.L.; Breton, B.; Zarzycka, B.; Han, G.W.; Lu, Y.; Gati, C.; Batyuk, A.; Popov, P.; Velasquez, J. Crystal Structure of Misoprostol Bound to the Labor Inducer Prostaglandin E2 Receptor. *Nat Chem Biol* **2019**, *15*, 11–17.

146. Morimoto, K.; Suno, R.; Hotta, Y.; Yamashita, K.; Hirata, K.; Yamamoto, M.; Narumiya, S.; Iwata, S.; Kobayashi, T. Crystal Structure of the Endogenous Agonist-Bound Prostanoid Receptor EP3. *Nat Chem Biol* **2019**, *15*, 8–10.

147. Toyoda, Y.; Morimoto, K.; Suno, R.; Horita, S.; Yamashita, K.; Hirata, K.; Sekiguchi, Y.; Yasuda, S.; Shiroishi, M.; Shimizu, T. Ligand Binding to Human Prostaglandin E Receptor EP4 at the Lipid-Bilayer Interface. *Nat Chem Biol* **2019**, *15*, 18–26.

148. Cao, C.; Tan, Q.; Xu, C.; He, L.; Yang, L.; Zhou, Y.; Zhou, Y.; Qiao, A.; Lu, M.; Yi, C. Structural Basis for Signal Recognition and Transduction by Platelet-Activating-Factor Receptor. *Nat Struct Mol Biol* **2018**, *25*, 488–495.

149. Hori, T.; Okuno, T.; Hirata, K.; Yamashita, K.; Kawano, Y.; Yamamoto, M.; Hato, M.; Nakamura, M.; Shimizu, T.; Yokomizo, T. Na+-Mimicking Ligands Stabilize the Inactive State of Leukotriene B4 Receptor BLT1. *Nat Chem Biol* **2018**, *14*, 262–269.

150. Hanson, M.A.; Roth, C.B.; Jo, E.; Griffith, M.T.; Scott, F.L.; Reinhart, G.; Desale, H.; Clemons, B.; Cahalan, S.M.; Schuerer, S.C. Crystal Structure of a Lipid G Protein–Coupled Receptor. *Science (1979)* **2012**, *335*, 851–855.

151. Haffke, M.; Fehlmann, D.; Rummel, G.; Boivineau, J.; Duckely, M.; Gommermann, N.; Cotesta, S.; Sirockin, F.; Freuler, F.; Littlewood-Evans, A. Structural Basis of Species-Selective Antagonist Binding to the Succinate Receptor. *Nature* **2019**, *574*, 581–585.

152. Fan, H.; Chen, S.; Yuan, X.; Han, S.; Zhang, H.; Xia, W.; Xu, Y.; Zhao, Q.; Wu, B. Structural Basis for Ligand Recognition of the Human Thromboxane A2 Receptor. *Nat Chem Biol* **2019**, *15*, 27–33.

153. Burg, J.S.; Ingram, J.R.; Venkatakrishnan, A.J.; Jude, K.M.; Dukkipati, A.; Feinberg, E.N.; Angelini, A.; Waghray, D.; Dror, R.O.; Ploegh, H.L.; et al. Structural Basis for Chemokine Recognition and Activation of a Viral G Protein-Coupled Receptor. *Science (1979)* **2015**, *347*, 1113–1117, doi:10.1126/science.aaa5026.

154. Miles, T.F.; Spiess, K.; Jude, K.M.; Tsutsumi, N.; Burg, J.S.; Ingram, J.R.; Waghray, D.; Hjorto, G.M.; Larsen, O.; Ploegh, H.L.; et al. Viral GPCR US28 Can Signal in Response to Chemokine Agonists of Nearly Unlimited Structural Degeneracy. *Elife* **2018**, *7*, doi:10.7554/eLife.35850.

155. Su, M.; Zhu, L.; Zhang, Y.; Paknejad, N.; Dey, R.; Huang, J.; Lee, M.-Y.; Williams, D.; Jordan, K.D.; Eng, E.T. Structural Basis of the Activation of Heterotrimeric Gs-Protein by Isoproterenol-Bound Β1-Adrenergic Receptor. *Mol Cell* **2020**, *80*, 59–71.

156. Gusach, A.; Luginina, A.; Marin, E.; Brouillette, R.L.; Besserer-Offroy, É.; Longpré, J.M.; Ishchenko, A.; Popov, P.; Patel, N.; Fujimoto, T.; et al. Structural Basis of Ligand Selectivity and Disease Mutations in Cysteinyl Leukotriene Receptors. *Nat Commun* **2019**, *10*, doi:10.1038/s41467-019-13348-2.

157. Kooistra, A.J.; Mordalski, S.; Pándy-Szekeres, G.; Esguerra, M.; Mamyrbekov, A.; Munk, C.; Keserű, G.M.; Gloriam, D.E. GPCRdb in 2021: Integrating GPCR Sequence, Structure and Function. *Nucleic Acids Res* **2021**, *49*, D335–D343.

158. Xu, P.; Huang, S.; Zhang, H.; Mao, C.; Zhou, X.E.; Cheng, X.; Simon, I.A.; Shen, D.-D.; Yen, H.-Y.; Robinson, C. V Structural Insights into the Lipid and Ligand Regulation of Serotonin Receptors. *Nature* **2021**, *592*, 469–473.

159. Miyagi, H.; Asada, H.; Suzuki, M.; Takahashi, Y.; Yasunaga, M.; Suno, C.; Iwata, S.; Saito, J. The Discovery of a New Antibody for BRIL-Fused GPCR Structure Determination. *Sci Rep* **2020**, *10*, 11669.

160. Huang, S.; Xu, P.; Tan, Y.; You, C.; Zhang, Y.; Jiang, Y.; Xu, H.E. Structural Basis for Recognition of Anti-Migraine Drug Lasmiditan by the Serotonin Receptor 5-HT1F–G Protein Complex. *Cell Res* **2021**, *31*, 1036–1038.

161. Kim, K.; Che, T.; Panova, O.; DiBerto, J.F.; Lyu, J.; Krumm, B.E.; Wacker, D.; Robertson, M.J.; Seven, A.B.; Nichols, D.E. Structure of a Hallucinogen-Activated Gq-Coupled 5-HT2A Serotonin Receptor. *Cell* **2020**, *182*, 1574–1588.

162. Chen, Z.; Fan, L.; Wang, H.; Yu, J.; Lu, D.; Qi, J.; Nie, F.; Luo, Z.; Liu, Z.; Cheng, J. Structure-Based Design of a Novel Third-Generation Antipsychotic Drug Lead with Potential Antidepressant Properties. *Nat Neurosci* **2022**, *25*, 39–49.

163. Draper-Joyce, C.J.; Bhola, R.; Wang, J.; Bhattarai, A.; Nguyen, A.T.N.; O'Sullivan, K.; Chia, L.Y.; Venugopal, H.; Valant, C.; Thal, D.M. Positive Allosteric Mechanisms of Adenosine A1 Receptor-Mediated Analgesia. *Nature* **2021**, *597*, 571–576.

164. Jespers, W.; Verdon, G.; Azuaje, J.; Majellaro, M.; Keränen, H.; García-Mera, X.; Congreve, M.; Deflorian, F.; De Graaf, C.; Zhukov, A. X-ray Crystallography and Free Energy Calculations Reveal the Binding Mechanism of A2A Adenosine Receptor Antagonists. *Angewandte Chemie International Edition* **2020**, *59*, 16536–16543.

165. Amelia, T.; van Veldhoven, J.P.D.; Falsini, M.; Liu, R.; Heitman, L.H.; van Westen, G.J.P.; Segala, E.; Verdon, G.; Cheng, R.K.Y.; Cooke, R.M. Crystal Structure and Subsequent Ligand Design of a Nonriboside Partial Agonist Bound to the Adenosine A2A Receptor. *J Med Chem* **2021**, *64*, 3827–3842.

166. Shimazu, Y.; Tono, K.; Tanaka, T.; Yamanaka, Y.; Nakane, T.; Mori, C.; Terakado Kimura, K.; Fujiwara, T.; Sugahara, M.; Tanaka, R. High-Viscosity Sample-Injection Device for Serial Femtosecond Crystallography at Atmospheric Pressure. *J Appl Crystallogr* **2019**, *52*, 1280–1288.

167. Ihara, K.; Hato, M.; Nakane, T.; Yamashita, K.; Kimura-Someya, T.; Hosaka, T.; Ishizuka-Katsura, Y.; Tanaka, R.; Tanaka, T.; Sugahara, M. Isoprenoid-Chained Lipid EROCOC17+ 4: A New Matrix for Membrane Protein Crystallization and a Crystal Delivery Medium in Serial Femtosecond Crystallography. *Sci Rep* **2020**, *10*, 19305.

168. Martin-Garcia, J.M.; Zhu, L.; Mendez, D.; Lee, M.-Y.; Chun, E.; Li, C.; Hu, H.; Subramanian, G.; Kissick, D.; Ogata, C. High-Viscosity Injector-Based Pink-Beam Serial Crystallography of Microcrystals at a Synchrotron Radiation Source. *IUCrJ* **2019**, *6*, 412–425.

169. Nass, K.; Cheng, R.; Vera, L.; Mozzanica, A.; Redford, S.; Ozerov, D.; Basu, S.; James, D.; Knopp, G.; Cirelli, C. Advances in Long-Wavelength Native Phasing at X-Ray Free-Electron Lasers. *IUCrJ* **2020**, *7*, 965–975.

170. Lee, M.-Y.; Geiger, J.; Ishchenko, A.; Han, G.W.; Barty, A.; White, T.A.; Gati, C.; Batyuk, A.; Hunter, M.S.; Aquila, A. Harnessing the Power of an X-Ray Laser for Serial Crystallography of Membrane Proteins Crystallized in Lipidic Cubic Phase. *IUCrJ* **2020**, *7*, 976–984.

171. Martynowycz, M.W.; Shiriaeva, A.; Ge, X.; Hattne, J.; Nannenga, B.L.; Cherezov, V.; Gonen, T. MicroED Structure of the Human Adenosine Receptor Determined from a Single Nanocrystal in LCP. *Proceedings of the National Academy of Sciences* **2021**, *118*, e2106041118.

172. Borodovsky, A.; Barbon, C.M.; Wang, Y.; Ye, M.; Prickett, L.; Chandra, D.; Shaw, J.; Deng, N.; Sachsenmeier, K.; Clarke, J.D. Small Molecule AZD4635 Inhibitor of A2AR Signaling Rescues Immune Cell Function Including CD103+ Dendritic Cells Enhancing Anti-Tumor Immunity. *J Immunother Cancer* **2020**, *8*.

173. Brown, A.J.H.; Bradley, S.J.; Marshall, F.H.; Brown, G.A.; Bennett, K.A.; Brown, J.; Cansfield, J.E.; Cross, D.M.; de Graaf, C.; Hudson, B.D. From Structure to Clinic: Design of a Muscarinic M1 Receptor Agonist with the Potential to Treat Alzheimer's Disease. *Cell* **2021**, *184*, 5886–5901.

174. Staus, D.P.; Hu, H.; Robertson, M.J.; Kleinhenz, A.L.W.; Wingler, L.M.; Capel, W.D.; Latorraca, N.R.; Lefkowitz, R.J.; Skiniotis, G. Structure of the M2 Muscarinic Receptor–β-Arrestin Complex in a Lipid Nanodisc. *Nature* **2020**, *579*, 297–302.

175. Qu, L.; Zhou, Q.T.; Wu, D.; Zhao, S.W. Crystal Structures of the Alpha2A Adrenergic Receptor in Complex with an Antagonist RSC. *Released Protein Data Bank* **2019**.

176. Yuan, D.; Liu, Z.; Kaindl, J.; Maeda, S.; Zhao, J.; Sun, X.; Xu, J.; Gmeiner, P.; Wang, H.-W.; Kobilka, B.K. Activation of the A2B Adrenoceptor by the Sedative Sympatholytic Dexmedetomidine. *Nat Chem Biol* **2020**, *16*, 507–512.

177. Chen, X.Y.; Wu, D.; Wu, L.J.; Han, G.W.; Guo, Y.; Zhong, G.S. Crystal Structure of Human Alpha2C Adrenergic G Protein-Coupled Receptor. *Released Protein Data Bank* **2019**.

178. Xu, X.; Kaindl, J.; Clark, M.J.; Hübner, H.; Hirata, K.; Sunahara, R.K.; Gmeiner, P.; Kobilka, B.K.; Liu, X. Binding Pathway Determines Norepinephrine Selectivity for the Human B1AR over B2AR. *Cell Res* **2021**, *31*, 569–579.

179. Liu, X.; Xu, X.; Hilger, D.; Aschauer, P.; Tiemann, J.K.S.; Du, Y.; Liu, H.; Hirata, K.; Sun, X.; Guixà-González, R. Structural Insights into the Process of GPCR-G Protein Complex Formation. *Cell* **2019**, *177*, 1243–1251.

180. Nguyen, A.H.; Thomsen, A.R.B.; Cahill III, T.J.; Huang, R.; Huang, L.-Y.; Marcink, T.; Clarke, O.B.; Heissel, S.; Masoudi, A.; Ben-Hail, D. Structure of an Endosomal Signaling GPCR–G Protein–β-Arrestin Megacomplex. *Nat Struct Mol Biol* **2019**, *26*, 1123–1131.

181. Yang, F.; Ling, S.; Zhou, Y.; Zhang, Y.; Lv, P.; Liu, S.; Fang, W.; Sun, W.; Hu, L.A.; Zhang, L. Different Conformational Responses of the B2-Adrenergic Receptor-Gs Complex upon Binding of the Partial Agonist Salbutamol or the Full Agonist Isoprenaline. *Natl Sci Rev* **2021**, *8*, nwaa284.

182. Zhang, Y.; Yang, F.; Ling, S.; Lv, P.; Zhou, Y.; Fang, W.; Sun, W.; Zhang, L.; Shi, P.; Tian, C. Single-Particle Cryo-EM Structural Studies of the B2AR–Gs Complex Bound with a Full Agonist Formoterol. *Cell Discov* **2020**, *6*, 45.

183. Nagiri, C.; Kobayashi, K.; Tomita, A.; Kato, M.; Kobayashi, K.; Yamashita, K.; Nishizawa, T.; Inoue, A.; Shihoya, W.; Nureki, O. Cryo-EM Structure of the Β3-Adrenergic Receptor Reveals the Molecular Basis of Subtype Selectivity. *Mol Cell* **2021**, *81*, 3205–3215.

184. Zhang, X.; He, C.; Wang, M.; Zhou, Q.; Yang, D.; Zhu, Y.; Feng, W.; Zhang, H.; Dai, A.; Chu, X. Structures of the Human Cholecystokinin Receptors Bound to Agonists and Antagonists. *Nat Chem Biol* **2021**, *17*, 1230–1237.

185. Wang, X.; Liu, D.; Shen, L.; Li, F.; Li, Y.; Yang, L.; Xu, T.; Tao, H.; Yao, D.; Wu, L. A Genetically Encoded F-19 NMR Probe Reveals the Allosteric Modulation Mechanism of Cannabinoid Receptor 1. *J Am Chem Soc* **2021**, *143*, 16320–16325.

186. Zhuang, Y.; Xu, P.; Mao, C.; Wang, L.; Krumm, B.; Zhou, X.E.; Huang, S.; Liu, H.; Cheng, X.; Huang, X.-P. Structural Insights into the Human D1 and D2 Dopamine Receptor Signaling Complexes. *Cell* **2021**, *184*, 931–942.

187. Xiao, P.; Yan, W.; Gou, L.; Zhong, Y.-N.; Kong, L.; Wu, C.; Wen, X.; Yuan, Y.; Cao, S.; Qu, C. Ligand Recognition and Allosteric Regulation of DRD1-Gs Signaling Complexes. *Cell* **2021**, *184*, 943–956.

188. Sun, B.; Feng, D.; Chu, M.L.-H.; Fish, I.; Lovera, S.; Sands, Z.A.; Kelm, S.; Valade, A.; Wood, M.; Ceska, T. Crystal Structure of Dopamine D1 Receptor in Complex with G Protein and a Non-Catechol Agonist. *Nat Commun* **2021**, *12*, 3305.

189. Yin, J.; Chen, K.-Y.M.; Clark, M.J.; Hijazi, M.; Kumari, P.; Bai, X.; Sunahara, R.K.; Barth, P.; Rosenbaum, D.M. Structure of a D2 Dopamine Receptor–G-Protein Complex in a Lipid Membrane. *Nature* **2020**, *584*, 125–129.

190. Im, D.; Inoue, A.; Fujiwara, T.; Nakane, T.; Yamanaka, Y.; Uemura, T.; Mori, C.; Shiimura, Y.; Kimura, K.T.; Asada, H. Structure of the Dopamine D2 Receptor in Complex with the Antipsychotic Drug Spiperone. *Nat Commun* **2020**, *11*, 6442.

191. Xu, P.; Huang, S.; Mao, C.; Krumm, B.E.; Zhou, X.E.; Tan, Y.; Huang, X.-P.; Liu, Y.; Shen, D.-D.; Jiang, Y. Structures of the Human Dopamine D3 Receptor-Gi Complexes. *Mol Cell* **2021**, *81*, 1147–1159.

192. Zhou, Y.; Cao, C.; He, L.; Wang, X.; Zhang, X.C. Crystal Structure of Dopamine Receptor D4 Bound to the Subtype Selective Ligand, L745870. *Elife* **2019**, *8*, e48822.

193. Shiimura, Y.; Horita, S.; Hamamoto, A.; Asada, H.; Hirata, K.; Tanaka, M.; Mori, K.; Uemura, T.; Kobayashi, T.; Iwata, S. Structure of an Antagonist-Bound Ghrelin Receptor Reveals Possible Ghrelin Recognition Mode. *Nat Commun* **2020**, *11*, 4160.

194. Liu, H.; Sun, D.; Myasnikov, A.; Damian, M.; Baneres, J.-L.; Sun, J.; Zhang, C. Structural Basis of Human Ghrelin Receptor Signaling by Ghrelin and the Synthetic Agonist Ibutamoren. *Nat Commun* **2021**, *12*, 6410.

195. Yan, W.; Cheng, L.; Wang, W.; Wu, C.; Yang, X.; Du, X.; Ma, L.; Qi, S.; Wei, Y.; Lu, Z. Structure of the Human Gonadotropin-Releasing Hormone Receptor GnRH1R Reveals an Unusual Ligand Binding Mode. *Nat Commun* **2020**, *11*, 5287.

196. Zhou, Y.; Daver, H.; Trapkov, B.; Wu, L.; Wu, M.; Harpsøe, K.; Gentry, P.R.; Liu, K.; Larionova, M.; Liu, J. Molecular Insights into Ligand Recognition and G Protein Coupling of the Neuromodulatory Orphan Receptor GPR139. *Cell Res* **2022**, *32*, 210–213.

197. Yang, F.; Mao, C.; Guo, L.; Lin, J.; Ming, Q.; Xiao, P.; Wu, X.; Shen, Q.; Guo, S.; Shen, D.-D. Structural Basis of GPBAR Activation and Bile Acid Recognition. *Nature* **2020**, *587*, 499–504.

198. Xia, R.; Wang, N.; Xu, Z.; Lu, Y.; Song, J.; Zhang, A.; Guo, C.; He, Y. Cryo-EM Structure of the Human Histamine H1 Receptor/Gq Complex. *Nat Commun* **2021**, *12*, 2086.

199. Michaelian, N.; Sadybekov, A.; Besserer-Offroy, É.; Han, G.W.; Krishnamurthy, H.; Zamlynny, B.A.; Fradera, X.; Siliphaivanh, P.; Presland, J.; Spencer, K.B. Structural Insights on Ligand Recognition at the Human Leukotriene B4 Receptor 1. *Nat Commun* **2021**, *12*, 2971.

200. Zhang, H.; Chen, L.-N.; Yang, D.; Mao, C.; Shen, Q.; Feng, W.; Shen, D.-D.; Dai, A.; Xie, S.; Zhou, Y. Structural Insights into Ligand Recognition and Activation of the Melanocortin-4 Receptor. *Cell Res* **2021**, *31*, 1163–1175.

201. Cao, C.; Kang, H.J.; Singh, I.; Chen, H.; Zhang, C.; Ye, W.; Hayes, B.W.; Liu, J.; Gumpper, R.H.; Bender, B.J. Structure, Function and Pharmacology of Human Itch GPCRs. *Nature* **2021**, *600*, 170–175.

202. Okamoto, H.H.; Miyauchi, H.; Inoue, A.; Raimondi, F.; Tsujimoto, H.; Kusakizako, T.; Shihoya, W.; Yamashita, K.; Suno, R.; Nomura, N. Cryo-EM Structure of the Human MT1–Gi Signaling Complex. *Nat Struct Mol Biol* **2021**, *28*, 694–701.

203. Tang, T.; Hartig, C.; Chen, Q.; Zhao, W.; Kaiser, A.; Zhang, X.; Zhang, H.; Qu, H.; Yi, C.; Ma, L. Structural Basis for Ligand Recognition of the Neuropeptide Y Y2 Receptor. *Nat Commun* **2021**, *12*, 737.

204. Deluigi, M.; Klipp, A.; Klenk, C.; Merklinger, L.; Eberle, S.A.; Morstein, L.; Heine, P.; Mittl, P.R.E.; Ernst, P.; Kamenecka, T.M. Complexes of the Neurotensin Receptor 1 with Small-Molecule Ligands Reveal Structural Determinants of Full, Partial, and Inverse Agonism. *Sci Adv* **2021**, *7*, eabe5504.

205. Claff, T.; Yu, J.; Blais, V.; Patel, N.; Martin, C.; Wu, L.; Han, G.W.; Holleran, B.J.; Van der Poorten, O.; White, K.L. Elucidating the Active δ-Opioid Receptor Crystal Structure with Peptide and Small-Molecule Agonists. *Sci Adv* **2019**, *5*, eaax9115.

206. Che, T.; English, J.; Krumm, B.E.; Kim, K.; Pardon, E.; Olsen, R.H.J.; Wang, S.; Zhang, S.; Diberto, J.F.; Sciaky, N. Nanobody-Enabled Monitoring of Kappa Opioid Receptor States. *Nat Commun* **2020**, *11*, 1145.

207. Hellmann, J.; Drabek, M.; Yin, J.; Gunera, J.; Pröll, T.; Kraus, F.; Langmead, C.J.; Hübner, H.; Weikert, D.; Kolb, P. Structure-Based Development of a Subtype-Selective Orexin 1 Receptor Antagonist. *Proceedings of the National Academy of Sciences* **2020**, *117*, 18059–18067.

208. Hong, C.; Byrne, N.J.; Zamlynny, B.; Tummala, S.; Xiao, L.; Shipman, J.M.; Partridge, A.T.; Minnick, C.; Breslin, M.J.; Rudd, M.T. Structures of Active-State Orexin Receptor 2 Rationalize Peptide and Small-Molecule Agonist Recognition and Receptor Activation. *Nat Commun* **2021**, *12*, 815.

209. Waltenspühl, Y.; Schöppe, J.; Ehrenmann, J.; Kummer, L.; Plückthun, A. Crystal Structure of the Human Oxytocin Receptor. *Sci Adv* **2020**, *6*, eabb5419.

210. Liu, H.; Deepak, R.N.V.K.; Shiriaeva, A.; Gati, C.; Batyuk, A.; Hu, H.; Weierstall, U.; Liu, W.; Wang, L.; Cherezov, V. Molecular Basis for Lipid Recognition by the Prostaglandin D2 Receptor CRTH2. *Proceedings of the National Academy of Sciences* **2021**, *118*, e2102813118.

211. Qu, C.; Mao, C.; Xiao, P.; Shen, Q.; Zhong, Y.-N.; Yang, F.; Shen, D.-D.; Tao, X.; Zhang, H.; Yan, X. Ligand Recognition, Unconventional Activation, and G Protein Coupling of the Prostaglandin E2 Receptor EP2 Subtype. *Sci Adv* **2021**, *7*, eabf1268.

212. Nojima, S.; Fujita, Y.; Kimura, K.T.; Nomura, N.; Suno, R.; Morimoto, K.; Yamamoto, M.; Noda, T.; Iwata, S.; Shigematsu, H. Cryo-EM Structure of the Prostaglandin E Receptor EP4 Coupled to G Protein. *Structure* **2021**, *29*, 252–260.

213. Xu, Z.; Ikuta, T.; Kawakami, K.; Kise, R.; Qian, Y.; Xia, R.; Sun, M.-X.; Zhang, A.; Guo, C.; Cai, X.-H. Structural Basis of Sphingosine-1-Phosphate Receptor 1 Activation and Biased Agonism. *Nat Chem Biol* **2022**, *18*, 281–288.

214. Zhao, C.; Cheng, L.; Wang, W.; Wang, H.; Luo, Y.; Feng, Y.; Wang, X.; Fu, H.; Cai, Y.; Yang, S. Structural Insights into Sphingosine-1-Phosphate Recognition and Ligand Selectivity of S1PR3–Gi Signaling Complexes. *Cell Res* **2022**, *32*, 218–221.

215. Maeda, S.; Shiimura, Y.; Asada, H.; Hirata, K.; Luo, F.; Nango, E.; Tanaka, N.; Toyomoto, M.; Inoue, A.; Aoki, J. Endogenous Agonist–Bound S1PR3 Structure Reveals Determinants of G Protein–Subtype Bias. *Sci Adv* **2021**, *7*, eabf5325.

216. Yuan, Y.; Jia, G.; Wu, C.; Wang, W.; Cheng, L.; Li, Q.; Li, Z.; Luo, K.; Yang, S.; Yan, W. Structures of Signaling Complexes of Lipid Receptors S1PR1 and S1PR5 Reveal Mechanisms of Activation and Drug Recognition. *Cell Res* **2021**, *31*, 1263–1274.

217. Velcicky, J.; Wilcken, R.; Cotesta, S.; Janser, P.; Schlapbach, A.; Wagner, T.; Piechon, P.; Villard, F.; Bouhelal, R.; Piller, F. Discovery and Optimization of Novel SUCNR1 Inhibitors: Design of Zwitterionic Derivatives with a Salt Bridge for the Improvement of Oral Exposure. *J Med Chem* **2020**, *63*, 9856–9875.

218. Harding, S.D.; Armstrong, J.F.; Faccenda, E.; Southan, C.; Alexander, S.P.H.; Davenport, A.P.; Pawson, A.J.; Spedding, M.; Davies, J.A.; NC-IUPHAR The IUPHAR/BPS Guide to PHARMACOLOGY in 2022: Curating Pharmacology for COVID-19, Malaria and Antibacterials. *Nucleic Acids Res* **2022**, *50*, D1282–D1294.
