# Supplementary material for: G Protein-Coupled Receptor–Ligand Pose and Functional Class Prediction
Source: Int J Mol Sci. 2024 Jun 22;25(13):6876. doi: 10.3390/ijms25136876 (PMC11241240; doi:10.3390/ijms25136876)
Supplement: Supplementary file 1 [file ijms-25-06876-s001.zip › Szwabowski_IJMS_2024_Supplemental_TablesS6_to_S12.pdf]

GPCR Ligand Pose and Functional Class Prediction

Gregory L. Szwabowski [1], Makenzie Griffing [2], Elijah J. Mugabe [3], Daniel O'Malley[4], Lindsey N. Baker[5], Daniel L. Baker [6,*], and Abby L. Parrill [7,*]

Department of Chemistry, University of Memphis, Memphis TN 38152, USA

1 gszwabowski@gmail.com

2 makenziegriffing@outlook.com

3 elm4026@med.cornell.edu

4 domalley@memphis.edu

5 lindsey.baker@yale.edu

6 dlbaker@memphis.edu

7 aparrill@memphis.edu

* Correspondence: aparrill@memphis.edu; Tel.: 1-901-678-3371 (AP) or dlbaker@memphis.edu (DB)

SUPPLEMENTAL TABLES

Table S6. Known inactive ligands for 5 DUD-E GPCR targets (AA2AR, ADRB1, ADRB2, CXCR4, DRD3).

Ligand Number	Structure[a]	Target	Docking Structure PDBID
1*	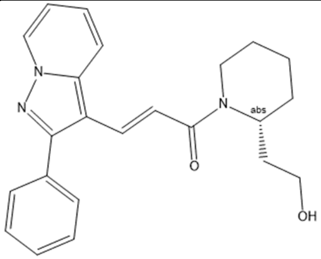	AA2AR	3EML
2*	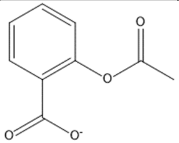	AA2AR	3EML
3*	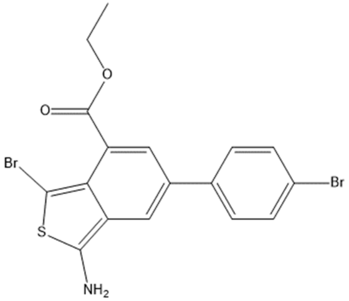	AA2AR	3EML
4*	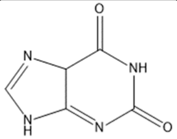	AA2AR	3EML
5*	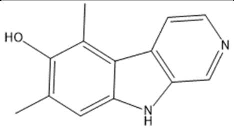	AA2AR	3EML
6*	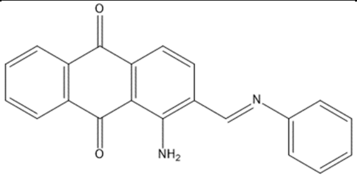	AA2AR	3EML

7*	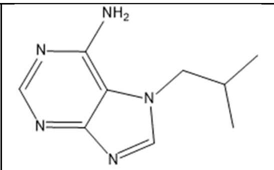	AA2AR	3EML
8*	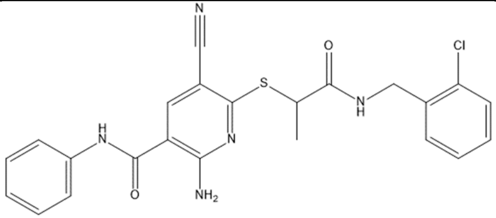	AA2AR	3EML
9*	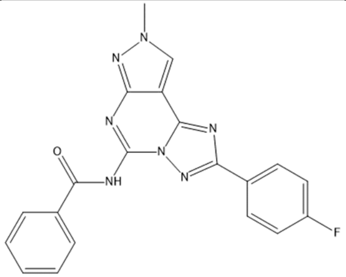	AA2AR	3EML
10*	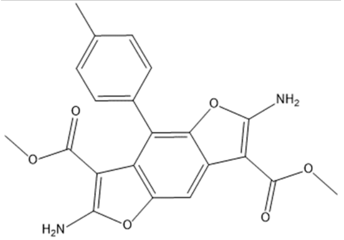	AA2AR	3EML
11*	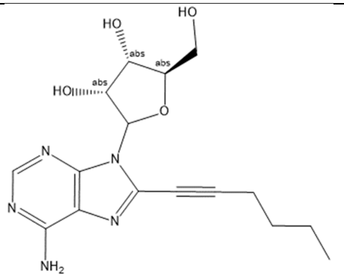	AA2AR	3EML
12*	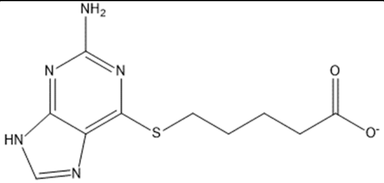	AA2AR	3EML

13*	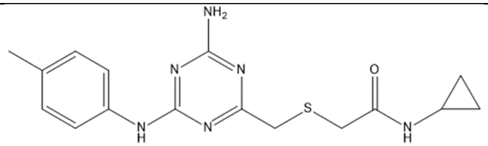	AA2AR	3EML
14*	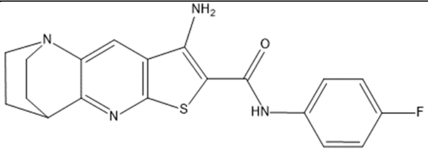	AA2AR	3EML
15*	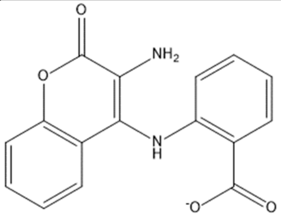	AA2AR	3EML
16*	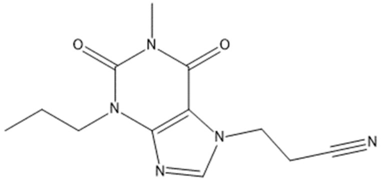	AA2AR	3EML
17*	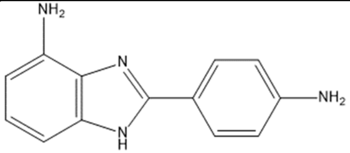	AA2AR	3EML
18*	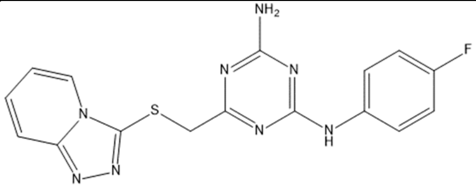	AA2AR	3EML
19*	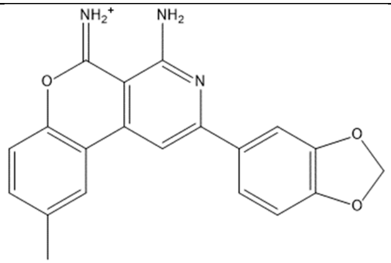	AA2AR	3EML

20*	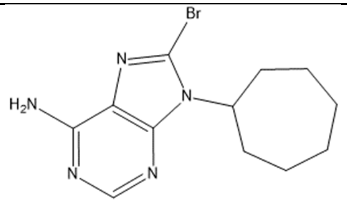	AA2AR	3EML
21*	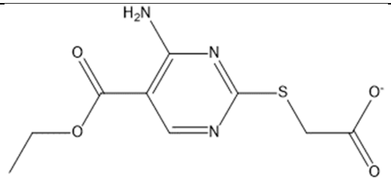	AA2AR	3EML
22*	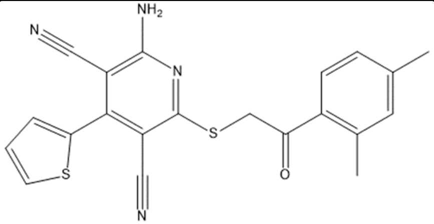	AA2AR	3EML
23*	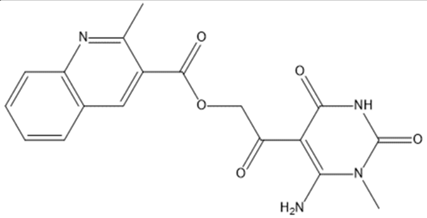	AA2AR	3EML
24*	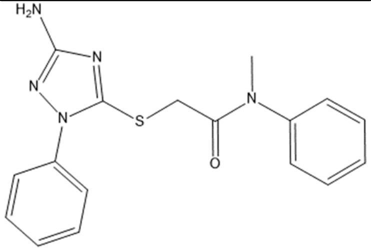	AA2AR	3EML
25*	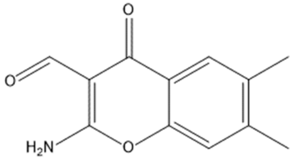	AA2AR	3EML

26	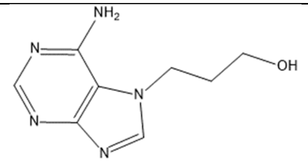	AA2AR	3EML
27	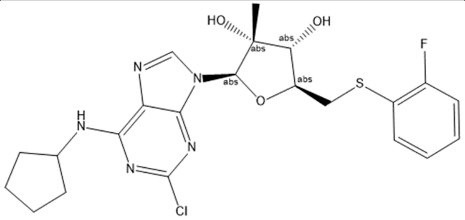	AA2AR	3EML
28	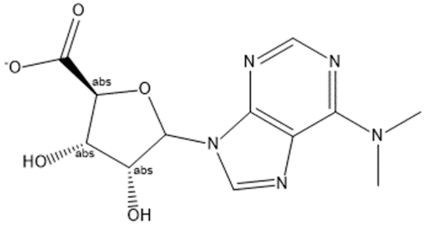	AA2AR	3EML
29	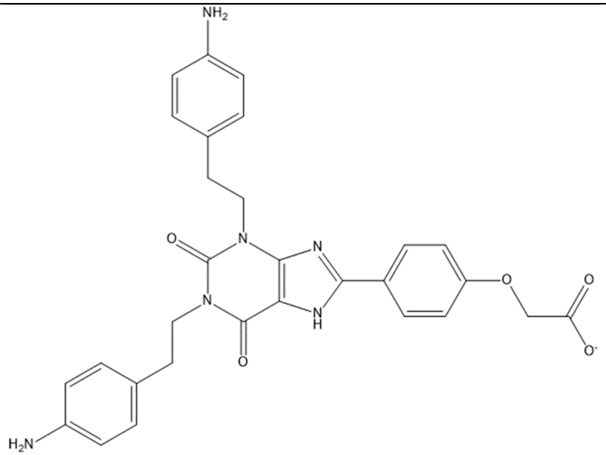	AA2AR	3EML
30	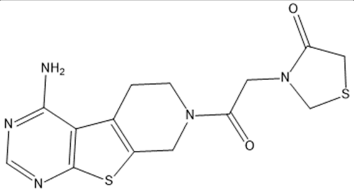	AA2AR	3EML

31	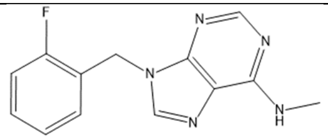	AA2AR	3EML
32	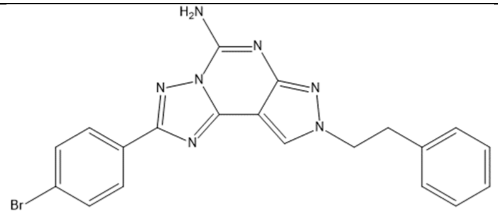	AA2AR	3EML
33	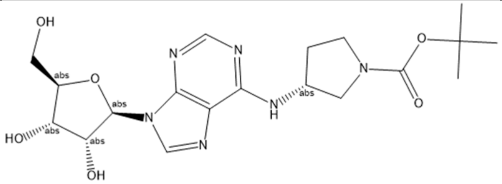	AA2AR	3EML
34	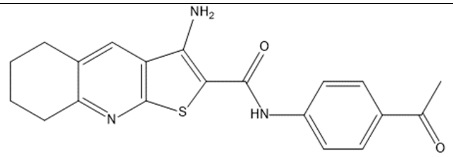	AA2AR	3EML
35	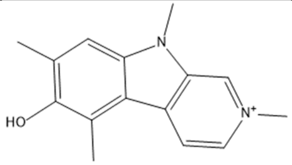	AA2AR	3EML
36	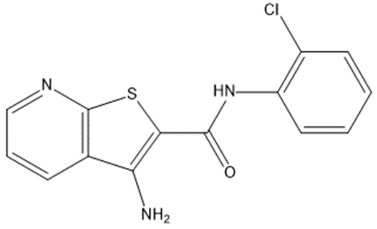	AA2AR	3EML
37	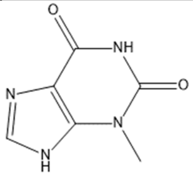	AA2AR	3EML

38	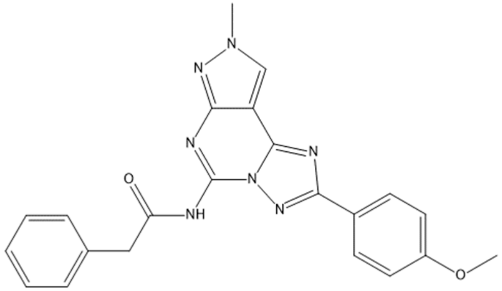	AA2AR	3EML
39	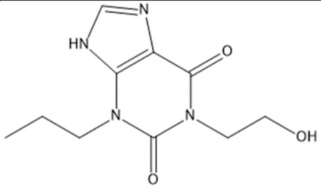	AA2AR	3EML
40	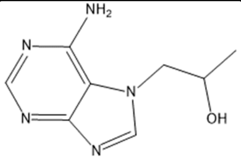	AA2AR	3EML
41	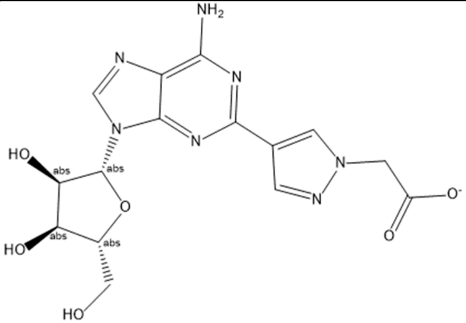	AA2AR	3EML
42	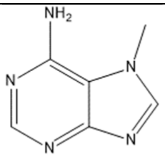	AA2AR	3EML

43	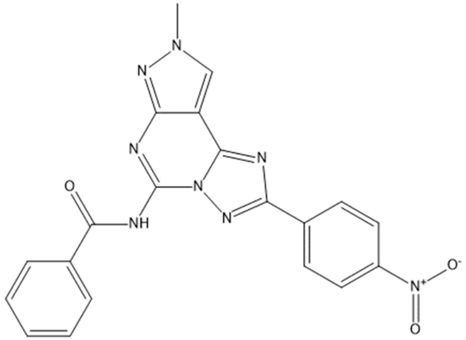	AA2AR	3EML
44	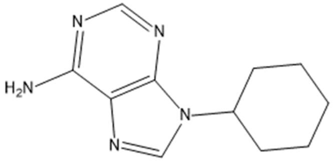	AA2AR	3EML
45	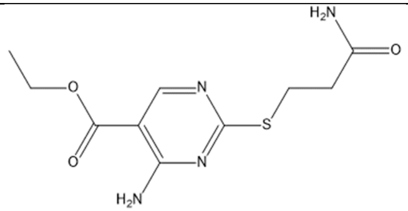	AA2AR	3EML
46	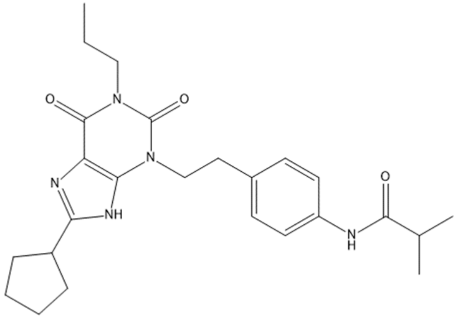	AA2AR	3EML
47	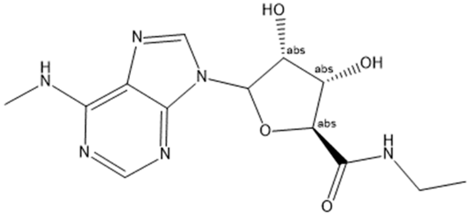	AA2AR	3EML

48	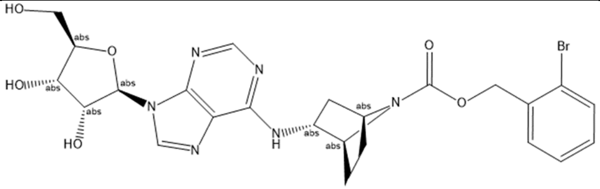	AA2AR	3EML
49	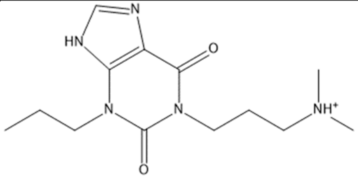	AA2AR	3EML
50	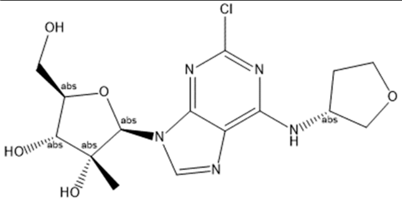	AA2AR	3EML
51	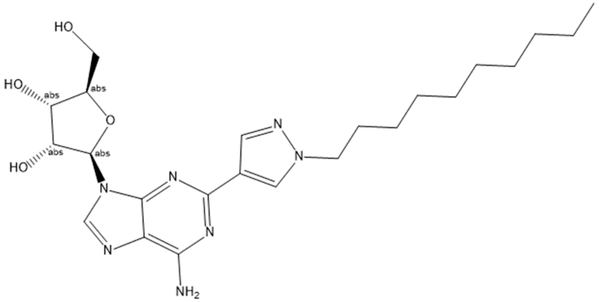	AA2AR	3EML
52	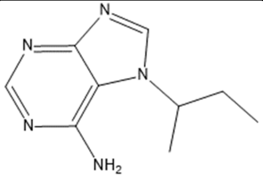	AA2AR	3EML
53	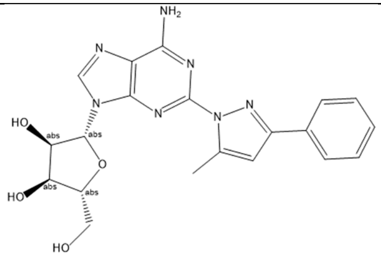	AA2AR	3EML

54	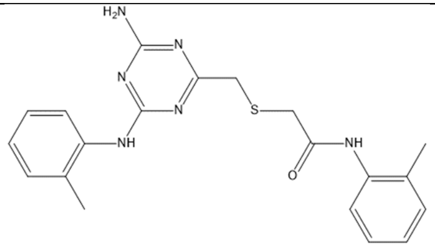	AA2AR	3EML
55	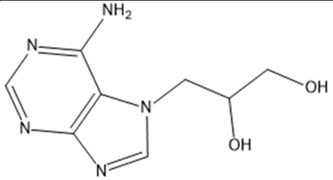	AA2AR	3EML
56	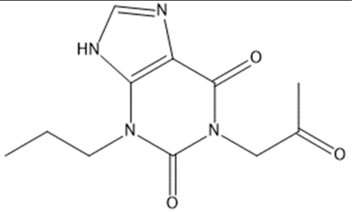	AA2AR	3EML
57	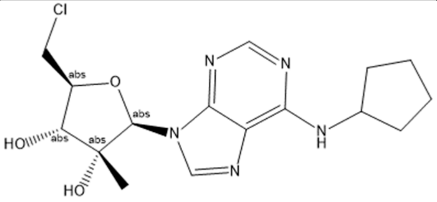	AA2AR	3EML
58	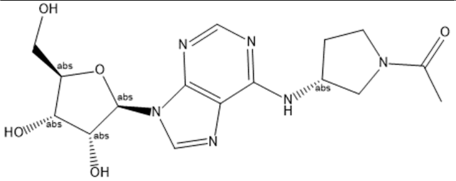	AA2AR	3EML

59	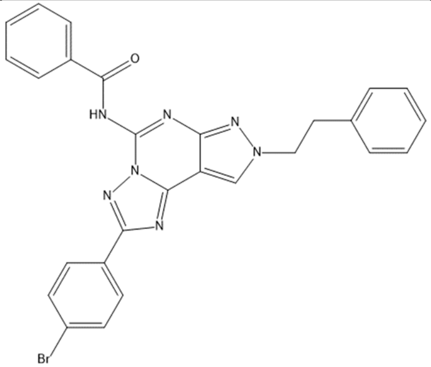	AA2AR	3EML
60	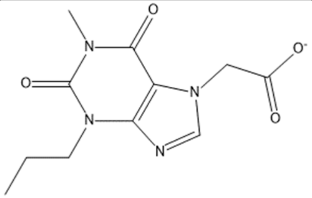	AA2AR	3EML
61	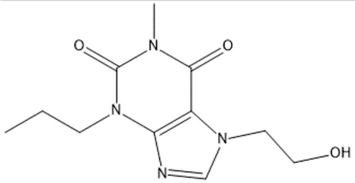	AA2AR	3EML
62	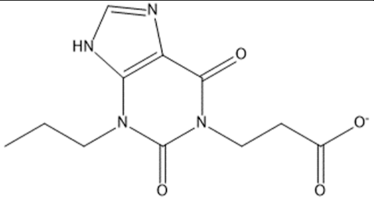	AA2AR	3EML
63	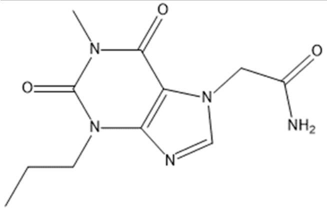	AA2AR	3EML

64	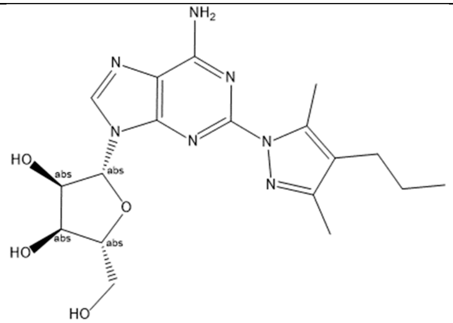	AA2AR	3EML
65	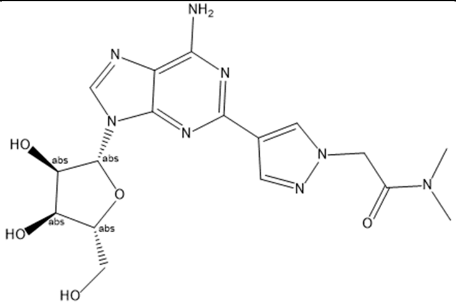	AA2AR	3EML
66	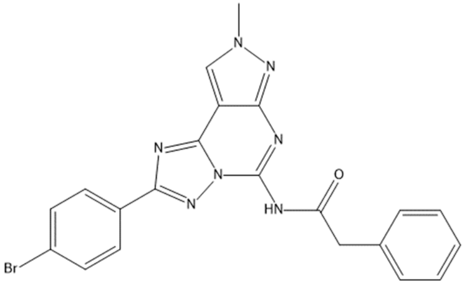	AA2AR	3EML
67	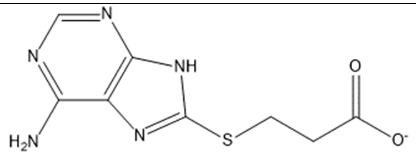	AA2AR	3EML
68	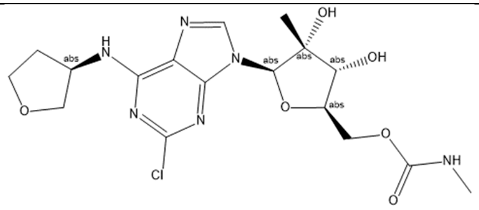	AA2AR	3EML

69	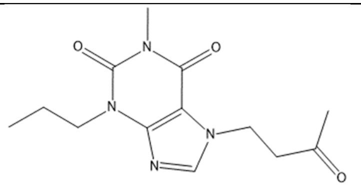	AA2AR	3EML
70	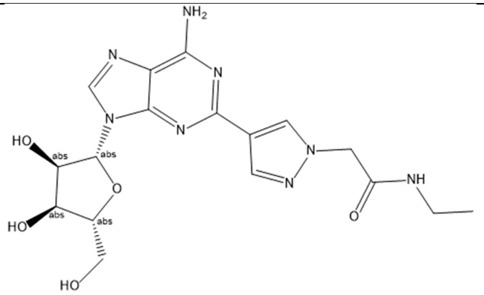	AA2AR	3EML
71	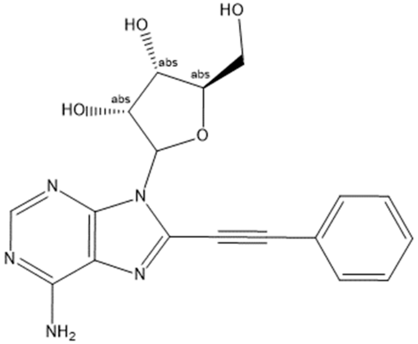	AA2AR	3EML
72	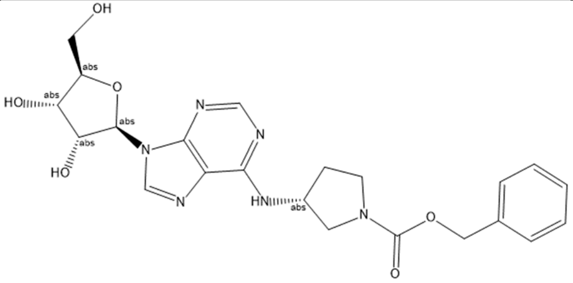	AA2AR	3EML
73	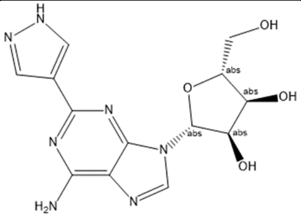	AA2AR	3EML

74	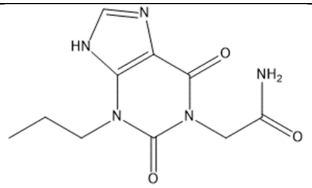	AA2AR	3EML
75	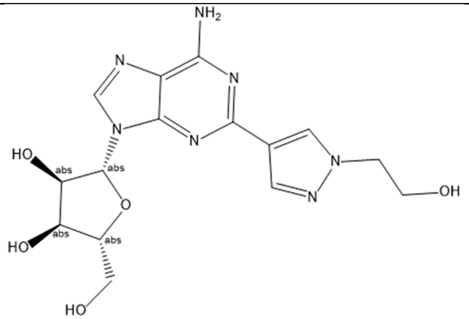	AA2AR	3EML
76	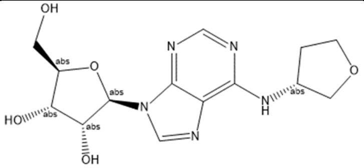	AA2AR	3EML
77	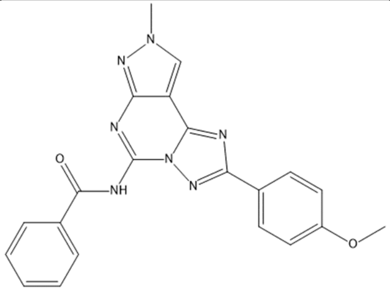	AA2AR	3EML
78	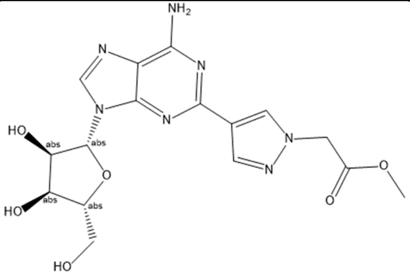	AA2AR	3EML

79	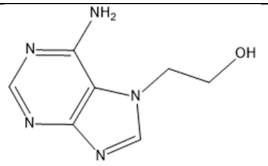	AA2AR	3EML
80	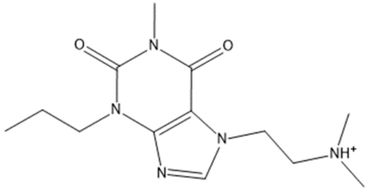	AA2AR	3EML
81	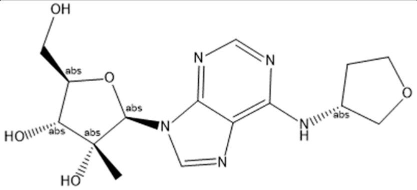	AA2AR	3EML
82	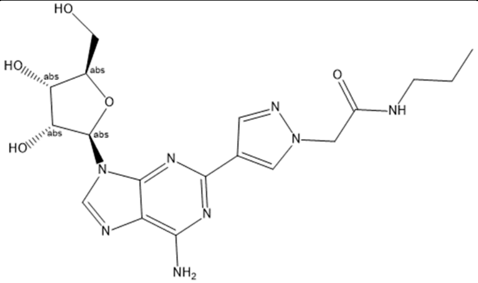	AA2AR	3EML
83	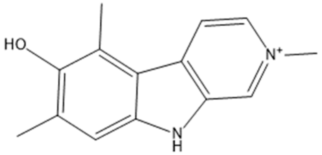	AA2AR	3EML
84	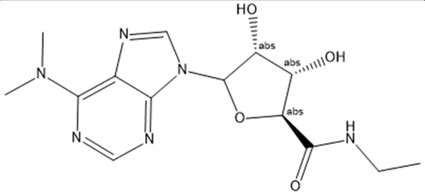	AA2AR	3EML

85	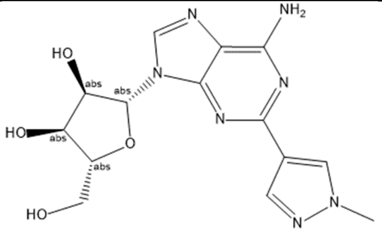	AA2AR	3EML
86	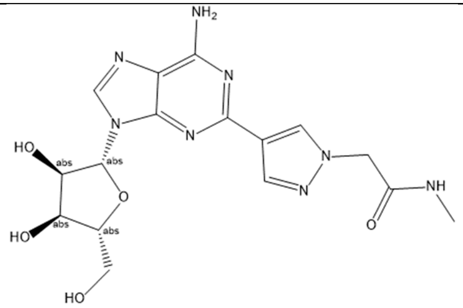	AA2AR	3EML
87	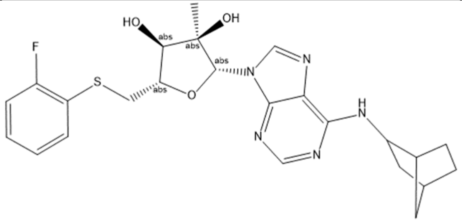	AA2AR	3EML
88	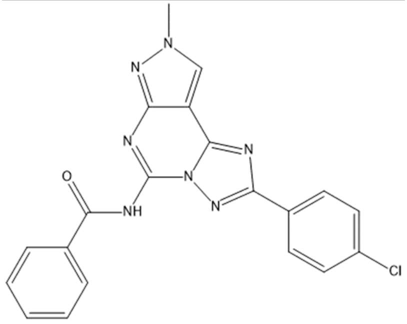	AA2AR	3EML

89	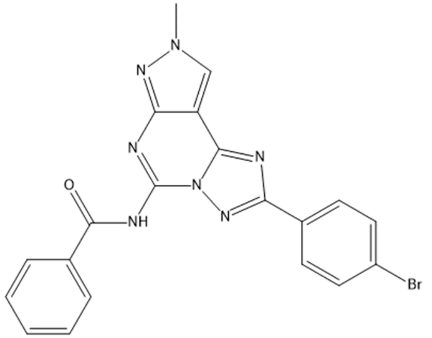	AA2AR	3EML
90	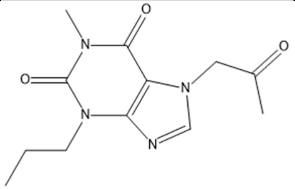	AA2AR	3EML
91	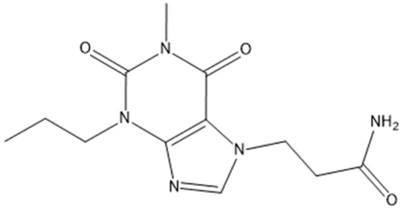	AA2AR	3EML
92	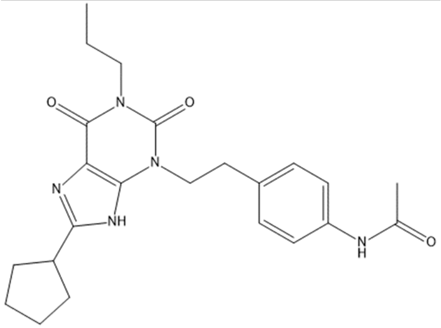	AA2AR	3EML
93	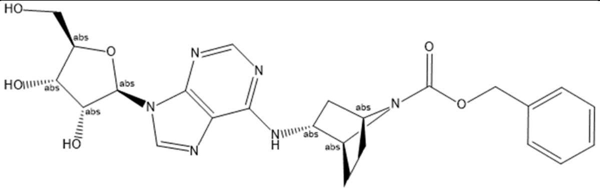	AA2AR	3EML

94	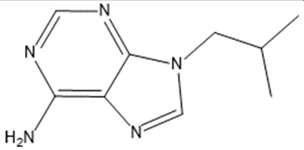	AA2AR	3EML
95	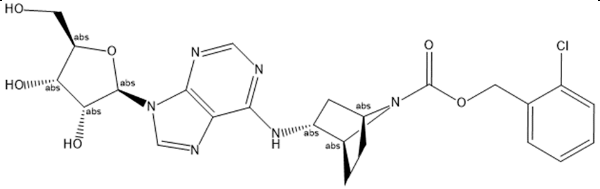	AA2AR	3EML
96	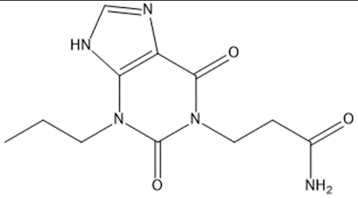	AA2AR	3EML
97	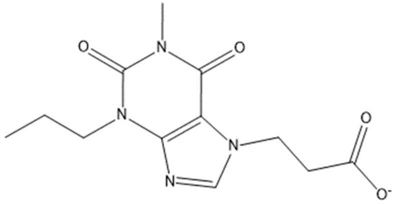	AA2AR	3EML
98	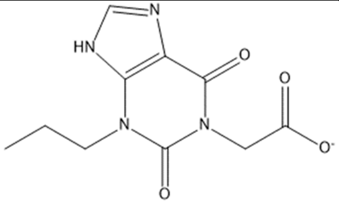	AA2AR	3EML
99	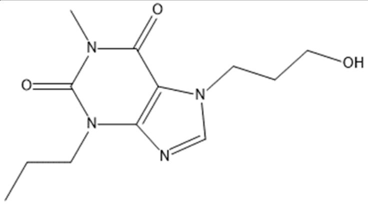	AA2AR	3EML

100	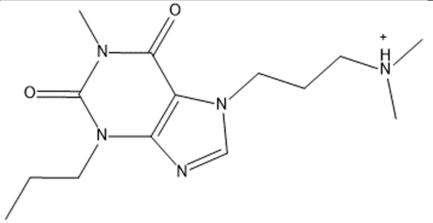	AA2AR	3EML
101	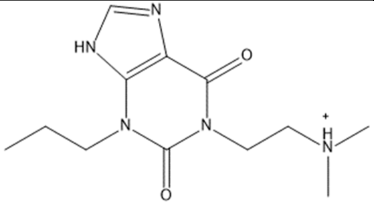	AA2AR	3EML
102	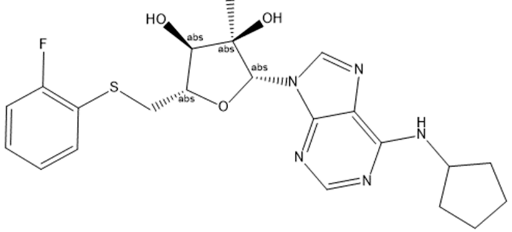	AA2AR	3EML
103	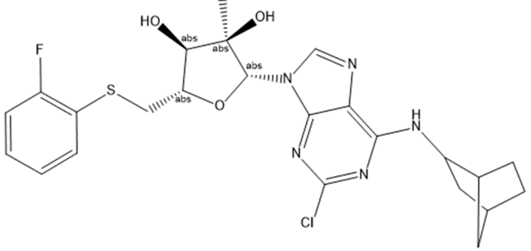	AA2AR	3EML
104	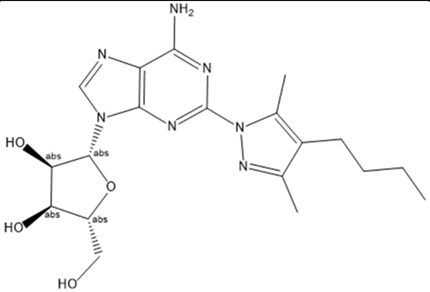	AA2AR	3EML
105*	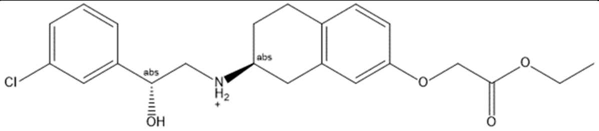	ADRB1	2VT4

106*	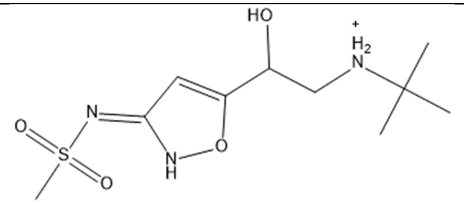	ADRB1	2VT4
107*	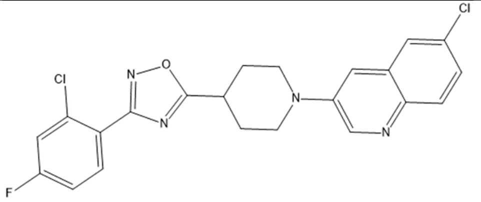	ADRB1	2VT4
108*	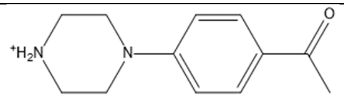	ADRB1	2VT4
109*	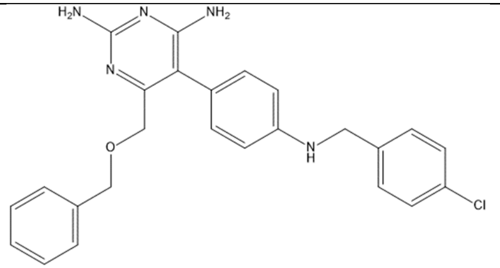	ADRB1	2VT4
110*	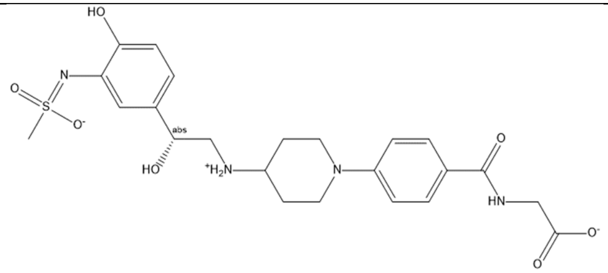	ADRB1	2VT4
111*	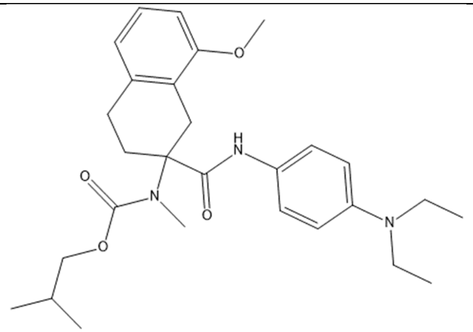	ADRB1	2VT4

112*	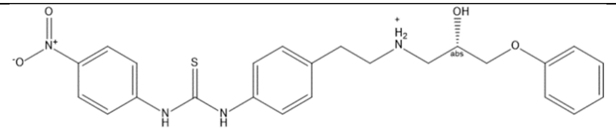	ADRB1	2VT4
113*	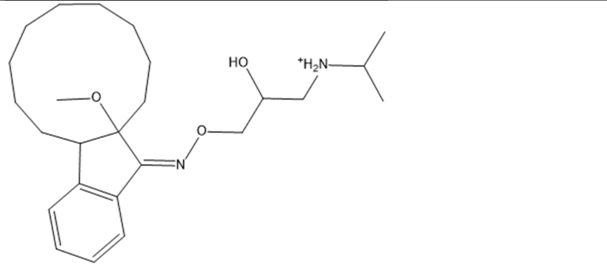	ADRB1	2VT4
114*	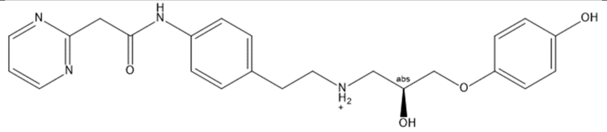	ADRB1	2VT4
115*	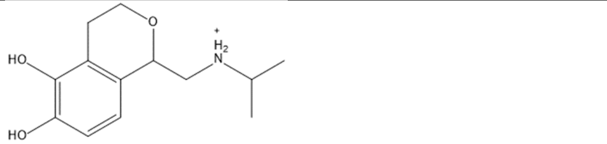	ADRB1	2VT4
116*	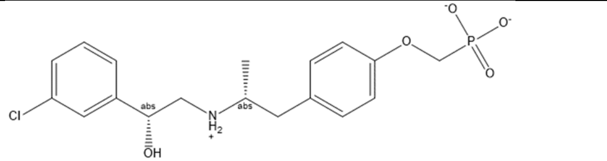	ADRB1	2VT4
117*	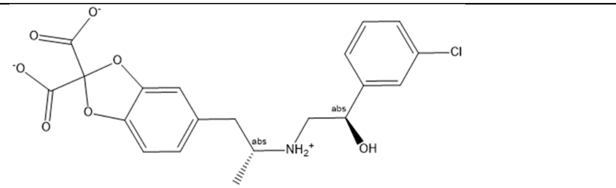	ADRB1	2VT4
118*	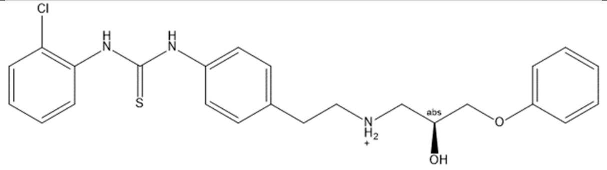	ADRB1	2VT4

119*	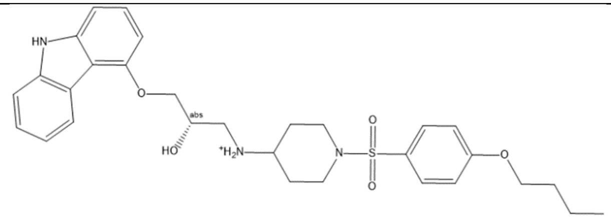	ADRB1	2VT4
120*	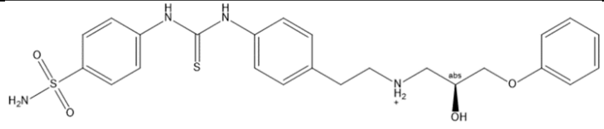	ADRB1	2VT4
121*	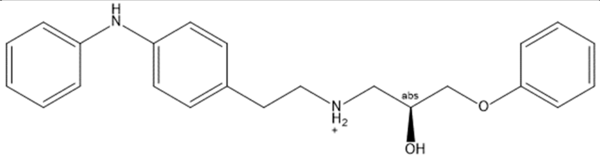	ADRB1	2VT4
122*	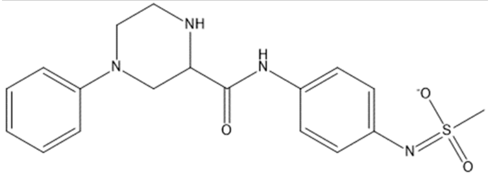	ADRB1	2VT4
123*	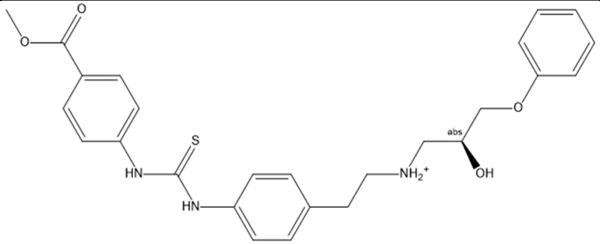	ADRB1	2VT4
124*	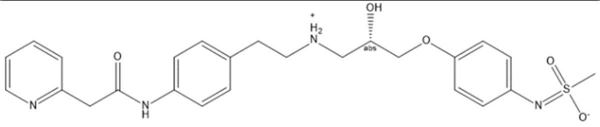	ADRB1	2VT4
125*	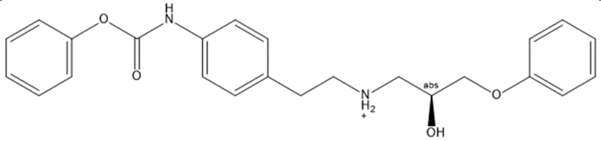	ADRB1	2VT4

126*	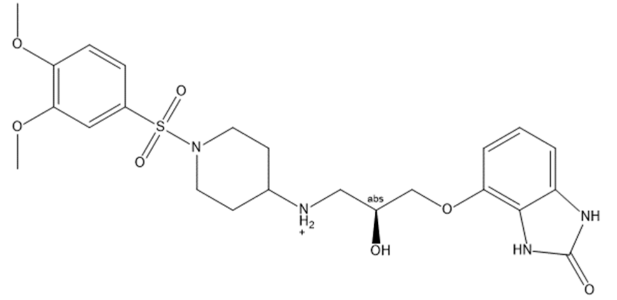	ADRB1	2VT4
127*	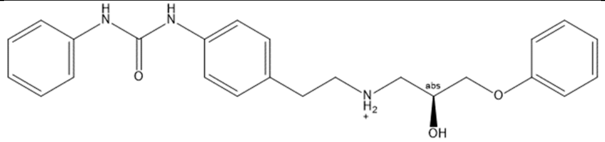	ADRB1	2VT4
128*	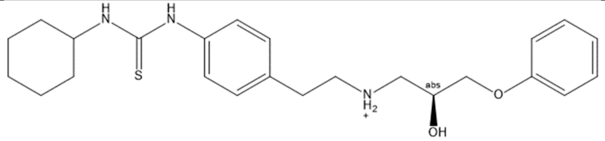	ADRB1	2VT4
129*	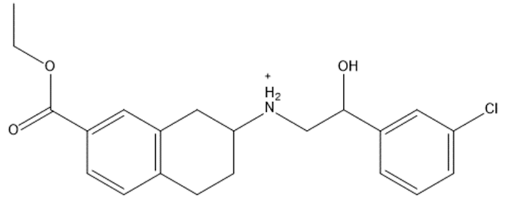	ADRB1	2VT4
130	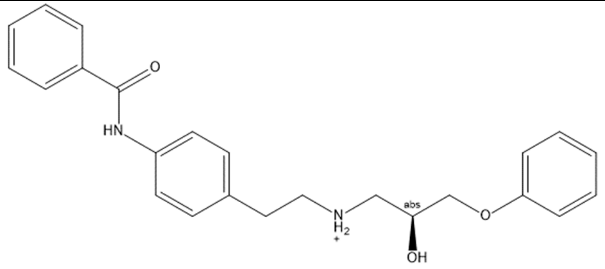	ADRB1	2VT4
131	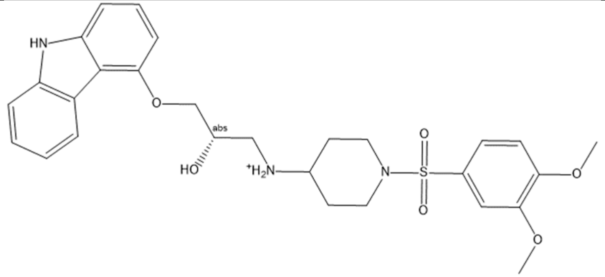	ADRB1	2VT4

132	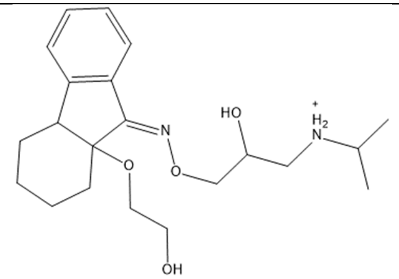	ADRB1	2VT4
133	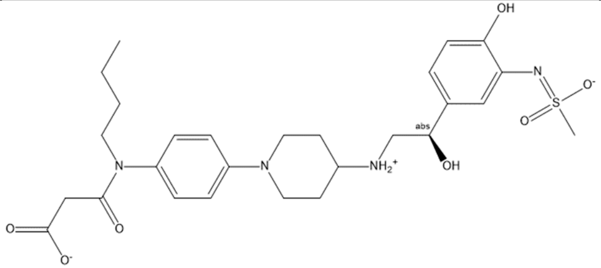	ADRB1	2VT4
134	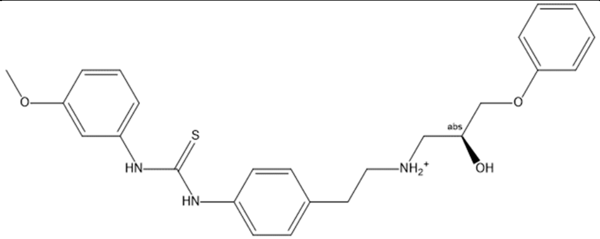	ADRB1	2VT4
135	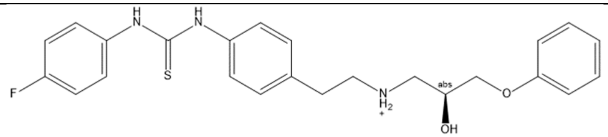	ADRB1	2VT4
136	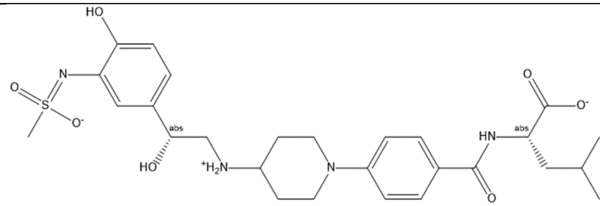	ADRB1	2VT4
137	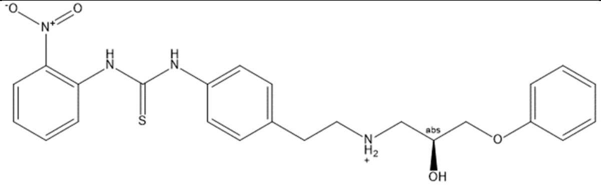	ADRB1	2VT4

138	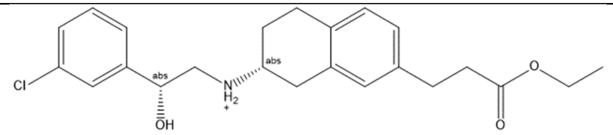	ADRB1	2VT4
139	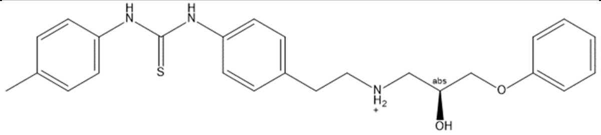	ADRB1	2VT4
140	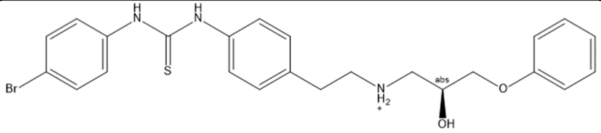	ADRB1	2VT4
141	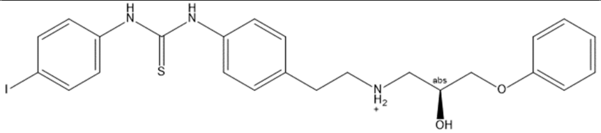	ADRB1	2VT4
142	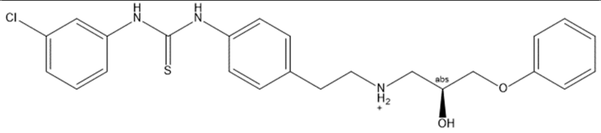	ADRB1	2VT4
143	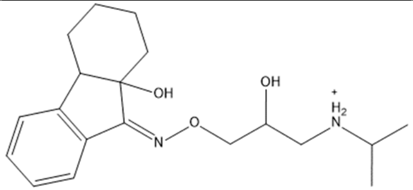	ADRB1	2VT4
144	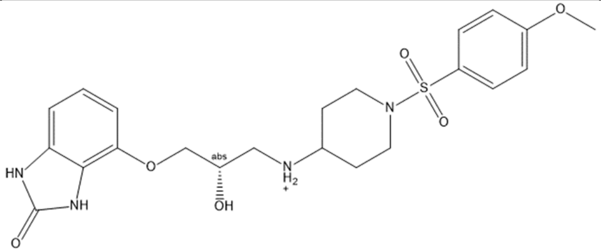	ADRB1	2VT4
145	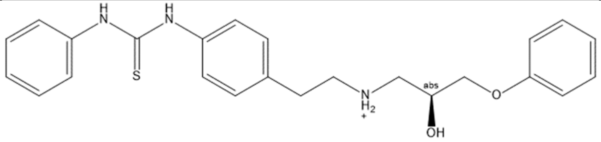	ADRB1	2VT4

146	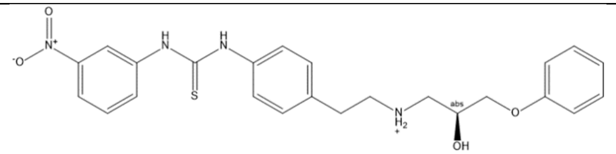	ADRB1	2VT4
147	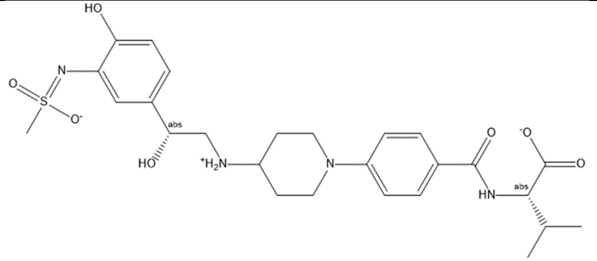	ADRB1	2VT4
148	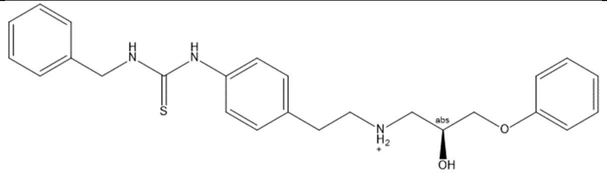	ADRB1	2VT4
149	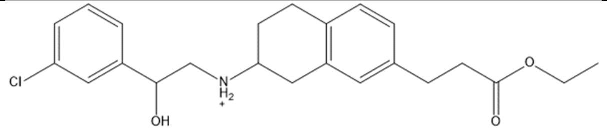	ADRB1	2VT4
150	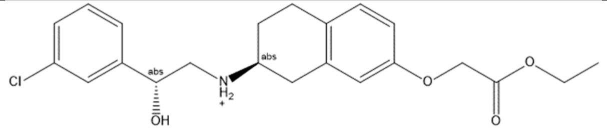	ADRB1	2VT4
151	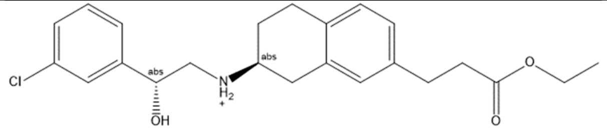	ADRB1	2VT4
152	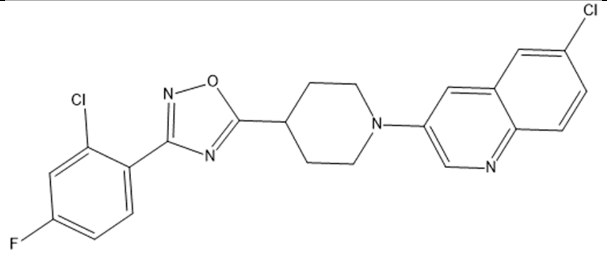	ADRB1	2VT4

153	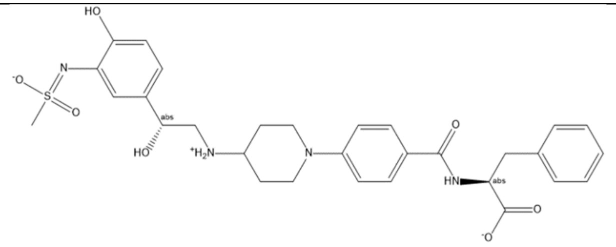	ADRB1	2VT4
154	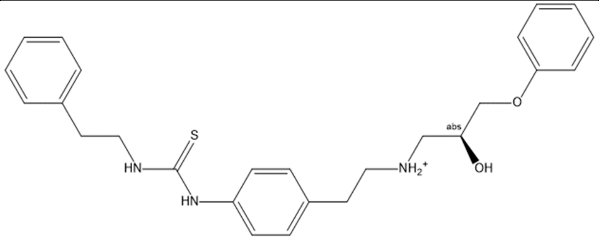	ADRB1	2VT4
155	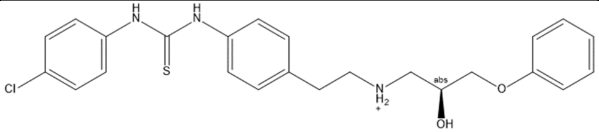	ADRB1	2VT4
156	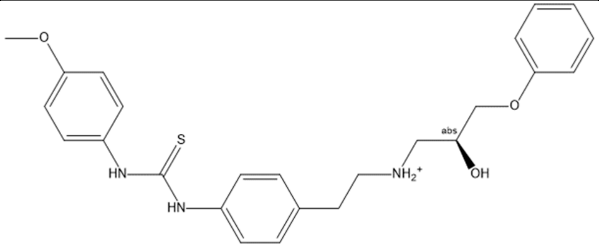	ADRB1	2VT4
157*	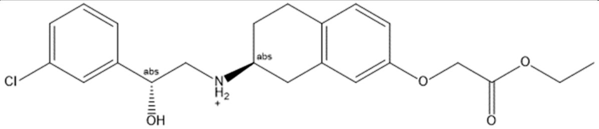	ADRB2	3NY8
158*	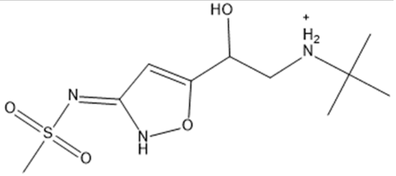	ADRB2	3NY8
159*	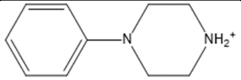	ADRB2	3NY8

160*	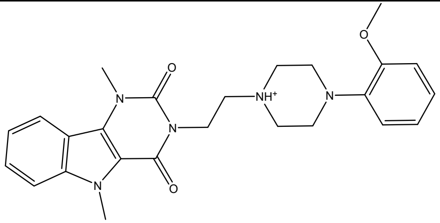	ADRB2	3NY8
161*	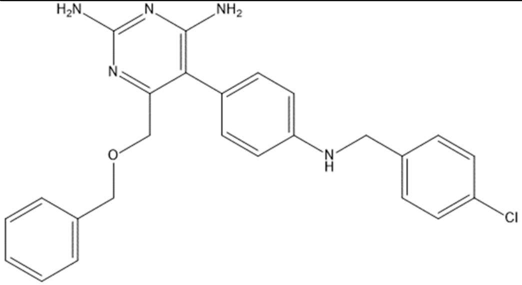	ADRB2	3NY8
162*	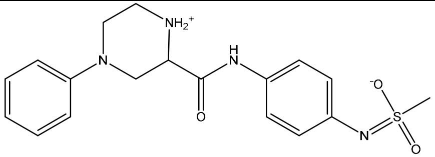	ADRB2	3NY8
163*	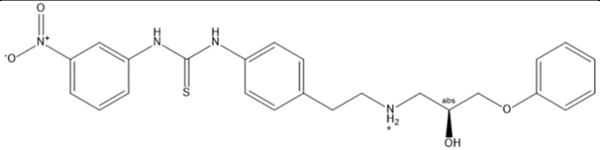	ADRB2	3NY8
164*	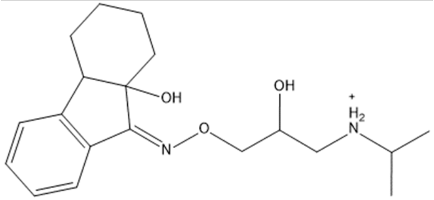	ADRB2	3NY8
165*	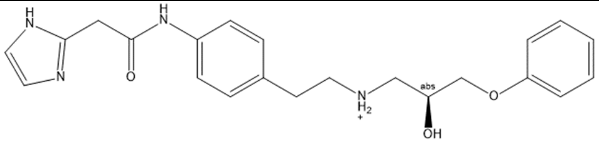	ADRB2	3NY8
166*	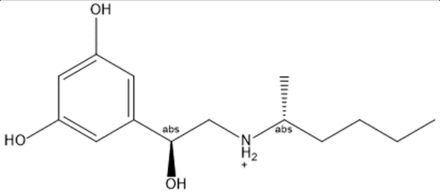	ADRB2	3NY8

167*	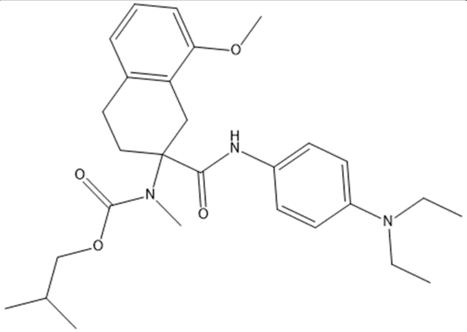	ADRB2	3NY8
168*	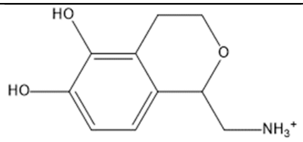	ADRB2	3NY8
169*	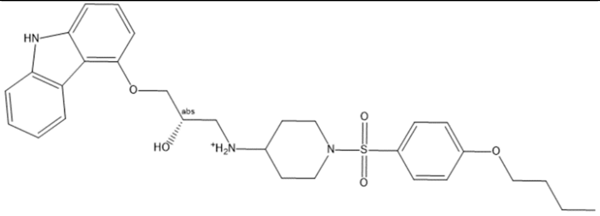	ADRB2	3NY8
170*	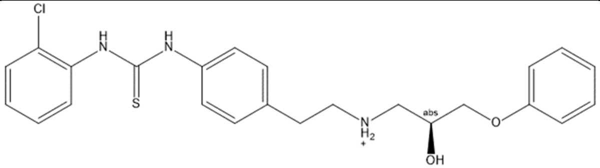	ADRB2	3NY8
171*	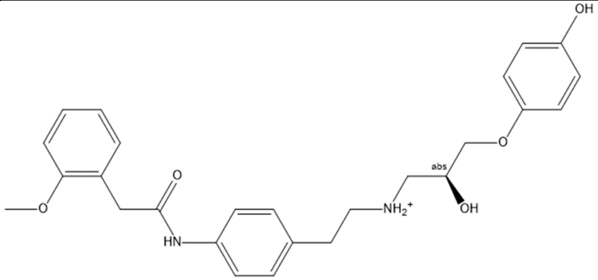	ADRB2	3NY8
172*	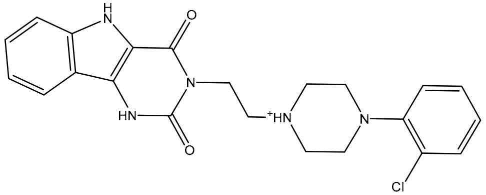	ADRB2	3NY8

173*	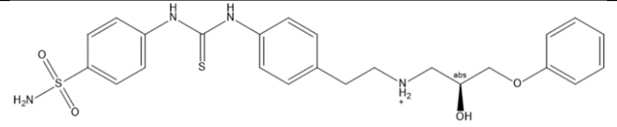	ADRB2	3NY8
174*	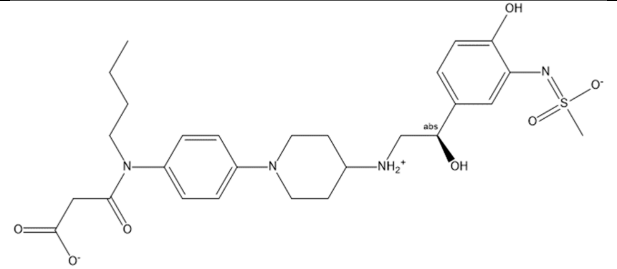	ADRB2	3NY8
175*	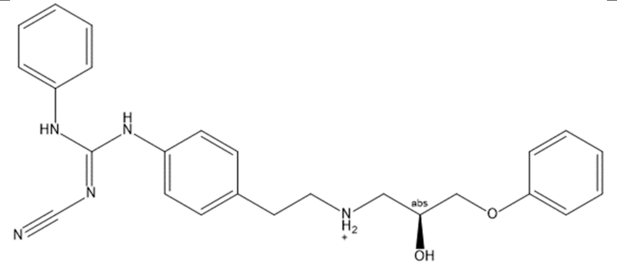	ADRB2	3NY8
176*	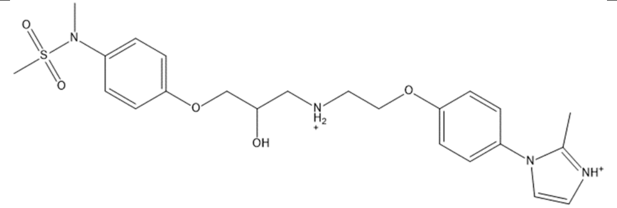	ADRB2	3NY8
177*	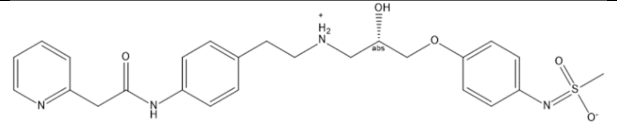	ADRB2	3NY8
178*	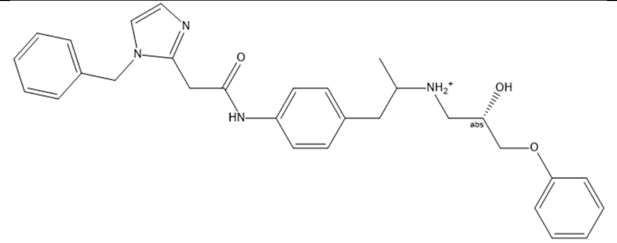	ADRB2	3NY8
179*	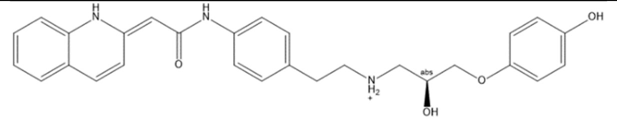	ADRB2	3NY8

180*	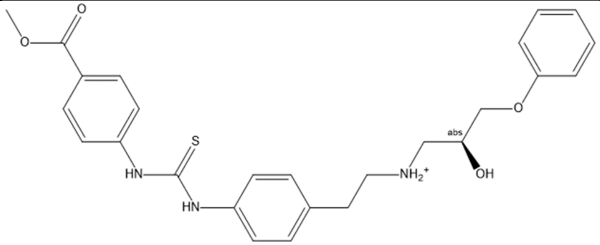	ADRB2	3NY8
181*	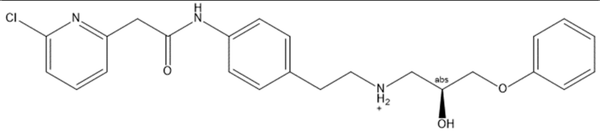	ADRB2	3NY8
182	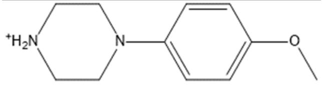	ADRB2	3NY8
183	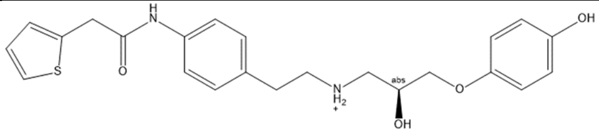	ADRB2	3NY8
184	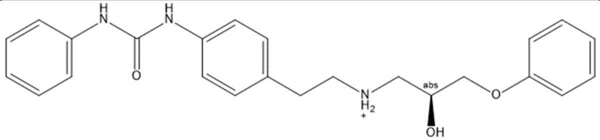	ADRB2	3NY8
185	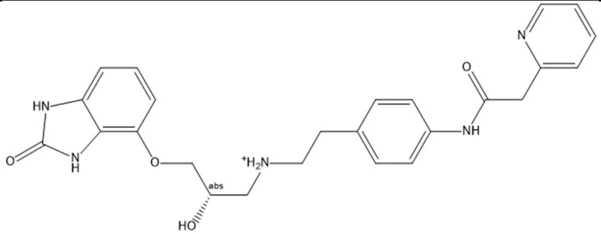	ADRB2	3NY8
186	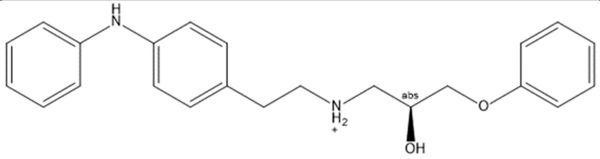	ADRB2	3NY8

187	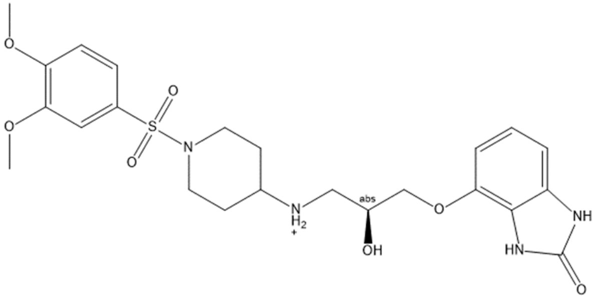	ADRB2	3NY8
188	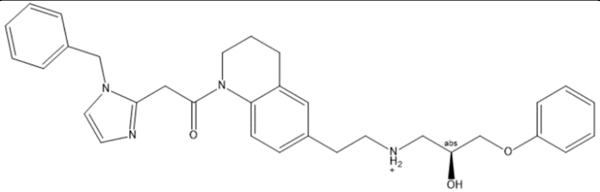	ADRB2	3NY8
189	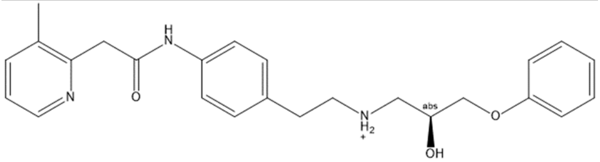	ADRB2	3NY8
190	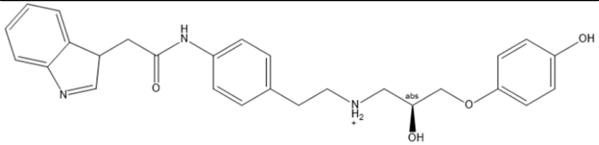	ADRB2	3NY8
191	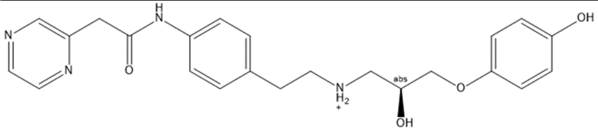	ADRB2	3NY8
192	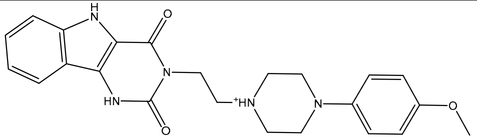	ADRB2	3NY8
193	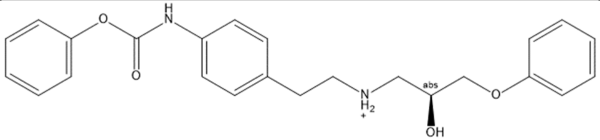	ADRB2	3NY8
194	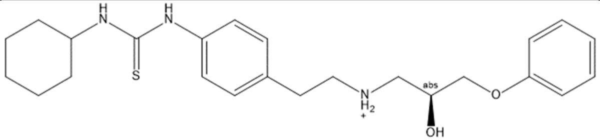	ADRB2	3NY8

195	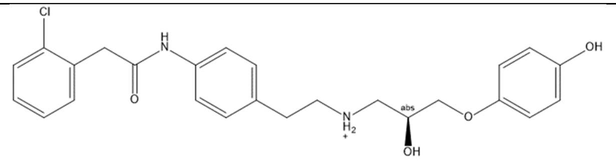	ADRB2	3NY8
196	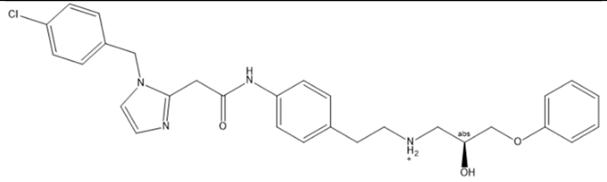	ADRB2	3NY8
197	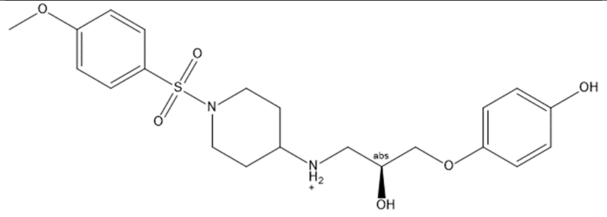	ADRB2	3NY8
198	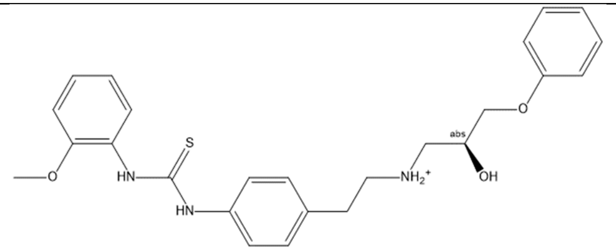	ADRB2	3NY8
199	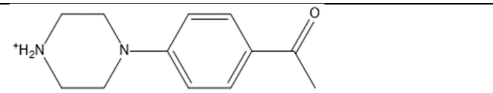	ADRB2	3NY8
200	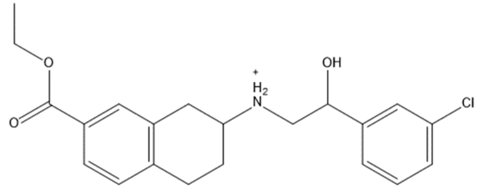	ADRB2	3NY8
201	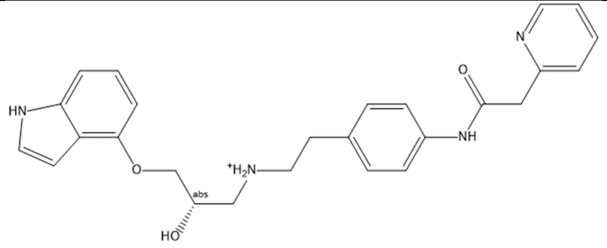	ADRB2	3NY8

202	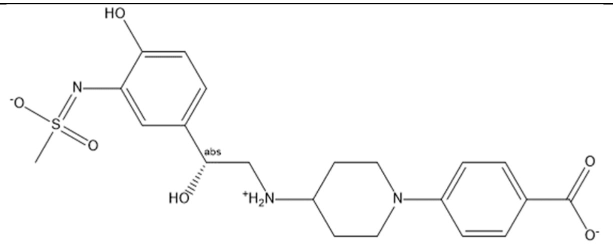	ADRB2	3NY8
203	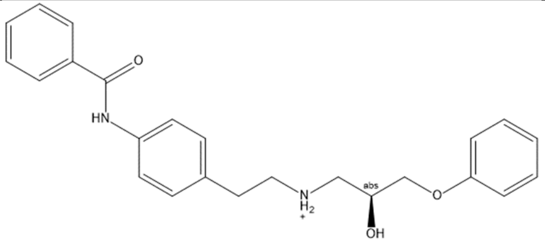	ADRB2	3NY8
204	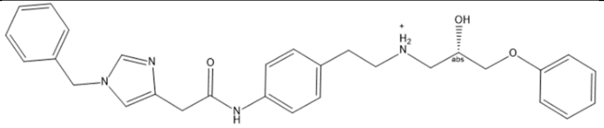	ADRB2	3NY8
205	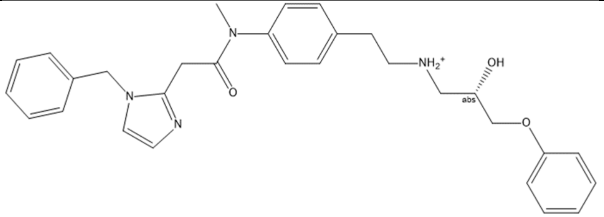	ADRB2	3NY8
206	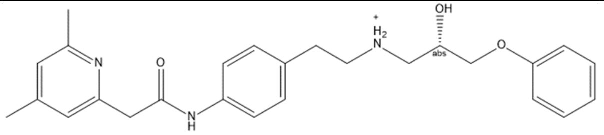	ADRB2	3NY8
207	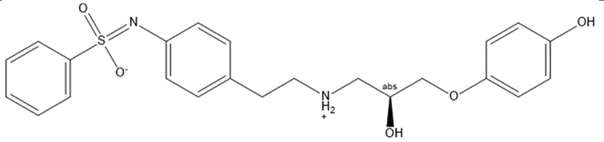	ADRB2	3NY8
208	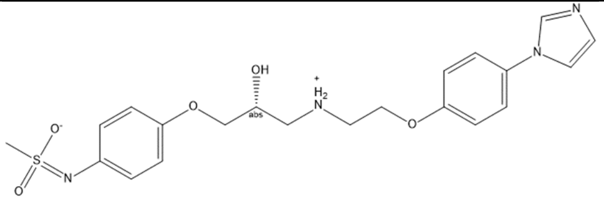	ADRB2	3NY8

209	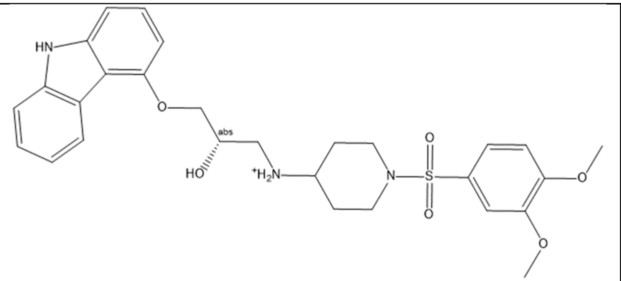	ADRB2	3NY8
210	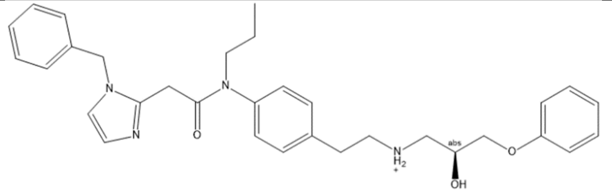	ADRB2	3NY8
211	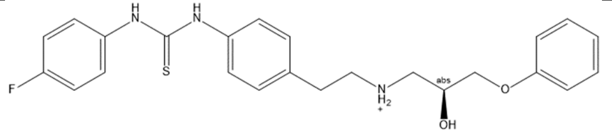	ADRB2	3NY8
212	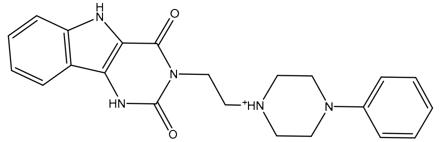	ADRB2	3NY8
213	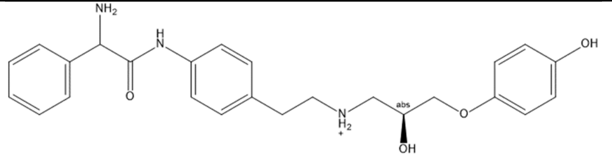	ADRB2	3NY8
214	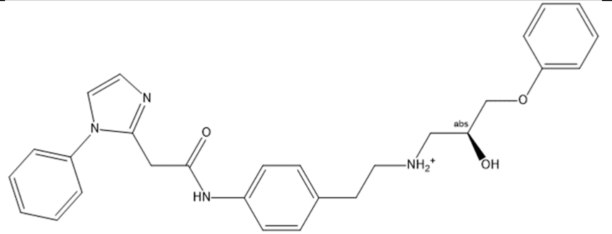	ADRB2	3NY8
215	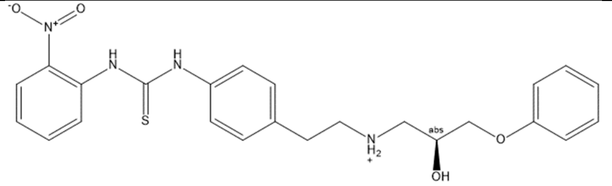	ADRB2	3NY8

216	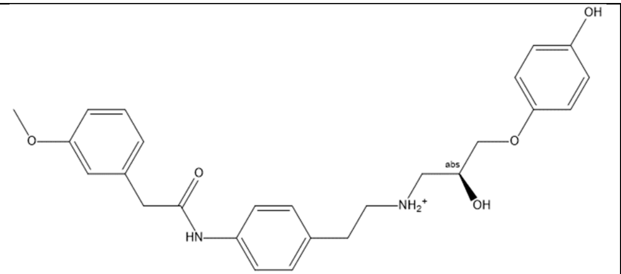	ADRB2	3NY8
217	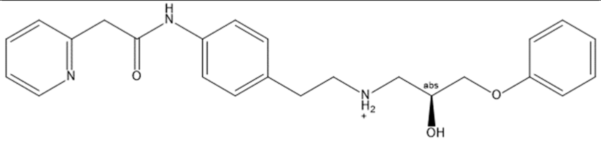	ADRB2	3NY8
218	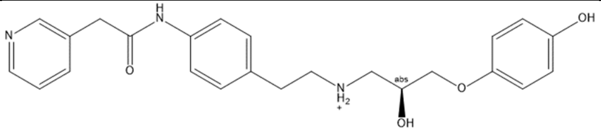	ADRB2	3NY8
219	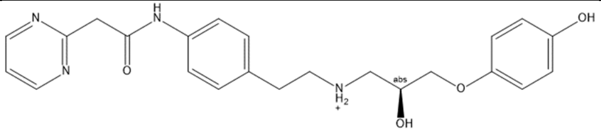	ADRB2	3NY8
220	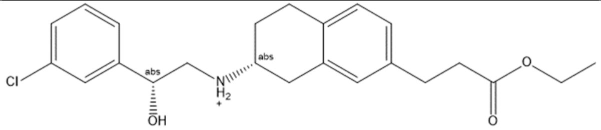	ADRB2	3NY8
221	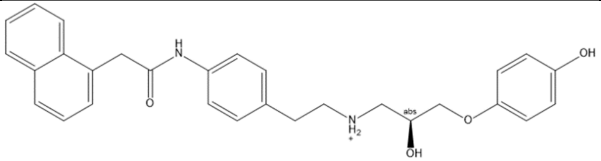	ADRB2	3NY8
222	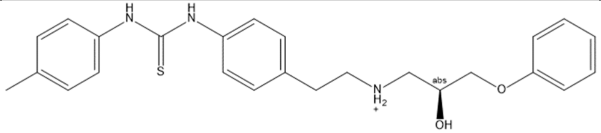	ADRB2	3NY8
223	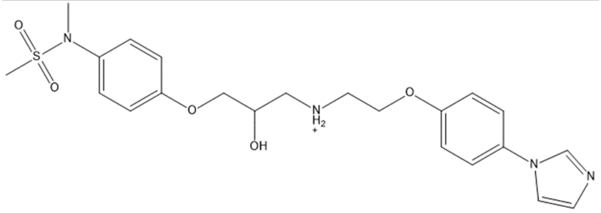	ADRB2	3NY8

224	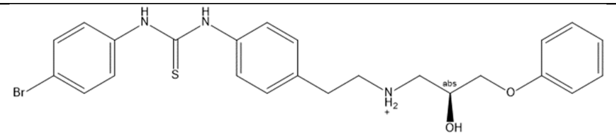	ADRB2	3NY8
225	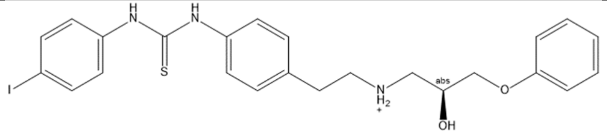	ADRB2	3NY8
226	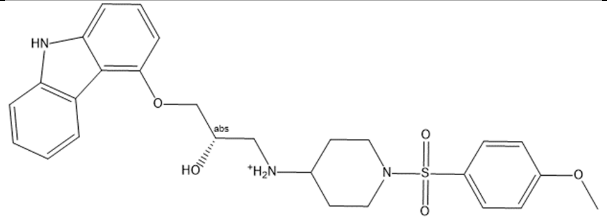	ADRB2	3NY8
227	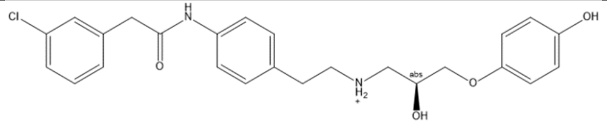	ADRB2	3NY8
228	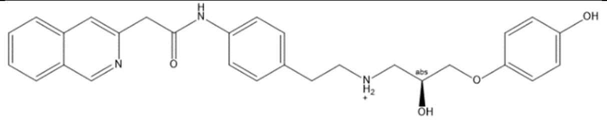	ADRB2	3NY8
229	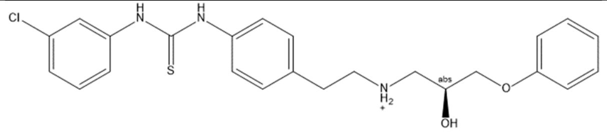	ADRB2	3NY8
230	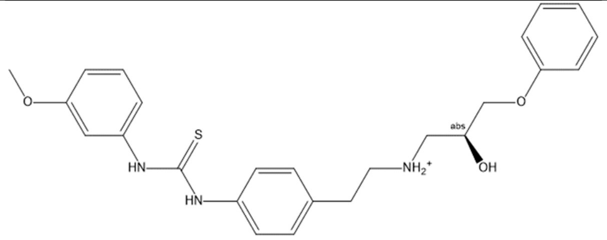	ADRB2	3NY8
231	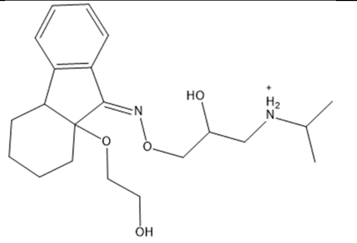	ADRB2	3NY8

232	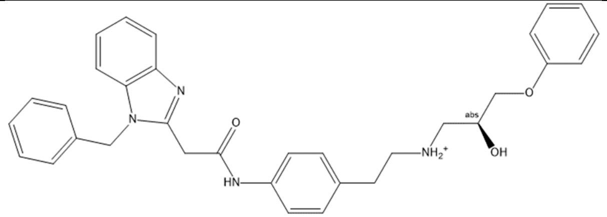	ADRB2	3NY8
233	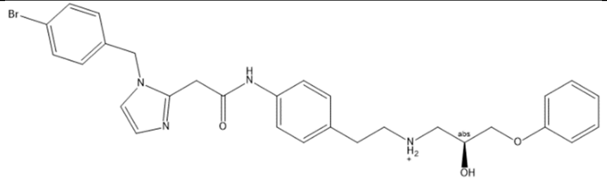	ADRB2	3NY8
234	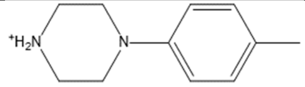	ADRB2	3NY8
235	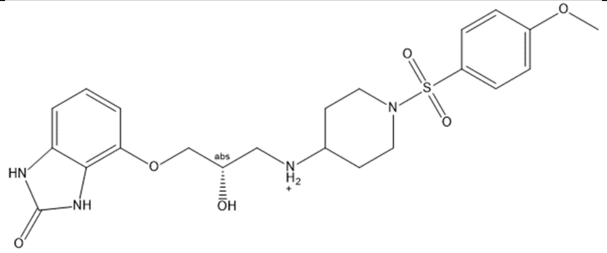	ADRB2	3NY8
236	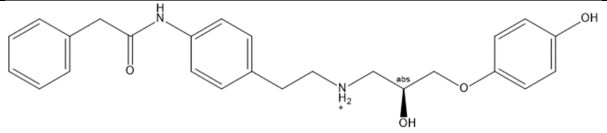	ADRB2	3NY8
237	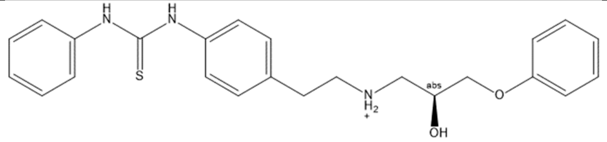	ADRB2	3NY8
238	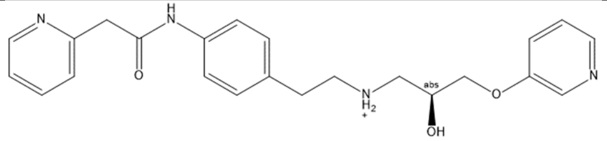	ADRB2	3NY8
239	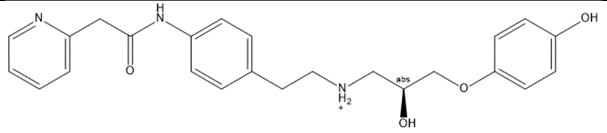	ADRB2	3NY8

240	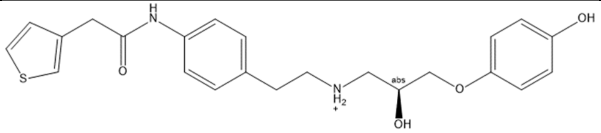	ADRB2	3NY8
241	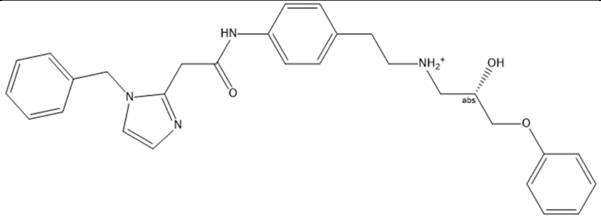	ADRB2	3NY8
242	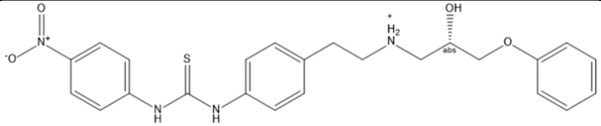	ADRB2	3NY8
243	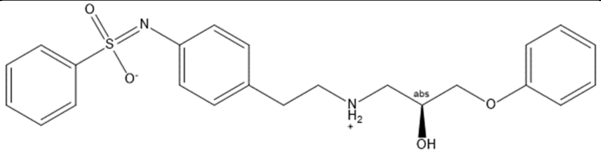	ADRB2	3NY8
244	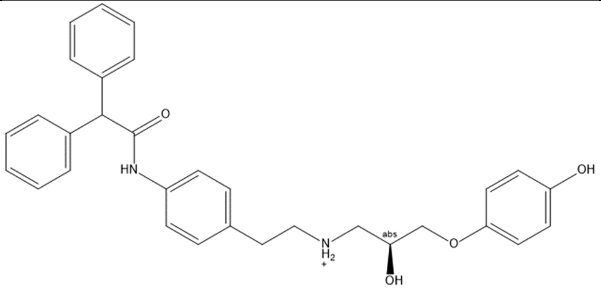	ADRB2	3NY8
245	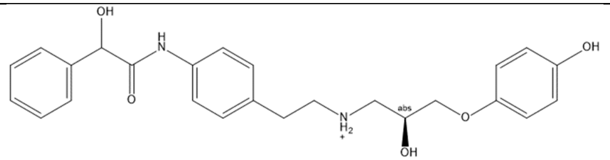	ADRB2	3NY8
246	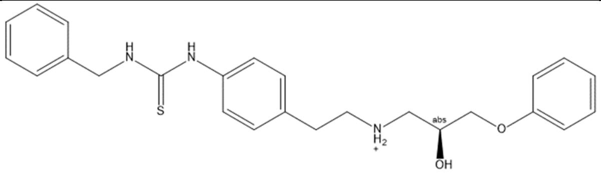	ADRB2	3NY8

247	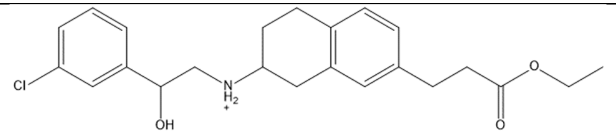	ADRB2	3NY8
248	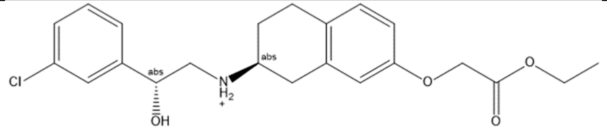	ADRB2	3NY8
249	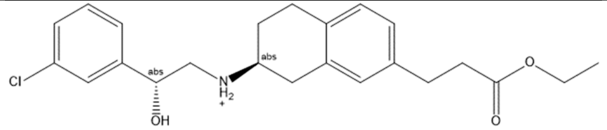	ADRB2	3NY8
250	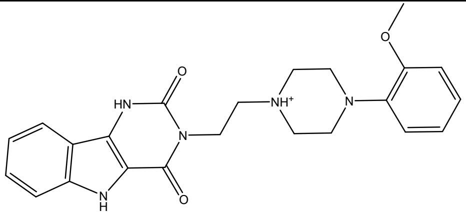	ADRB2	3NY8
251	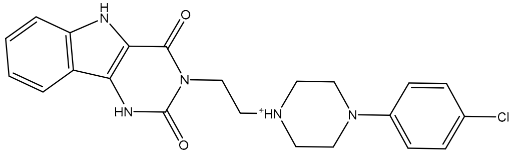	ADRB2	3NY8
252	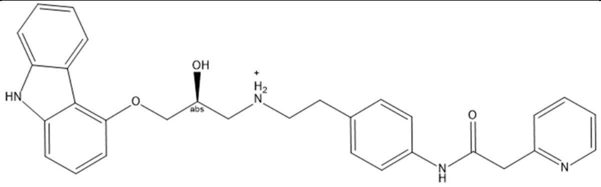	ADRB2	3NY8
253	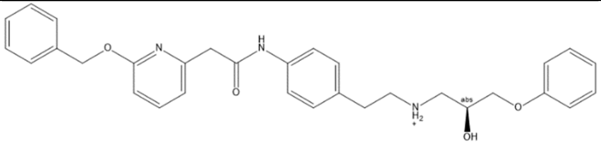	ADRB2	3NY8
254	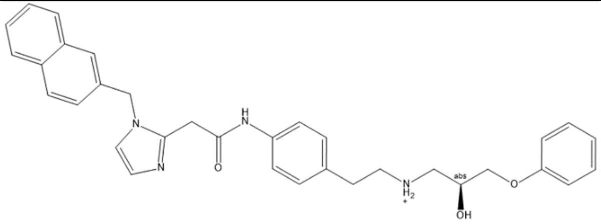	ADRB2	3NY8

255	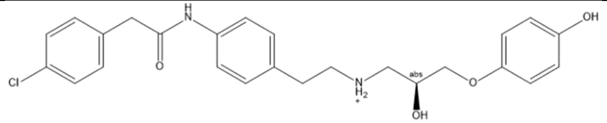	ADRB2	3NY8
256	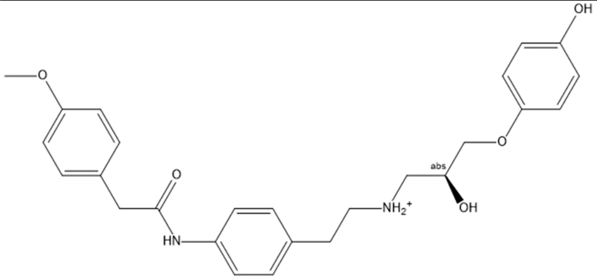	ADRB2	3NY8
257	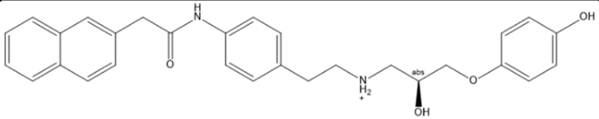	ADRB2	3NY8
258	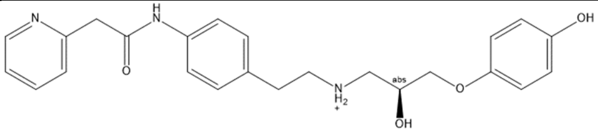	ADRB2	3NY8
259	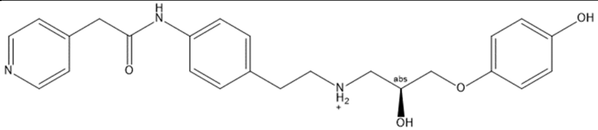	ADRB2	3NY8
260	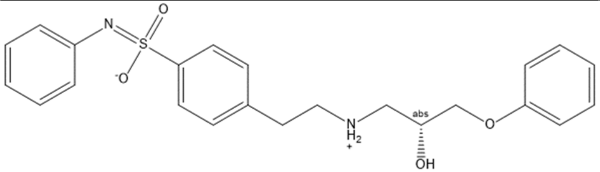	ADRB2	3NY8
261	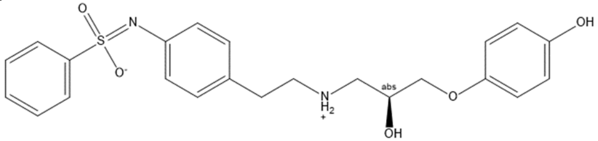	ADRB2	3NY8
262	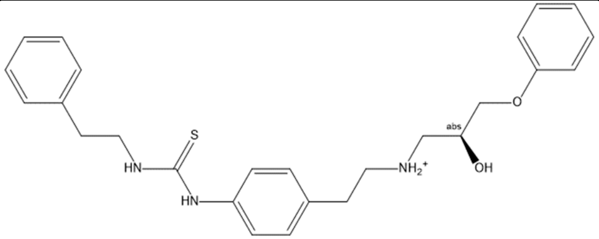	ADRB2	3NY8

263	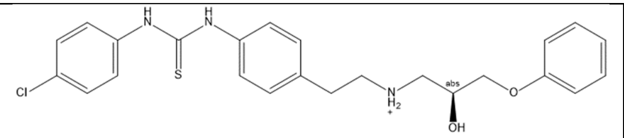	ADRB2	3NY8
264	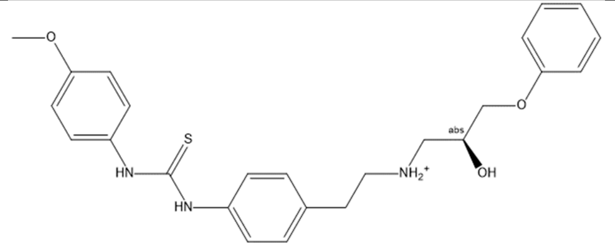	ADRB2	3NY8
265*	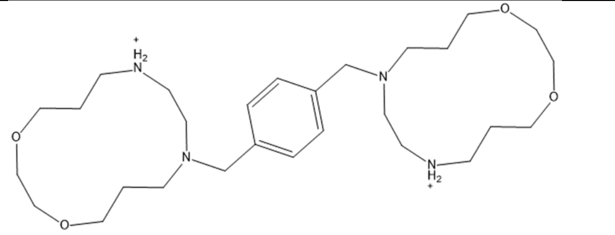	CXCR4	3ODU
266*	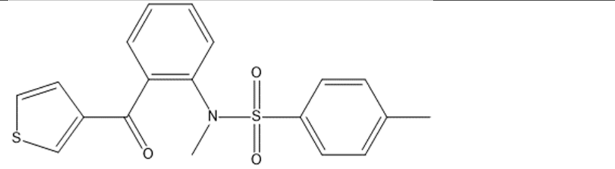	CXCR4	3ODU
267*	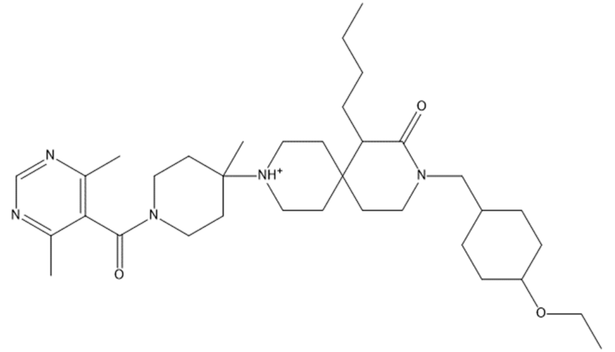	CXCR4	3ODU
268*	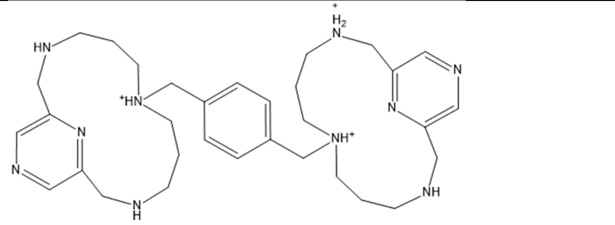	CXCR4	3ODU

269*	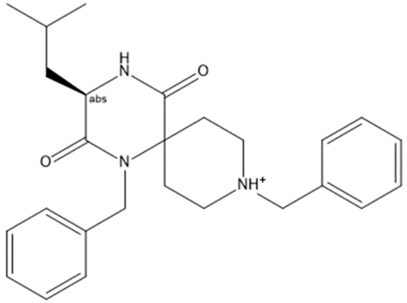	CXCR4	3ODU
270*	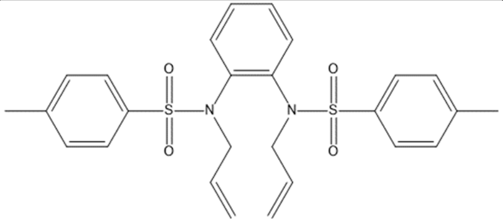	CXCR4	3ODU
271*	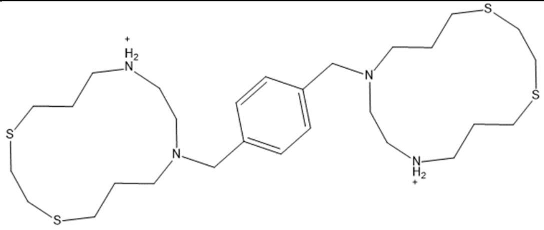	CXCR4	3ODU
272*	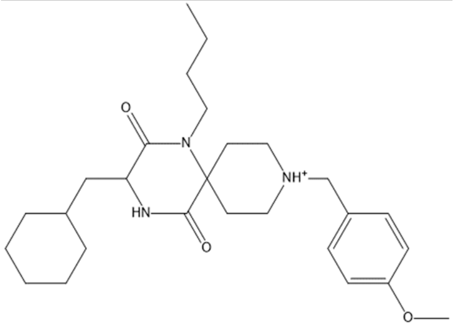	CXCR4	3ODU
273*	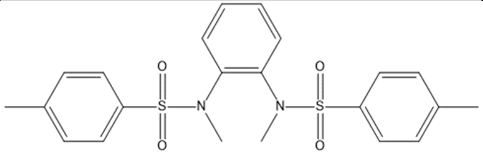	CXCR4	3ODU

274*	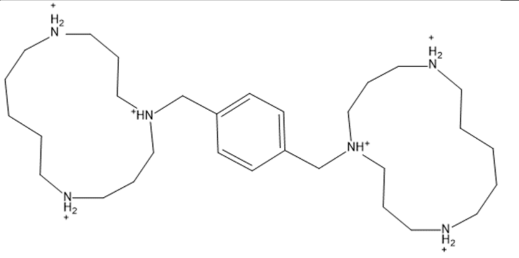	CXCR4	3ODU
275*	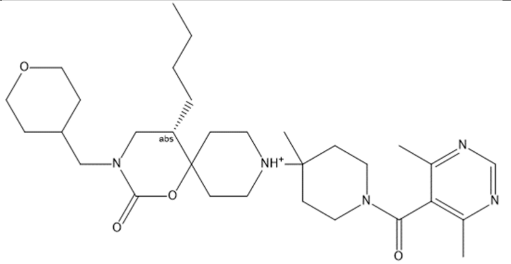	CXCR4	3ODU
276*	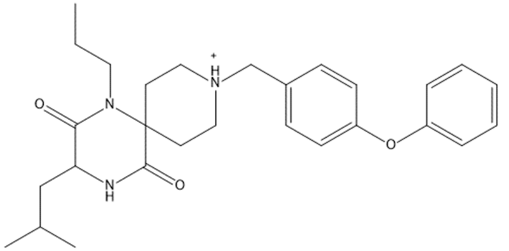	CXCR4	3ODU
277*	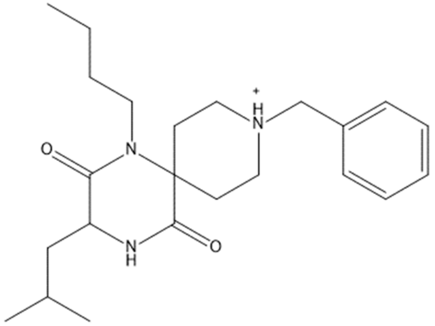	CXCR4	3ODU

278*	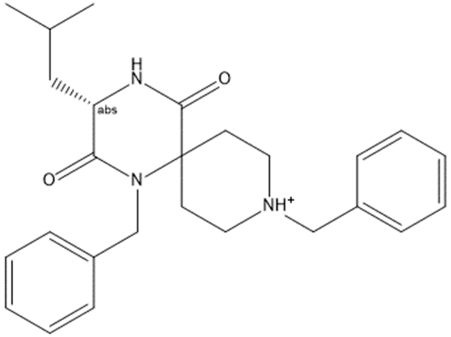	CXCR4	3ODU
279*	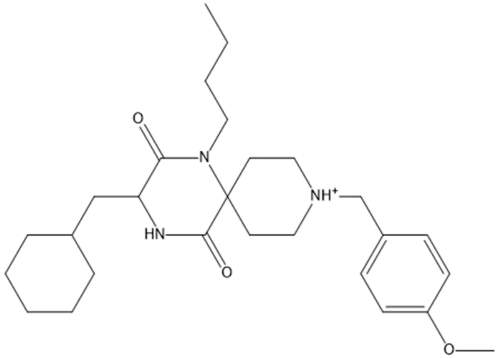	CXCR4	3ODU
280	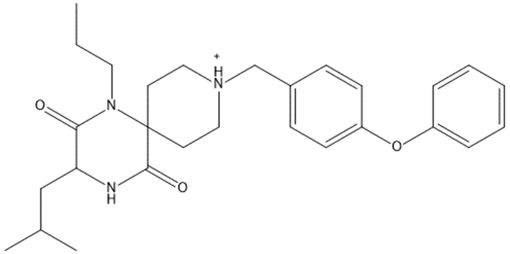	CXCR4	3ODU
281*	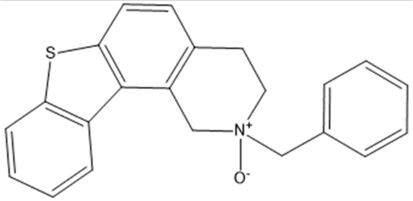	DRD3	3PBL

282*	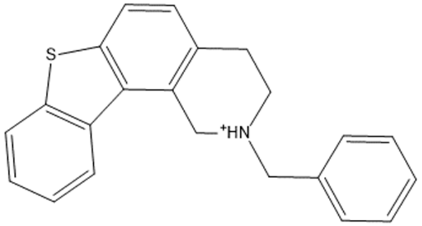	DRD3	3PBL
283*	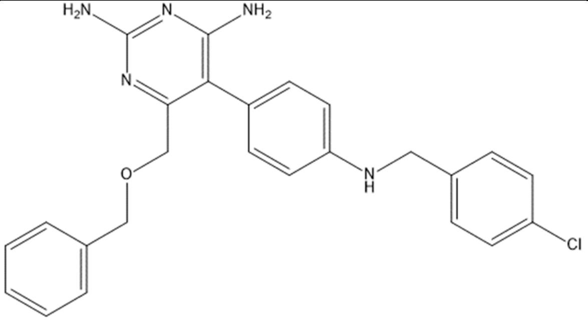	DRD3	3PBL
284*	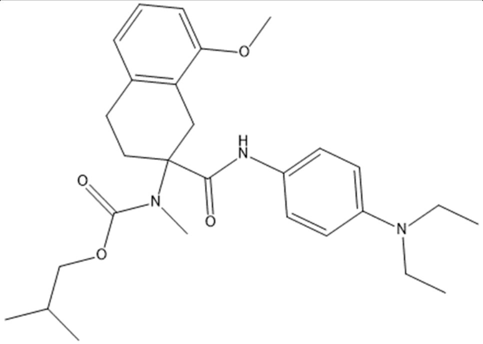	DRD3	3PBL
285*	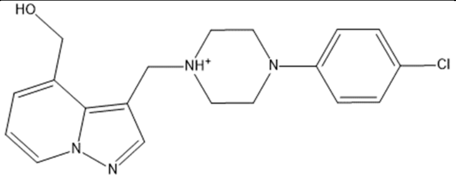	DRD3	3PBL

Entries marked with an asterisk denote ligands selected with MOE's Diverse Subset tool that were docked into modeled structures of their target.
[a]Structures are shown in the ionization state they were docked in. Additionally, structures labeled with 'Abs' denote the presence of a constrained stereocenter during docking. Stereocenters lacking this label were left unconstrained during docking.

Table S7. Known active ligands for 5 DUD-E GPCR targets.				
Ligand Number	Structure[a]	Ligand Name	Ligand Function	Target
1	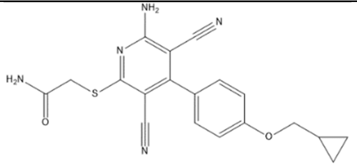	BAY 60-6583	Agonist	AA2AR
2	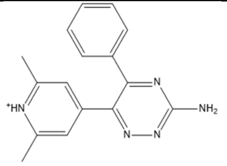	compound 4g [PMID: 22220592]	Agonist	AA2AR
3	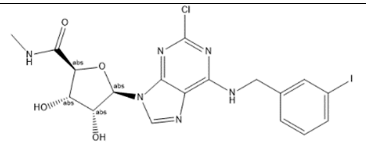	Cl-IB-MECA	Agonist	AA2AR
4	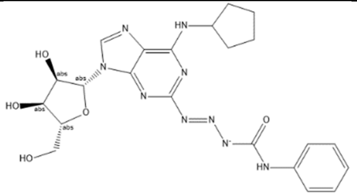	TCPA	Agonist	AA2AR
5	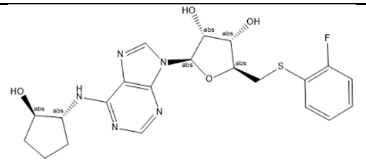	GS9667	Agonist	AA2AR
6	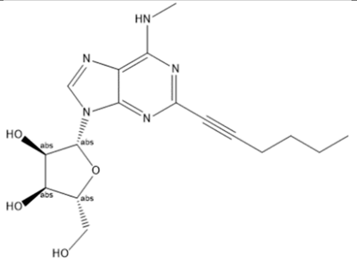	HEMADO	Agonist	AA2AR
7	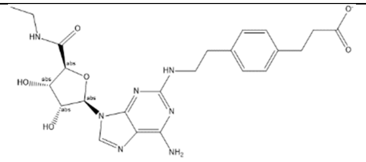	CGS 21680	Agonist	AA2AR

8	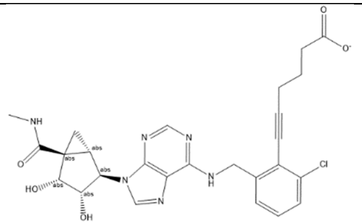	MRS5151	Agonist	AA2AR
9	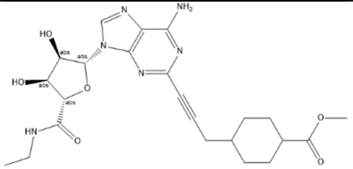	apadenoson	Agonist	AA2AR
10	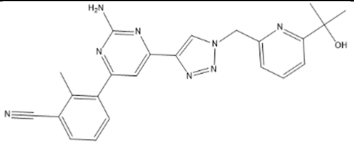	AB928	Antagonist	AA2AR
11	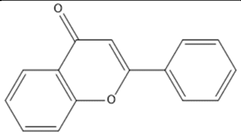	flavone	Antagonist	AA2AR
12	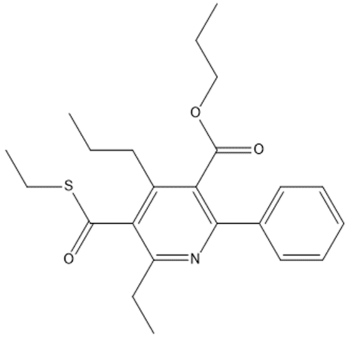	MRS1523	Antagonist	AA2AR
13	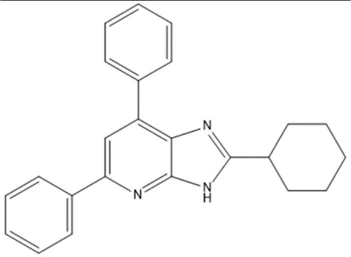	LUF5981	Antagonist	AA2AR

14	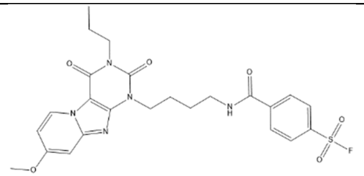	LUF7602	Antagonist	AA2AR
15	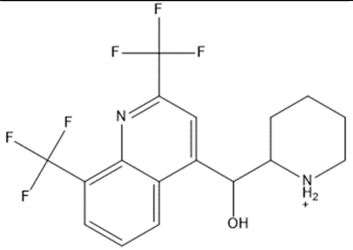	mefloquine	Antagonist	AA2AR
16	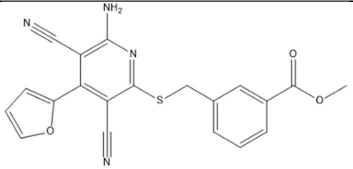	compound 10 [PMID: 31306001]	Antagonist	AA2AR
17	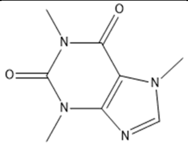	caffeine	Antagonist	AA2AR
18	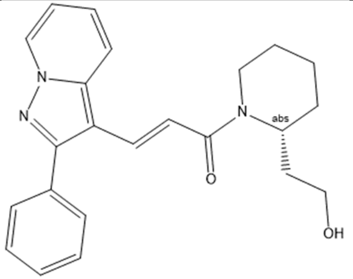	FK-453	Antagonist	AA2AR
19	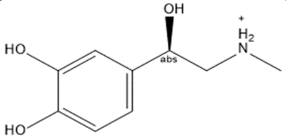	(-)-adrenaline	Agonist	ADRB1
20	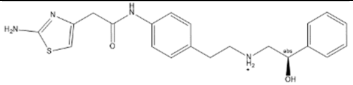	mirabegron	Agonist	ADRB1
21	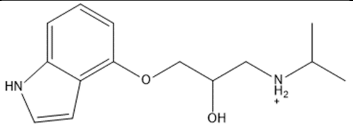	pindolol	Agonist	ADRB1

22	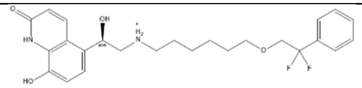	abediterol	Agonist	ADRB1
23	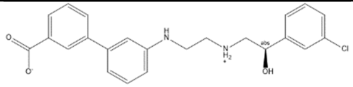	solabegron	Agonist	ADRB1
24	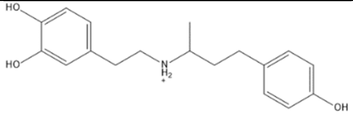	dobutamine	Agonist	ADRB1
25	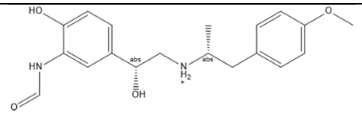	arformoterol	Agonist	ADRB1
26	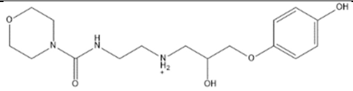	xamoterol	Agonist	ADRB1
27	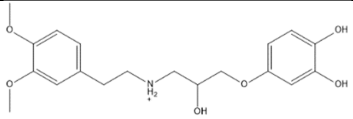	(-)-Ro 363	Agonist	ADRB1
28	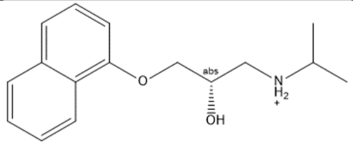	(-)-propranolol	Antagonist	ADRB1
29	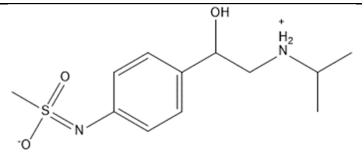	sotalol	Antagonist	ADRB1
30	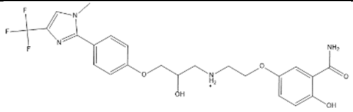	CGP 20712A	Antagonist	ADRB1
31	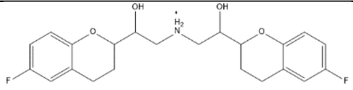	nebivolol	Antagonist	ADRB1
32	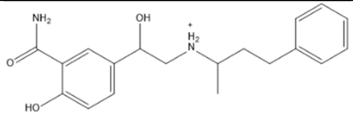	labetalol	Antagonist	ADRB1
33	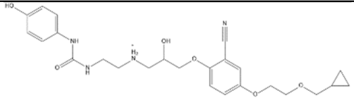	LK 204-545	Antagonist	ADRB1

34	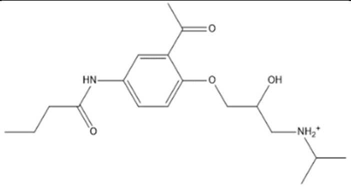	acebutolol	Antagonist	ADRB1
35	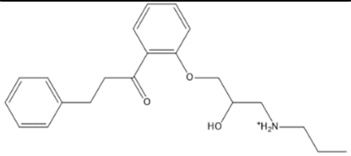	propafenone	Antagonist	ADRB1
36	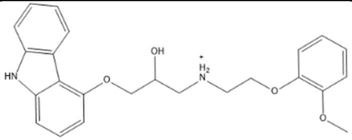	carvedilol	Antagonist	ADRB1
37	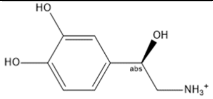	(-)-noradrenaline	Agonist	ADRB2
38	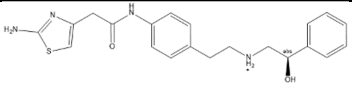	mirabegron	Agonist	ADRB2
39	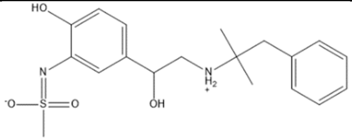	zinterol	Agonist	ADRB2
40	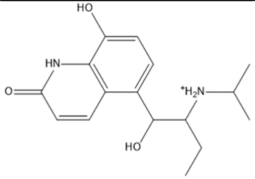	procaterol	Agonist	ADRB2
41	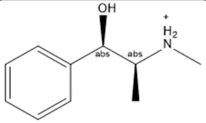	ephedrine	Agonist	ADRB2
42	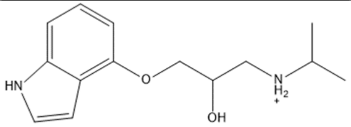	pindolol	Agonist	ADRB2
43	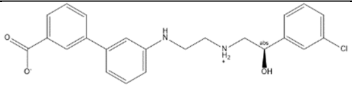	solabegron	Agonist	ADRB2

44	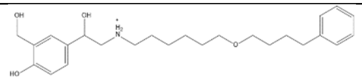	salmeterol	Agonist	ADRB2
45	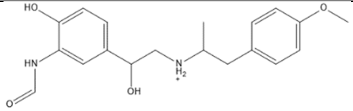	formoterol	Agonist	ADRB2
46	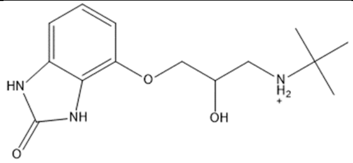	CGP 12177	Antagonist	ADRB2
47	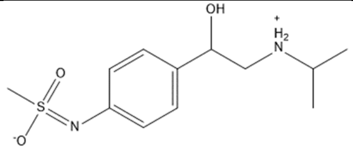	sotalol	Antagonist	ADRB2
48	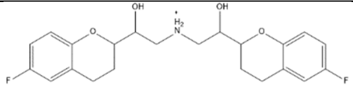	nebivolol	Antagonist	ADRB2
49	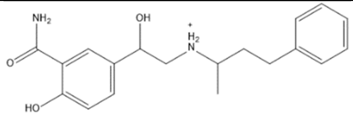	labetalol	Antagonist	ADRB2
50	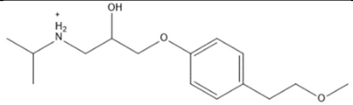	metoprolol	Antagonist	ADRB2
51	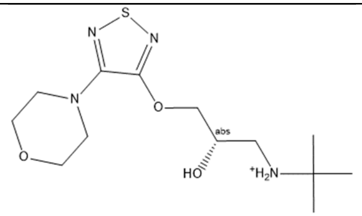	timolol	Antagonist	ADRB2
52	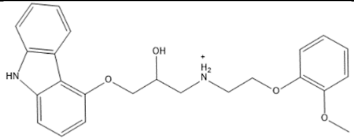	carvedilol	Antagonist	ADRB2
53	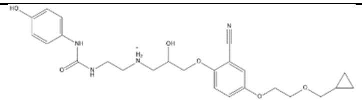	LK 204-545	Antagonist	ADRB2

54	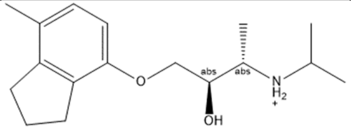	ICI 118551	Inverse agonist	ADRB2
55	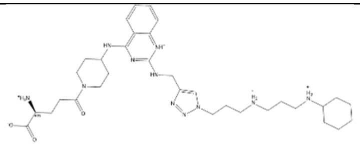	CX549	Antagonist	CXCR4
56	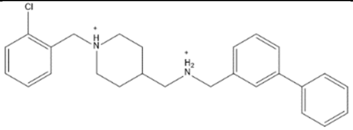	CXCR4 antagonist 22	Antagonist	CXCR4
57	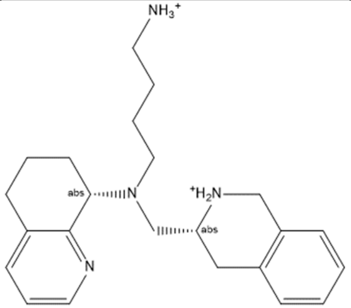	TIQ-15	Antagonist	CXCR4
58	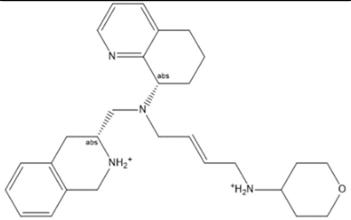	compound 46c [PMID: 29350534]	Antagonist	CXCR4
59	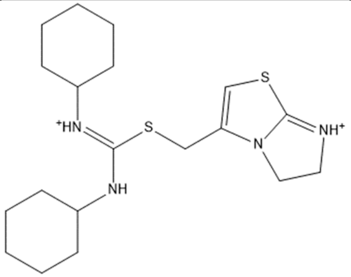	isothiourea-1a	Antagonist	CXCR4

60	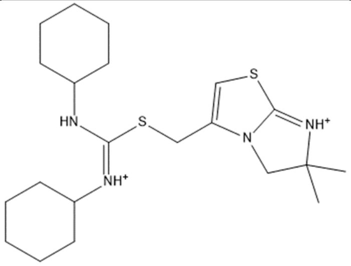	isothiourea-1t	Antagonist	CXCR4
61	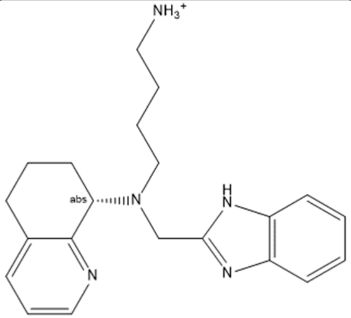	mavorixafor	Antagonist	CXCR4
62	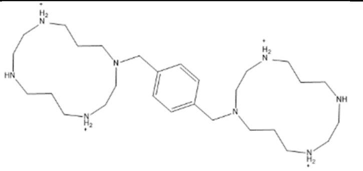	plerixafor	Antagonist	CXCR4
63	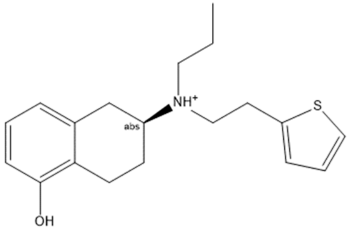	rotigotine	Agonist	DRD3
64	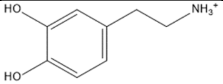	dopamine	Agonist	DRD3
65	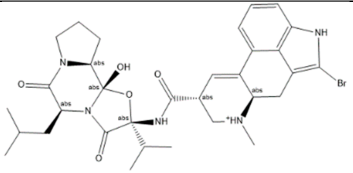	bromocriptine	Agonist	DRD3

66	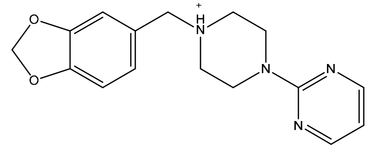	piribedil	Agonist	DRD3
67	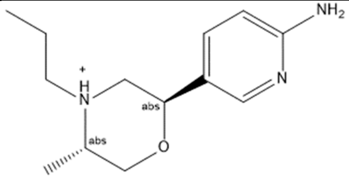	PF-592379	Agonist	DRD3
68	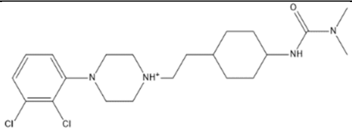	cariprazine	Agonist	DRD3
69	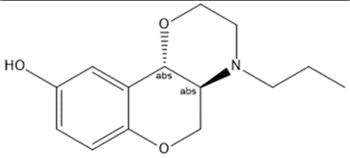	PD 128907	Agonist	DRD3
70	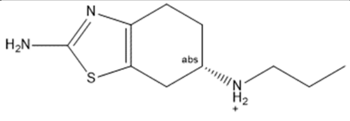	pramipexole	Agonist	DRD3
71	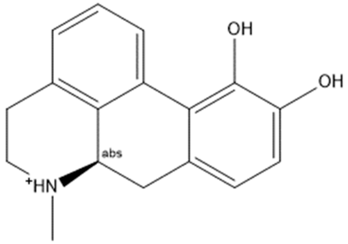	apomorphine	Agonist	DRD3
72	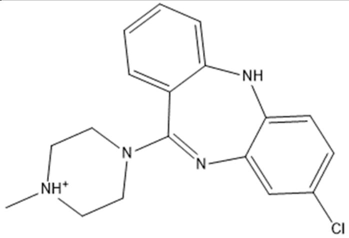	clozapine	Antagonist	DRD3

73	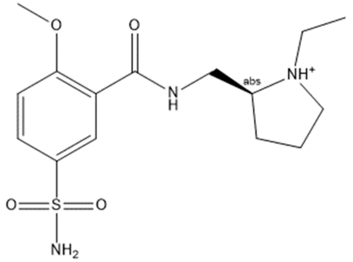	(-)-sulpiride	Antagonist	DRD3
74	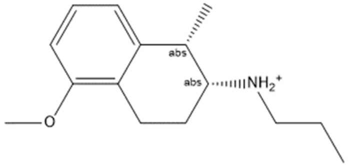	(+)-AJ76	Antagonist	DRD3
75	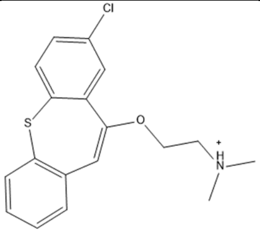	zotepine	Antagonist	DRD3
76	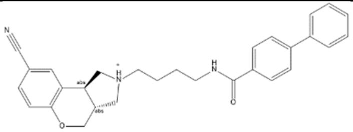	S33084	Antagonist	DRD3
77	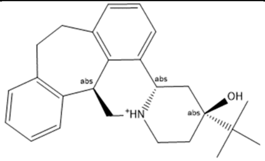	(+)-butaclamol	Antagonist	DRD3
78	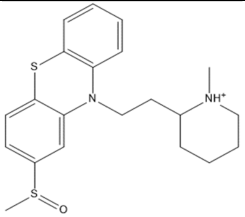	mesoridazine	Antagonist	DRD3

79	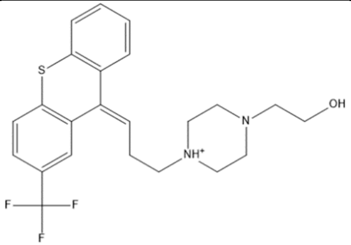	flupentixol	Antagonist	DRD3
80	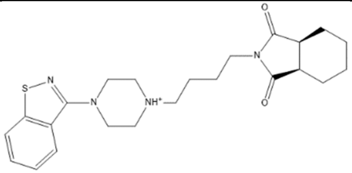	perospirone	Antagonist	DRD3

[a]Structures are shown in the ionization state they were docked in. Additionally, structures labeled with 'Abs' denote the presence of a constrained stereocenter during docking. Stereocenters lacking this label were left unconstrained during docking.

Table S8. Global sequence similarity comparison between external dataset GPCR targets and internal dataset DUD-E GPCR targets.

	Global Sequence Similarity (%)[a]	
DUD-E GPCR Target	GPR31	TAAR2
AA2AR	2.2	3.6
ADRB1	12.8	33.4
ADRB2	8.4	31.9
CXCR4	10.5	11.6
DRD3	12.5	26.2

[a] Global unweighted sequence similarity calculated by Ngo et al.

Table S9. Ligands docked into GPR31 and TAAR2 homology models.

Ligand Number	Structure[a]	Ligand Name	Function	Target
1	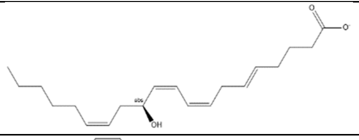	12(S)-HETE	Agonist	GPR31
2	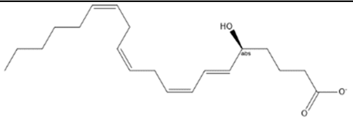	5(S)-HETE	Agonist	GPR31
3	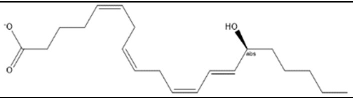	15(S)-HETE	Agonist	GPR31
4	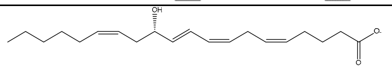	12(R)-HETE	Inactive	GPR31
5	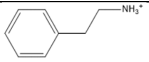	beta phenylethamine	Agonist	TAAR2
6	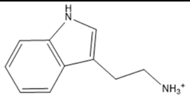	tryptamine	Agonist	TAAR2

[a]Structures are shown in the ionization state they were docked in.

Table S10. Ballesteros-Weinstein numbered GPCR residue positions whose interaction energies and types were used as predictors for the random forest classifier, categorized by transmembrane (TM) domain.

**TM1**	**TM2**	**TM3**	**TM4**	**TM5**	**TM6**	**TM7**
1.31	2.53	3.25	4.55	5.31	6.44	7.31
1.35	2.54	3.26	4.56	5.35	6.48	7.32
1.39	2.56	3.28	4.57	5.36	6.51	7.34
1.42	2.57	3.29	4.59	5.37	6.52	7.35
	2.58	3.30	4.60	5.38	6.54	7.36
	2.60	3.32	4.61	5.39	6.55	7.38
	2.61	3.33		5.40	6.58	7.39
	2.63	3.35		5.41	6.59	7.40
	2.64	3.36		5.42	6.62	7.42
	2.65	3.37		5.43		7.43
		3.40		5.46		7.46
				5.47		

Table S11. Active ligands extracted from experimental structures for self- and cross-docking.

Ligand Number	Structure[a]	Ligand Name	Target(s)[a]	Activity	Self-dock PDBID(s)	Cross-dock PDBID(s)
1	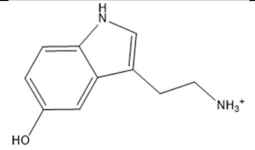	5-hydroxytryptamine	1. 5HT1A<br>2. 5HT1D	1. Agonist<br>2. Agonist	1. 7E2Y<br>2. 7E32	1. 7E2X
2	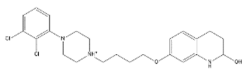	aripiprazole	1. 5HT1A<br>2. 5HT2A	1. Agonist<br>2. Agonist	1. 7E2Z<br>2. 7VIE	1. 7E2X
3	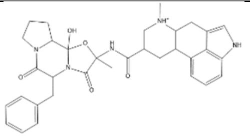	dihydroergotamine	5HT1B	Agonist	4IAQ	4IAR
4	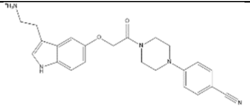	donitriptan	5HT1B	Agonist	6G79	4IAR
5	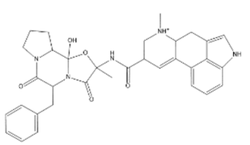	ergotamine	1. 5HT1B<br>2. 5HT2B<br>3. 5HT2C	1. Agonist<br>2. Agonist<br>3. Agonist	1. 4IAR, 7C61<br>2. 4IB4, 4NC3, 5TUD<br>3. 6BQG	1. 4IAQ, 5V54<br>2. 4NC3, 4IB4, 4IB4<br>3. 6BQH
6	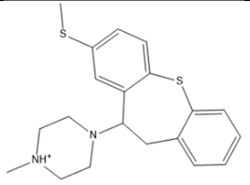	CHEMBL428892	1. 5HT1B<br>2. 5HT2A	1. Antagonist<br>2. Inverse agonist	1. 5V54<br>2. 6WH4	1. 7C61<br>2. 7WC8
7	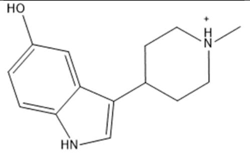	BRL-54443	5HT1E	Agonist	7E33	NA[b]
8	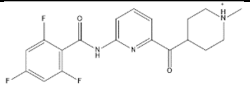	lasmiditan	5HT1F	Agonist	7EXD	NA
9	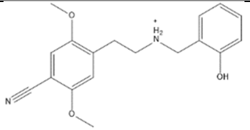	25-CN-NBOH	5HT2A	Agonist	6WHA	7WC8
10	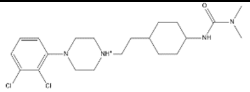	cariprazine	5HT2A	Agonist	7VOD	7VOE

11	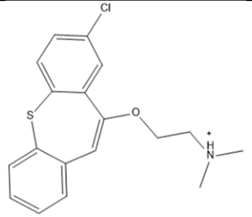	zotepine	5HT2A	Antagonist	6A94	6A93
12	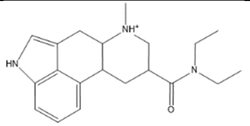	lysergide	1. 5HT2A<br>2. 5HT2B	1. Agonist<br>2. Agonist	1. 6WGT<br>2. 5TVN	1. 7WC8<br>2. 4IB4
13	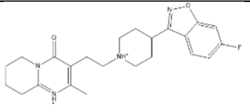	risperidone	1. 5HT2A<br>2. DRD2	1. Antagonist<br>2. Inverse agonist	1. 6A93<br>2. 6CM4	1. 6A94<br>2. 6LUQ
14	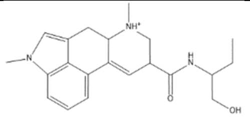	methysergide	5HT2B	Antagonist	6DRZ	6DRX
15	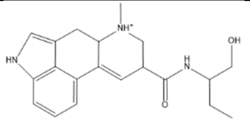	methylergonovine	5HT2B	Agonist	6DRY	4IB4
16	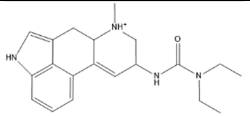	lisuride	5HT2B	Antagonist	6DRX	6DRZ
17	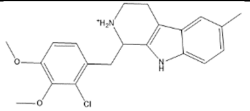	LY266097	5HT2B	Antagonist	6DS0	6DRX
18	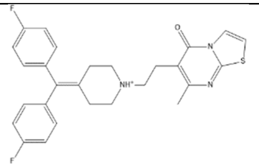	ritanserin	5HT2C	Inverse agonist	6BQH	6BQG
19	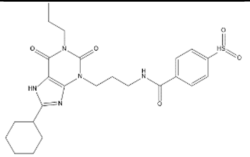	DU172	AA1R	Antagonist	5UEN	5N2S

20	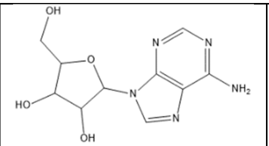	adenosine	1. AA1R<br>2. AA2AR	1. Agonist<br>2. Agonist	1. 6D9H, 7LD3, 7LD4<br>2. 2YDO	1. 7LD3, 7LD4<br>2. 5WF5
21	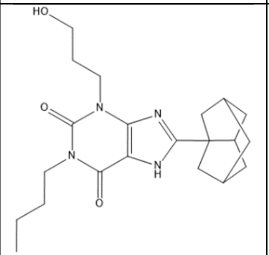	PSB36	1. AA1R<br>2. AA2AR	1. Agonist<br>2. Antagonist	1. 5N2S<br>2. 5N2R	1. 5UEN<br>2. 5NM4
22	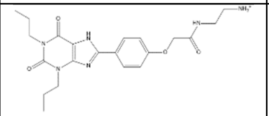	xanthine amine congener	AA2AR	Antagonist	3REY	5NM4
23	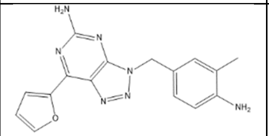	vipadenant	AA2AR	Antagonist	5OLH	5NM4
24	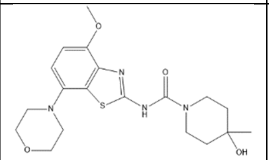	tozadenant	AA2AR	Antagonist	5OLO	5NM4
25	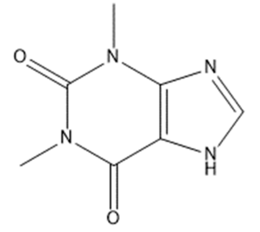	theophylline	AA2AR	Antagonist	5MZJ	5NM4
26	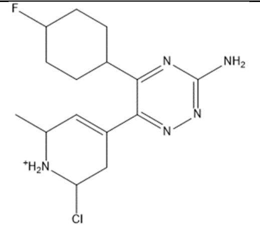	imaradenant	AA2AR	Antagonist	6GT3	5NM4

27	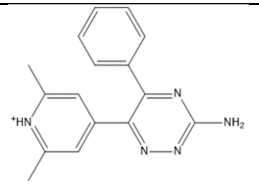	compound 4g [PMID: 22220592]	AA2AR	Antagonist	3UZA	5NM4
28	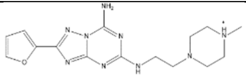	CHEMBL3934661	AA2AR	Antagonist	5IU8	5NM4
29	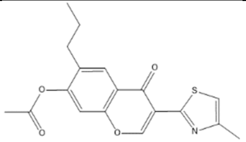	CHEMBL2030687	AA2AR	Antagonist	6ZDR	5NM4
30	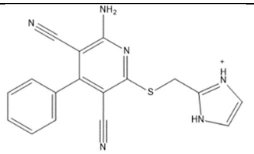	CHEMBL124345	AA2AR	Agonist	7ARO	5NM4
31	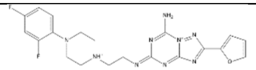	CHEMBL184061	AA2AR	Antagonist	5IUB	5NM4
32	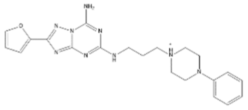	2-(furan-2-yl)-5-N-[3-(4-phenylpiperazin-1-yl)propyl]-1H-[1,2,4]triazolo[1,5-a][1,3,5]triazin-8-ium-5,7-diamine	AA2AR	Antagonist	5IUA	5NM4
33	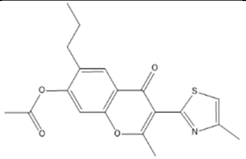	2-methyl-3-(4-methylthiazol-2-yl)-4-oxo-6-propyl-4H-chromen-7-yl acetate	AA2AR	Antagonist	6ZDV	5NM4
34	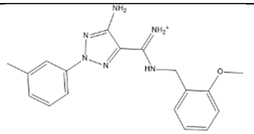	5-amino-N-[(2-methoxyphenyl)methyl]-2-(3-methylphenyl)-2h-1,2,3-triazole-4-carboximidamide	AA2AR	Antagonist	5UIG	5NM4
35	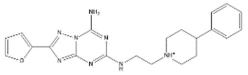	2-(furan-2-yl)-5-N-[2-(4-phenylpiperidin-1-yl)ethyl]-1H-[1,2,4]triazolo[1,5-a][1,3,5]triazin-8-ium-5,7-diamine	AA2AR	Antagonist	5IU7	5NM4
36	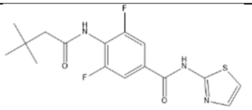	CHEMBL1671936	AA2AR	Antagonist	5OLV	5NM4
37	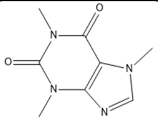	caffeine	AA2AR	Antagonist	3RFM, 5MZP	5NM4

38	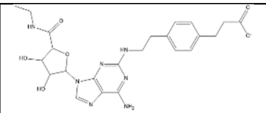	CGS 21680	AA2AR	Agonist	4UG2, 4UHR	5WF5
39	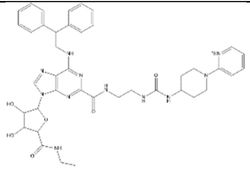	UK-432,097	AA2AR	Agonist	3QAK, 5WF5, 5WF6	5WF5, 4UHR
40	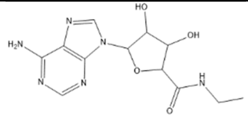	NECA	AA2AR	Agonist	2YDV, 5G53, 6GDG	5WF5
41	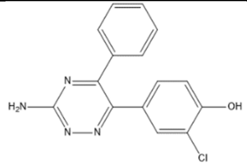	CHEMBL2024114	AA2AR	Antagonist	3UZC, 5OLZ, 5OM1, 5OM4	5NM4
42	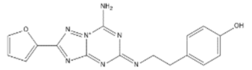	ZM-241385	AA2AR	Antagonist	3EML, 3PWH, 3VG9, 3VGA, 4EIY, 5IU4, 5JTB, 5K2A, 5K2B, 5K2C, 5K2D, 5NLX, 5NM2, 5NM4, 5OLG, 5UVI, 5VRA, 6AQF, 6JZH, 6LPJ, 6LPK, 6LPL, 6MH8, 6PS7, 6S0L, 6S0Q, 6WQA, 7RM5	5NM4, 5IU4
43	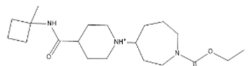	ethyl (4S)-4-[4-[(1-methylcyclobutyl)carbamoyl]piperidin-1-yl]azepane-1-carboxylate	ACM1	Agonist	6ZG4	6ZFZ
44	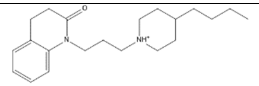	CHEMBL3354065	ACM1	Agonist	6ZFZ	6ZG4
45	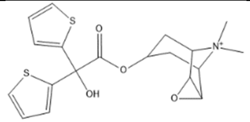	0HK	ACM1	Antagonist	5CXV	6WJC

46	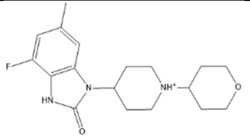	7-fluoro-5-methyl-3-[1-(oxan-4-yl)piperidin-4-yl]-1H-benzimidazol-2-one	ACM1	Agonist	6ZG9	6ZFZ
47	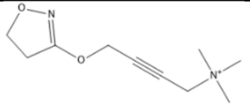	iperoxo	1. ACM1<br>2. ACM2	1. Agonist<br>2. Agonist	1. 6OIJ<br>2. 4MQS	1. 6ZFZ<br>2. 6OIK
48	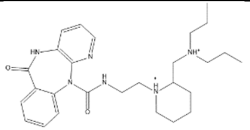	AF-DX 384	ACM2	Antagonist	5ZKB	5ZKC
49	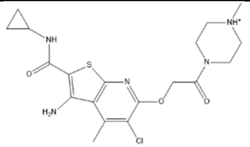	LY2119620	ACM2	Agonist	6U1N	4MQS
50	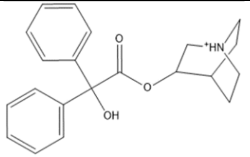	CHEMBL558910	ACM2	Antagonist	3UON, 5ZK3	5ZKC
51	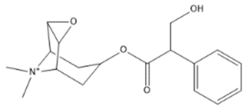	N-methyl scopolamine	1. ACM2<br>2. ACM3	1. Antagonist<br>2. Antagonist	1. 5YC8, 5ZK8, 5ZKC<br>2. 4U16	1. 5ZKC, 5YC8<br>2. 4U15
52	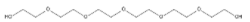	(1R,2R,4S,5S,7s)-7-({[4-fluoro-2-(thiophen-2-yl)phenyl]carbamoyl}oxy)-9,9-dimethyl-3-oxa-9-azatricyclo[3.3.1.0~2,4~]nonan-9-ium	ACM3	Antagonist	5ZHP	4U15
53	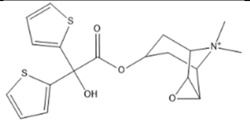	tiotropium	1. ACM3<br>2. ACM4<br>3. ACM5	1. Antagonist<br>2. Antagonist<br>3. Inverse agonist	1. 4DAJ, 4U14<br>2. 5DSG<br>3. 6OL9	1. 4U15<br>2. 6KP6
54	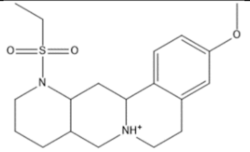	RSC	ADA2A	Antagonist	6KUX	6KUY

55	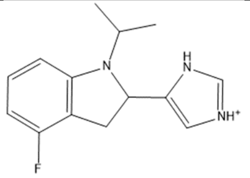	(2~{S})-4-fluoranyl-2-(1~{H}-imidazol-5-yl)-1-propan-2-yl-2,3-dihydroindole	ADA2A	Agonist	6KUY	6KUX
56	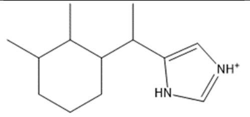	dexmedetomidine	ADA2B	Agonist	6K41, 6K42	6K42, 6K41
57	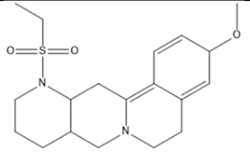	(8aR,12aS,13aR)-12-ethylsulfonyl-3-methoxy-5,6,8,8a,9,10,11,12a,13,13a-decahydroisoquinolino[2,1-g][1,6]naphthyridine	ADA2C	Antagonist	6KUW	NA
58	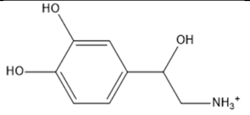	noradrenaline	ADRB1	Agonist	7BU6	6H7N
59	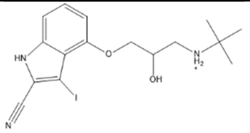	iodocyanopindolol	ADRB1	Antagonist	2YCZ	4BVN
60	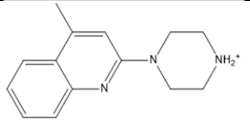	CHEMBL1559535	ADRB1	Antagonist	3ZPR	4BVN
61	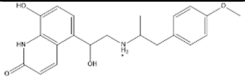	carmoterol	ADRB1	Agonist	2Y02	2Y03
62	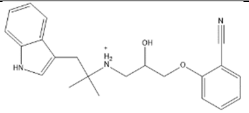	bucindolol	ADRB1	Agonist	4AMI	2Y02
63	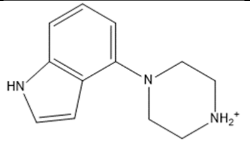	CHEMBL200234	ADRB1	Antagonist	3ZPQ	4BVN
64	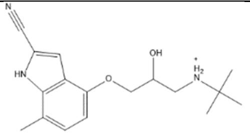	7-methylcyanopindolol	ADRB1	Inverse agonist	5A8E	4AMJ
65	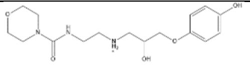	(S)-xamoterol	ADRB1	Agonist	6H7N	7BU7

66	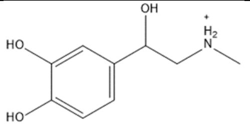	(-)-adrenaline	ADRB1	Agonist	7BTS	6H7N
67	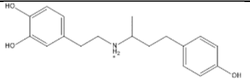	dobutamine	ADRB1	Agonist	2Y00, 2Y01, 6H7L	2Y01, 2Y00, 6H7N
68	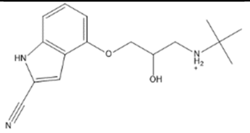	(S)-cyanopindolol	ADRB1	Antagonist	2VT4, 2YCY, 4BVN, 5F8U, 6H7O	4BVN, 4BVN, 3ZPR, 4BVN, 6H7N
69	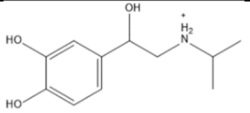	levisoprenaline	1. ADRB1<br>2. ADRB2	1. Agonist<br>2. Agonist	1. 2Y03, 6H7J, 7JJO,<br>2. 7DHR	1. 2Y02, 6H7N, 6H7N<br>2. 4LDE
70	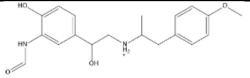	arformoterol	1. ADRB1<br>2. ADRB2	1. Agonist<br>2. Agonist	1. 6IBL, 6TKO<br>2. 7BZ2	1. 6H7N<br>2. 4LDE
71	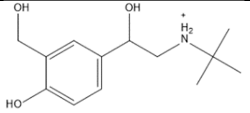	levosalbutamol	1 ADRB1<br>2. ADRB2	1. Agonist<br>2. Agonist	1. 2Y04, 6H7M<br>2. 7DHI	1. 2Y00, 6H7N<br>2. 4LDE
72	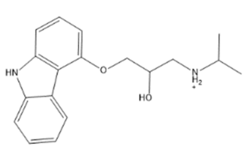	carazolol	1. ADRB1<br>2. ADRB2	1. Antagonist<br>2. Inverse agonist	1. 2YCW, 7BVQ<br>2. 2RH1, 4GBR, 5D5A, 5D5B, 5D6L, 5JQH	1. 4BVN, 4AMJ<br>2. 5D5A, 2RH1
73	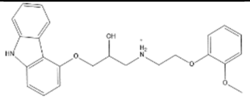	CHEMBL3799125	ADRB1, ADRB2	Inverse agonist, Antagonist	4AMJ, 6PS3	5A8E, 6PS2
74	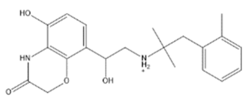	BI-167107	ADRB1, ADRB2, ADRB2, ADRB2, ADRB2, ADRB2	1. Agonist<br>2. Agonist	1. 7BU7, 3P0G, 3SN6<br>2. 4LDE, 6E67, 6NI3	1. 6H7N<br>2. 4LDE, 6MXT
75	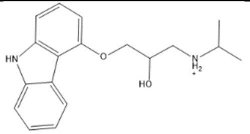	carazolol	ADRB2	Antagonist	6PS0	6PS2
76	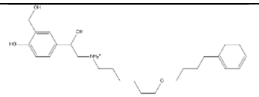	salmeterol	ADRB2	Agonist	6MXT	4LDE

77	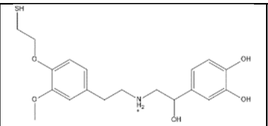	Q27453560	ADRB2	Agonist	4QKX	4LDE
78	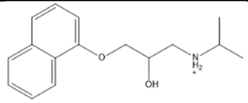	propranolol	ADRB2	Antagonist	6PS5	6PS2
79	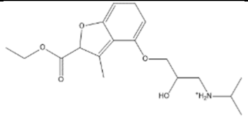	CHEMBL1233771	ADRB2	Inverse agonist	3NY9	2RH1
80	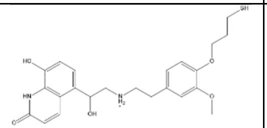	FAUC50	ADRB2	Agonist	3PDS	6PS2
81	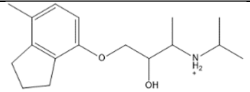	CHEMBL1233766	ADRB2	Antagonist	3NY8, 6PS4	2RH1, 6PS2
82	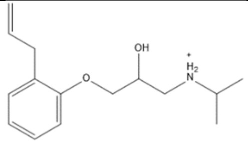	CHEMBL1160734	ADRB2	Antagonist	3NYA, 6PRZ, 6PS2	6PS2, 6PS2, 6PS3
83	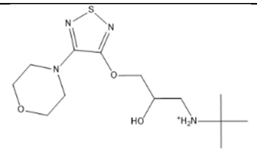	timolol	ADRB2	Antagonist	3D4S, 6PS1, 6PS6	2RH1, 6PS2, 6PS2
84	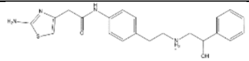	mirabegron	ADRB3	Agonist	7DH5	
85	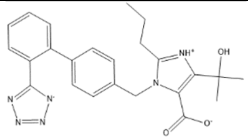	olmesartan	AGTR1	Inverse agonist	4ZUD	4YAY
86	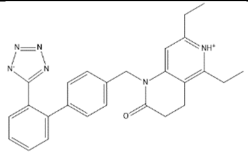	ZD-7155	AGTR1	Antagonist	4YAY	4ZUD
87	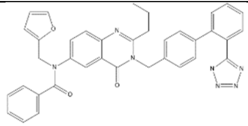	compound 2 [PMID: 28379944]	AGTR2	Antagonist	5UNH	5UNF

88	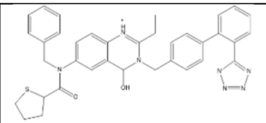	compound 1 [PMID: 28379944]	AGTR2	Antagonist	5UNF, 5UNG	5UNG, 5UNF
89	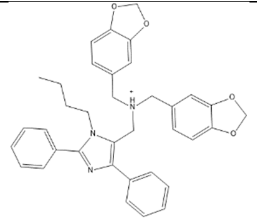	NDT9513727	C5AR1	Antagonist	5O9H	6C1R
90	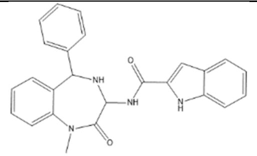	devazepide	CCKAR	Antagonist	7F8Y	7F8U
91	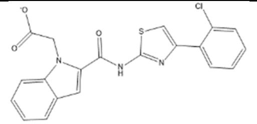	lintitript	CCKAR	Antagonist	7F8U	7F8Y
92	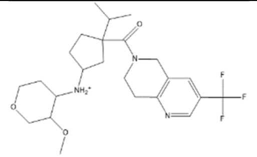	MK-0812	CCR2	Antagonist	6GPS, 6GPX	6GPX, 6GPS
93	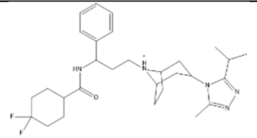	maraviroc	CCR5	Antagonist	4MBS	5UIW
94	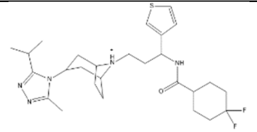	CCR5 antagonist 34	CCR5	Antagonist	6AKY	5UIW
95	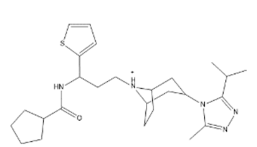	N-[(1S)-3-{(3-exo)-3-[3-methyl-5-(propan-2-yl)-4H-1,2,4-triazol-4-yl]-8-azabicyclo[3.2.1]octan-8-yl}-1-(thiophen-2-yl)propyl]cyclopentanecarboxamide	CCR5	Antagonist	6AKX	5UIW
96	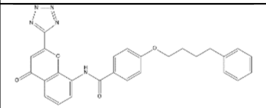	pranlukast	CLTR1	Antagonist	6RZ4	6RZ5

97	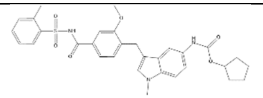	zafirlukast	CLTR1	Antagonist	6RZ5	6RZ4
98	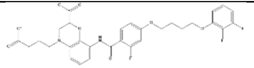	ONO-2080365	CLTR2	Antagonist	6RZ8	6RZ6
99	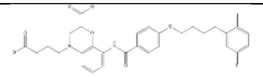	ONO-2770372	CLTR2	Antagonist	6RZ9	6RZ6
100	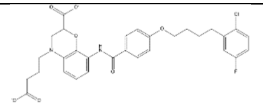	ONO-2570366	CLTR2	Antagonist	6RZ6, 6RZ7	6RZ7, 6RZ6
101	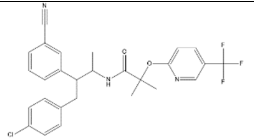	taranabant	CNR1	Inverse agonist	5U09	5TGZ
102	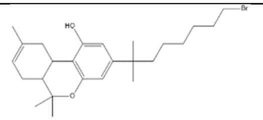	AM11542	CNR1	Agonist	5XRA	5XR8
103	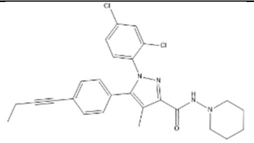	AM6538	CNR1	Antagonist	5TGZ	5U09
104	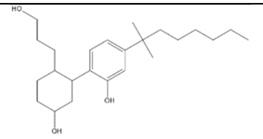	CP55940	CNR1	Agonist	7V3Z	5XRA
105	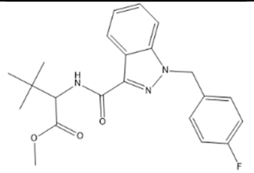	MDMB-fubinaca	CNR1	Agonist	6N4B	5XRA
106	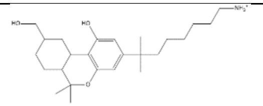	AM841	CNR1, CNR1, CNR2, CNR2	1. Agonist<br>2. Agonist	1. 5XR8, 6KPG<br>2. 6KPC, 6KPF	1. 5XRA, 5XRA<br>2. 5ZTY, 6PT0
107	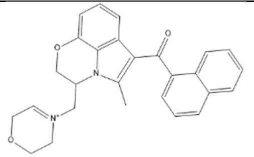	WIN55212-2	CNR2	Agonist	6PT0	6KPF

108	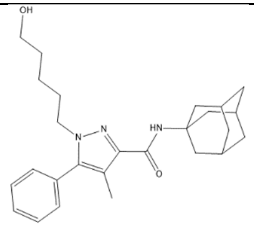	AM10257	CNR2	Antagonist	5ZTY	6KPC
109	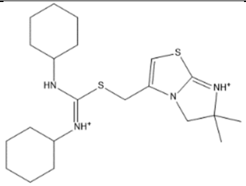	isothiourea-1t	CXCR4	Antagonist	3ODU, 3OE6, 3OE8, 3OE9	3OE0, 3ODU
110	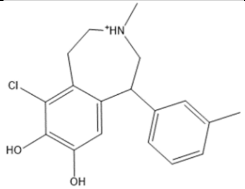	39YLC3L0ZU	DRD1	Agonist	7JVP	7JVQ
111	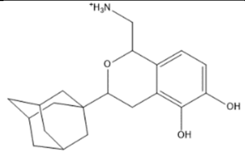	A77636	DRD1	Agonist	7CKX	7JVP
112	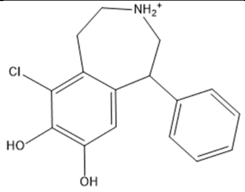	CHEMBL1160787	DRD1	Agonist	7JV5	7JVP
113	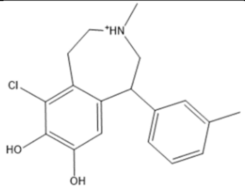	CHEMBL1416789	DRD1	Agonist	7CRH	7JVP
114	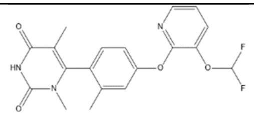	CHEMBL3697578	DRD1	Agonist	7CKY	7JVP
115	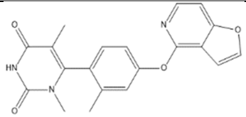	CHEMBL4453318	DRD1	Agonist	7JOZ	7JVP

116	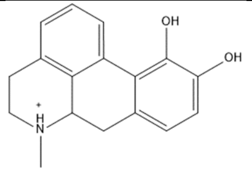	apomorphine	DRD1	Agonist	7JVQ	7JVP
117	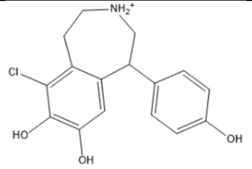	fenoldopam	DRD1	Agonist	7CKW	7JVP
118	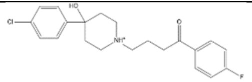	haloperidol	DRD2	Antagonist	6LUQ	7DFP
119	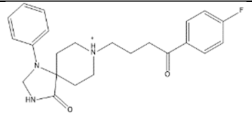	spiperone	DRD2	Antagonist	7DFP	6LUQ
120	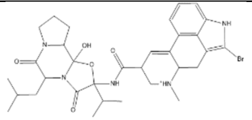	bromocriptine	DRD2	Agonist	6VMS, 7JVR	7JVR, 6VMS
121	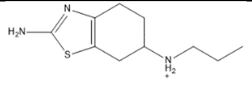	pramipexole	DRD3	Agonist	7CMU	7CMV
122	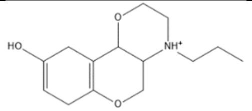	CHEMBL70565	DRD3	Agonist	7CMV	7CMU
123	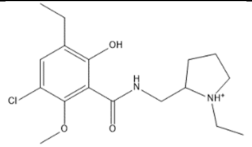	eticlopride	DRD3	Antagonist	3PBL	7CMV
124	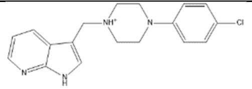	L745870	DRD4	Antagonist	6IQL	5WIU
125	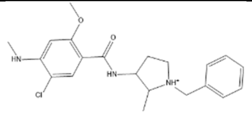	[3H]-nemonapride	DRD4	Antagonist	5WIU, 5WIV	5WIV, 5WIU
126	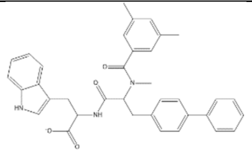	IRL 2500	EDNRB	Inverse agonist	6K1Q	6IGK

127	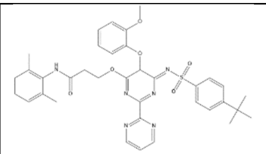	K-8794	EDNRB	Antagonist	5X93	6IGK
128	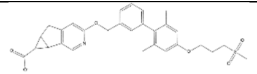	MK-8666	FFAR1	Agonist	5TZR	4PHU
129	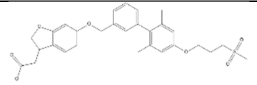	fasiglifam	FFAR1	Agonist	4PHU	5TZR
130	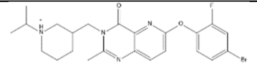	CHEMBL1956994	GHSR	Antagonist	6KO5	7F83
131	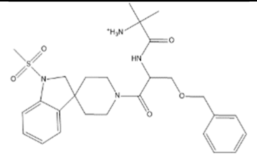	ibutamoren	GHSR	Agonist	7NA8	7NA7
132	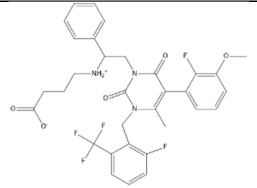	elagolix	GNRHR	Antagonist	7BR3	NA
133	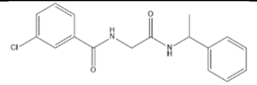	JNJ-63533054	GP139	Agonist	7VUG, 7VUH, 7VUI, 7VUJ	7VUH, 7VUG, 7VUG, 7VUG
134	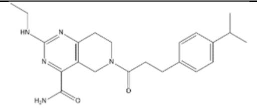	CHEMBL2331646	GPBAR	Agonist	7CFM	7CFN
135	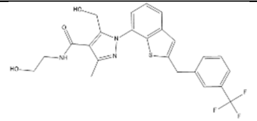	derivative 17 [Nakahata *et al.*, 2018]	GPR52	Agonist	6LI0	6LI2
136	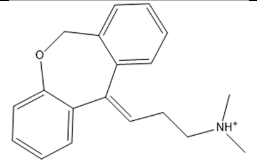	doxepin	HRH1	Antagonist	3RZE	7DFL
137	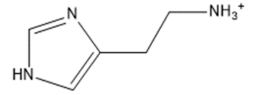	histamine	HRH1	Agonist	7DFL	3RZE

138	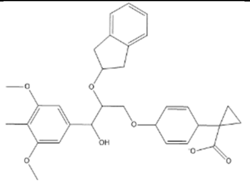	ONO-3080573	LPAR1	Antagonist	4Z36	4Z35
139	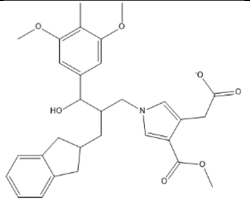	ONO-9780307	LPAR1	Antagonist	4Z34	4Z35
140	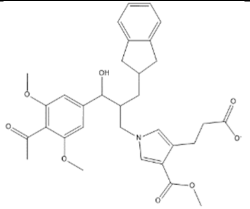	ONO-9910539	LPAR1	Antagonist	4Z35	4Z36
141	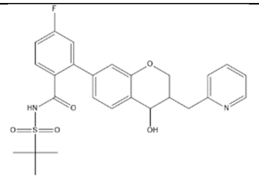	N-(tert-butylsulfonyl)-4-fluoro-2-{(3S,4R)-4-hydroxy-3-[(pyridin-2-yl)methyl]-3,4-dihydro-2H-1-benzopyran-7-yl}benzamide	LT4R1	Antagonist	7K15	7VKT
142	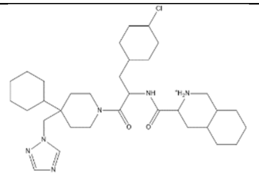	THIQ	MC4R	Agonist	7F58	7PIU
143	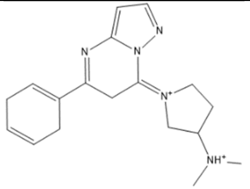	(R)-ZINC-3573	MRGX	Agonist	7S8N, 7S8O	7S8L, 7S8L
144	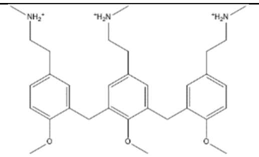	compound 48/80	MRGX2	Agonist	7VDH, 7VV6	7S8L, 7S8L

145	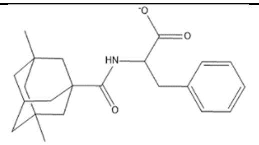	N-[(1R,3R,5S,7R)-3,5-dimethyltricyclo[3.3.1.1~3,7~]decane-1-carbonyl]-D-phenylalanine	MRGX4	Agonist	7S8P	
146	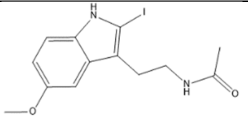	2-iodomelatonin	MTR1A	Agonist	6ME4	6ME2
147	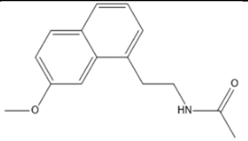	agomelatine	MTR1A	Agonist	6ME5	6ME2
148	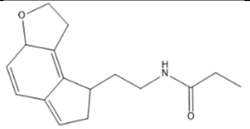	ramelteon	MTR1A,<br>MTR1A,<br>MTR1B	1. Agonist<br>2. Agonist	1. 6ME2, 7DB6<br>2. 6ME9	1. 6ME3, 7VGY<br>2. 6ME6
149	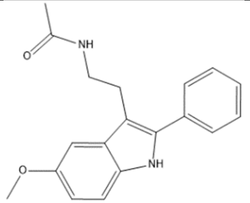	2-phenylmelatonin	MTR1A,<br>MTR1A,<br>MTR1B,<br>MTR1B,<br>MTR1B	1. Agonist<br>2. Agonist	1. 6ME3, 6PS8<br>2. 6ME6, 6ME7,<br>6ME8	1. 6ME2<br>2. 6ME8, 6ME6,
150	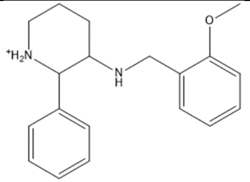	CP 99994	NK1R	Antagonist	6HLL	6HLP
151	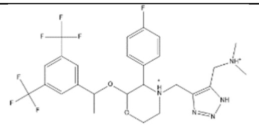	L760735	NK1R	Antagonist	6E59	6HLP
152	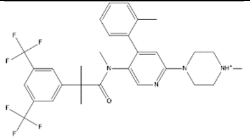	netupitant	NK1R	Antagonist	6HLP	6HLO
153	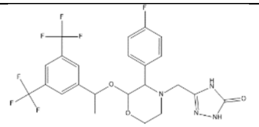	aprepitant	NK1R	Antagonist	6HLO, 6J20, 6J21	6HLP, 6HLP, 6HLP

154	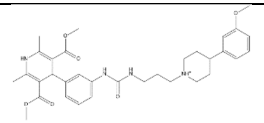	BMS-193885	NPY1R	Antagonist	5ZBH	5ZBQ
155	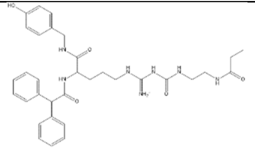	CHEMBL3747822	NPY1R	Antagonist	5ZBQ	5ZBH
156	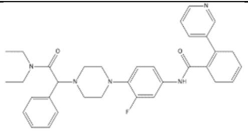	CHEMBL1823578	NPY2R	Antagonist	7DDZ	
157	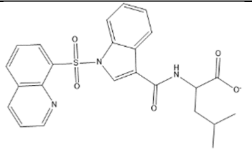	(2~{S})-4-methyl-2-[(1-quinolin-8-ylsulfonylindol-3-yl)carbonylamino]pentanoic acid	NTR1	Agonist	6Z8N	6Z4V
158	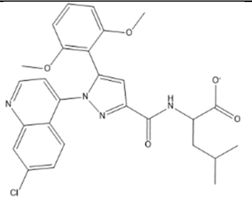	CHEMBL508044	NTR1	Agonist	6ZA8	6Z4V
159	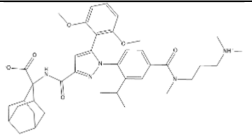	SR142948A	NTR1	Inverse agonist	6Z4Q	6ZIN
160	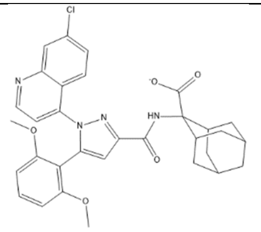	meclinertant	NTR1	Inverse agonist	6Z4S, 6ZIN	6ZIN, 6Z4S
161	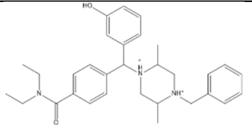	DPI-287	OPRD	Agonist	6PT3	6PT2

162	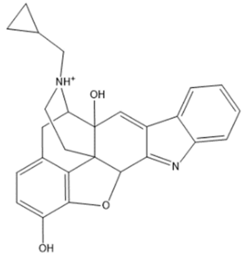	naltrindole	OPRD	Antagonist	4EJ4, 4N6H	4N6H, 4RWD
163	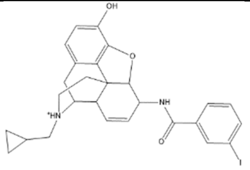	MP1104	OPRK	Agonist	6B73	4DJH
164	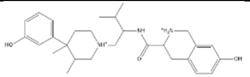	JDTic	OPRK	Antagonist	4DJH, 6VI4	6VI4, 4DJH
165	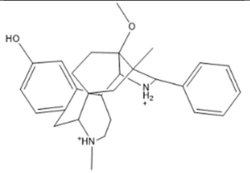	BU72	OPRM	Agonist	5C1M	7SBF
166	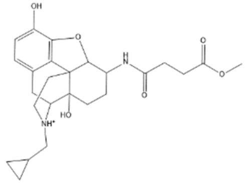	methyl 4-[[(4R,4aS,7R,7aR,12bS)-3-(cyclopropylmethyl)-4a,9-dihydroxy-1,2,4,5,6,7,7a,13-octahydro-4,12-methanobenzofuro[3,2-e]isoquinolin-7-yl]amino]-4-oxobutanoate	OPRM	Antagonist	4DKL	5C1M
167	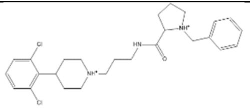	CHEMBL1783826	OPRX	Antagonist	5DHG	5DHH
168	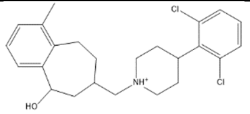	SB 612111	OPRX	Antagonist	5DHH	5DHG
169	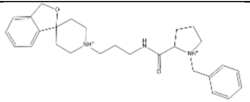	compound 24 [PMID: 16451050]	OPRX	Antagonist	4EA3	5DHG
170	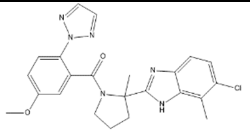	daridorexant	OX1R	Antagonist	6TP3	6TOD

171	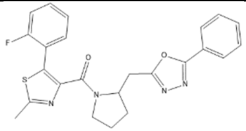	SB-674042	OX1R	Antagonist	4ZJC	6TOD
172	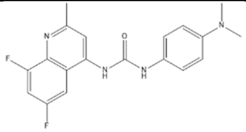	SB-408124	OX1R	Antagonist	6TQ9	6TOD
173	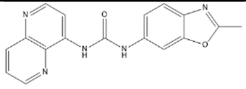	SB-334867	OX1R	Antagonist	6TQ7	6TOD
174	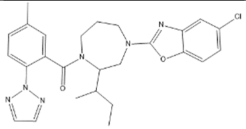	JH112	OX1R	Antagonist	6V9S	6TOD
175	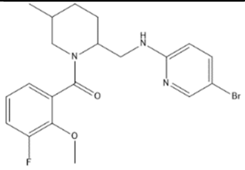	CHEMBL2413522	OX1R	Antagonist	6TOS	6TOD
176	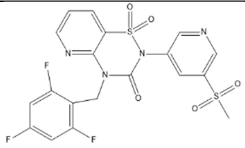	CHEMBL4592653	OX1R	Antagonist	6TQ6	6TOD
177	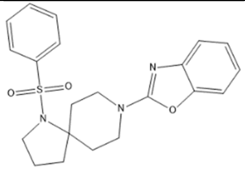	CHEMBL4590386	OX1R	Antagonist	6TQ4	6TOD
178	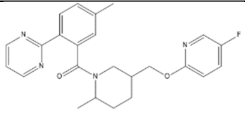	filorexant	OX1R	Antagonist	6TP6	6TOD
179	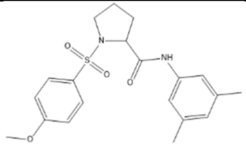	ACT-462206	OX1R	Antagonist	6TP4	6TOD
180	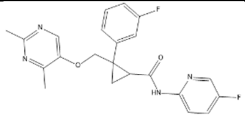	lemborexant	OX1R	Antagonist	6TOT	6TOD

181	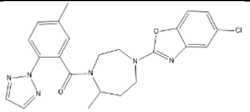	suvorexant	1. OX1R<br>2. OX2R	1. Antagonist<br>2. Antagonist	1. 4ZJ8, 6TO7<br>2. 4S0V, 6TPJ	1. 6TOD, 6TOD<br>2. 5WQC, 5WQC
182	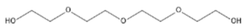	EMPA	1. OX1R<br>2. OX2R	1. Antagonist<br>2. Antagonist	1. 6TOD<br>2. 5WQC, 5WS3, 6TPG	1. 6TOS<br>2. 5WS3, 5WQC, 5WQC
183	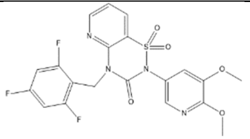	HTL6641	OX2R	Antagonist	6TPN	5WQC
184	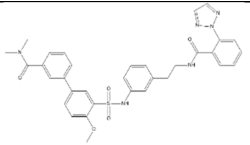	compound 1 [PMID: 33547286]	OX2R	Agonist	7L1V	7L1U
185	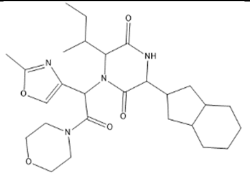	retosiban	OXYR	Antagonist	6TPK	7RYC
186	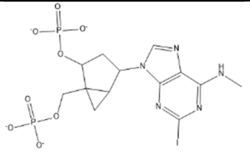	MRS2500	P2RY1	Antagonist	4XNW	4XNV
187	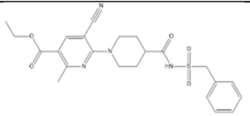	AZD1283	P2Y12	Antagonist	4NTJ	4PXZ
188	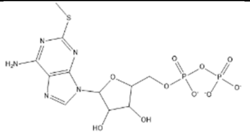	2MeSADP	P2Y12	Agonist	4PXZ	4NTJ
189	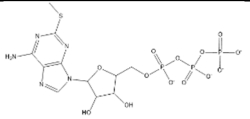	2MeSATP	P2Y12	Agonist	4PY0	4PXZ
190	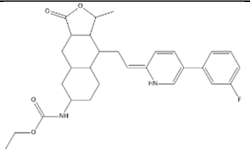	vorapaxar	PAR1	Antagonist	3VW7	7TD0

191	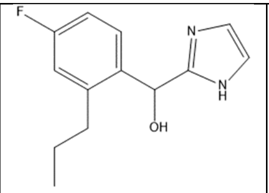	AZ8838	PAR2	Antagonist	5NDD	5NDZ
192	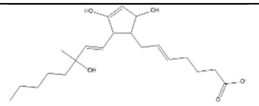	15(*R*)-15-methyl-prostaglandin D_2	PD2R2	Antagonist	7M8W	6D27
193	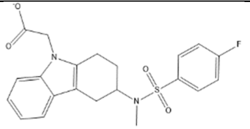	CHEMBL179036	PD2R2	Antagonist	6D27	7M8W
194	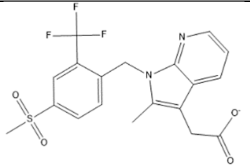	fevipiprant	PD2R2	Antagonist	6D26	7M8W
195	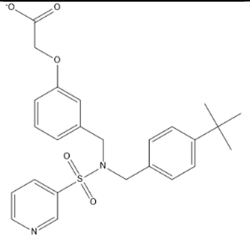	evatanepag	PE2R2	Agonist	7CX4	7CX3
196	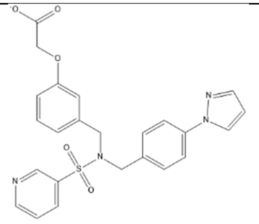	taprenepag	PE2R2	Agonist	7CX3	7CX2
197	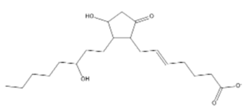	prostaglandin E_2	1. PE2R2<br>2. PE2R3<br>3. PE2R4	1. Agonist<br>2. Agonist<br>3. Agonist	1. 7CX2<br>2. 6AK3<br>3. 7D7M	1. 7CX3<br>2. 6M9T<br>3. 5YWY
198	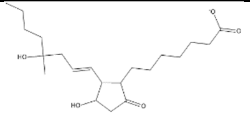	misoprostol-FA	PE2R3	Agonist	6M9T	6AK3

199	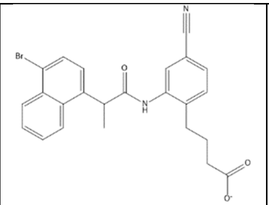	ONO-AE3-208-Br	PE2R4	Antagonist	5YHL	5YWY
200	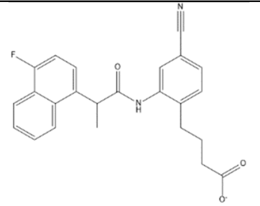	ONO-AE3-208	PE2R4	Antagonist	5YWY	5YHL
201	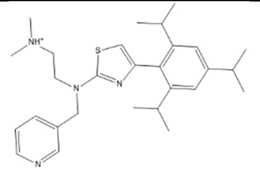	foropafant	PTAFR	Antagonist	5ZKP	5ZKQ
202	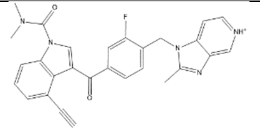	ABT-491	PTAFR	Inverse agonist	5ZKQ	5ZKP
203	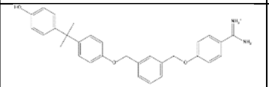	BIIL 260	Q9WTK1	Antagonist	5X33	NA
204	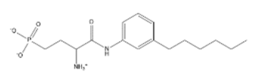	W146	1. S1PR1<br>2. S1PR1	1. Antagonist<br>2. Antagonist	1. 3V2W<br>1. 3V2Y	1. 3V2Y<br>1. 3V2W
205	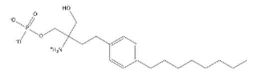	(S)-FTY720-phosphate	1. S1PR1<br>2. S1PR3	1. Agonist<br>2. Agonist	1. 7EO2<br>2. 7EW2	1. 7TD4<br>2. 7EW3
206	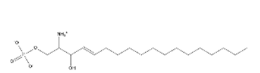	sphingosine 1-phosphate	1. S1PR1<br>2. S1PR3	1. Agonist<br>2. Agonist	1. 7WF7<br>2. 7C4S	1. 7TD4<br>2. 7EW2
207	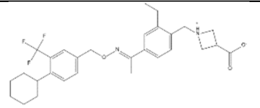	siponimod	1. S1PR1<br>2. S1PR5	1. Agonist<br>2. Agonist	1. 7EO4<br>2. 7EW1	1. 7TD4
208	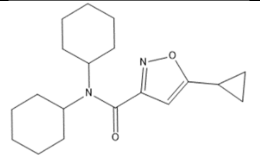	CYM-5541	S1PR3	Agonist	7EW4	7EW2
209	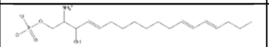	sphingosine 1-phosphate	S1PR3	Agonist	7EW3	7EW2

210	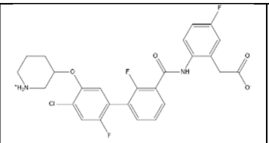	CHEMBL4751488	SUCR1	Antagonist	6Z10	6RNK
211	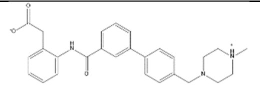	NF-56-EJ40	SUCR1	Antagonist	6RNK	6Z10
212	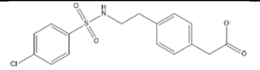	daltroban	TA2R	Antagonist	6IIV	6IIU
213	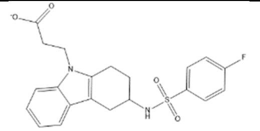	ramatroban	TA2R	Antagonist	6IIU	6IIV

Target names, ligand functions, self-dock PDBIDs, and cross-dock PBDIDs are numbered per target for ligands possessing activity for multiple targets.

[a]Structures are shown in the ionization state they were docked in. Additionally, structures labeled with 'Abs' denote the presence of a constrained stereocenter during docking. Stereocenters lacking this label were left unconstrained during docking.

[b]Entries assigned an NA value denote ligands that were not cross-docked.

Table S12. Anchor residues used in ECL2 modeling for each target studied.

	Target Receptor	Template Receptor	Template PDBID	Anchor Residue 1[a]	Anchor Residue 2[a]
Best Case[a]	AA2AR	AA1AR	7LD3[19]	L137	M174
	AA2AR	AA1AR	5UEN[16]	L137	M174
	ADRB1	ADRB2	4LDE[78]	L195	R222
	ADRB1	ADRB2	6PS2[45]	L195	R222
	ADRB2	ADRB1	7BU7[64]	Q170	Q197
	ADRB2	ADRB1	7BVQ[64]	Q170	Q197
	CXCR4	CCR1	7VL9[186]	F174	L194
	CXCR4	CCR9	5LWE[187]	L167	V198
	DRD3	DRD2	7JVR[112]	L169	P186
	DRD3	DRD2	6CM4[117]	F170	P186
Normal Case[b]	AA2AR	S1PR5	7EW1[182]	L141	P173
	ADRB1	5HT6	7XTB[188]	L195	R222
	ADRB2	DRD2	6LUQ[116]	I169	Q197
	CXCR4	AGTR2	5UNH[96]	I173	D193
	DRD3	5HT1D	7E32[1]	L169	P186

[a]Best case homology models represent cases where selected template receptors bind the same endogenous ligand as the target. In addition, best case homology models represent models constructed using both active and inactive state template structures.

[b]Normal case homology models represent cases where a selected template receptor does not bind the same endogenous ligand as the target.

[c]Anchor residues are numbered to match the numbering scheme present on GPCRdb.[189]
